# Systematics of testudacarine torrent mites (Acari, Hydrachnidia, Torrenticolidae) with descriptions of 13 new species from North America

**DOI:** 10.3897/zookeys.582.7684

**Published:** 2016-04-21

**Authors:** Joseph C. O’Neill, J. Ray Fisher, Whitney A. Nelson, Micheal J. Skvarla, Danielle M. Fisher, Ashley P. G. Dowling

**Affiliations:** 1 Department of Entomology, University of Arkansas, Fayetteville, AR 72701, USA

**Keywords:** Hydrachnidiae, Hydrachnidia, water mites, Testudacarinae, Testudacarus, Debsacarus

## Abstract

Thirteen new species of North American *Testudacarus* (Torrenticolidae: Testudacarinae) are described: *Testudacarus deceptivus* O’Neill & Dowling, **sp. n.**, *Testudacarus hitchensi* O’Neill & Dowling, **sp. n.**, *Testudacarus harrisi* O’Neill & Dowling, **sp. n.**, *Testudacarus dennetti* O’Neill & Dowling, **sp. n.**, *Testudacarus dawkinsi* O’Neill & Dowling, **sp. n.**, *Testudacarus radwellae* O’Neill & Dowling, **sp. n.**, *Testudacarus kirkwoodae* O’Neill & Dowling, **sp. n.**, *Testudacarus hyporhynchus* O’Neill & Dowling, **sp. n.**, *Testudacarus smithi* O’Neill & Dowling, **sp. n.**, *Testudacarus rollerae* O’Neill & Dowling, **sp. n.**, *Testudacarus elongatus* O’Neill & Dowling, **sp. n.**, *Testudacarus rectangulatus* O’Neill & Dowling, **sp. n.**, and *Testudacarus oblongatus* O’Neill & Dowling, **sp. n.** *Testudacarus vulgaris* Habeeb, 1954 is resurrected from synonymy with *Testudacarus minimus* and redescribed. *Debsacarus* (Habeeb, 1961), *Testudacarus americanus* Marshall, 1943, and *Testudacarus minimus* Marshall, 1943 are redescribed. All redescriptions are from original types. Species delimination was accomplished through examination of morphology, biogeography, and molecular phylogenetics of the barcoding region of COI. Other species are addressed and a key to world species is presented. For Testudacarinae, this represents the first published: 1) descriptions from multiple specimens (i.e. intraspecific variation); 2) colored photographs; 3) explicit illustrations and discussion of sexual dimorphism within the subfamily; 4) genetic data. A comprehensive testudacarine reference list is also included.

## Introduction

Torrenticolidae Piersig, 1902 are ubiquitous and diverse in North America, but the majority of species remain undescribed. This study is the second in a series of descriptions of North American torrenticolids. The goal of this ongoing taxonomic project is to explore the family and make these mites amenable to other researchers.

Testudacarinae Cook, 1974 are found abundantly in riffles of fast flowing streams throughout most of North America and sporadically in Asia. Typical of lotic-dwelling water mites, testudacarines are dorso-ventrally flattened, heavily sclerotized, and possess robust legs with large tarsal claws used for crawling. Most testudacarines are less than 1 mm in size and can exhibit striking coloration. Larvae are reported to be ectoparasites of chironomid adults (Smith 1982).

Despite their abundance, few testudacarines are described worldwide and in North America the most recent description is over fifty years old. Limited morphological and distributional data have been presented, and no genetic data has ever been published on Testudacarinae. Minimalistic and incomplete descriptions have led to considerable confusion throughout testudacarine taxonomic history. There is a need describe new species with modern methods and to redescribe older species with the same thoroughness.

Thirteen descriptions and four redescriptions of North American *Testudacarus* Walter, 1928 are included within. Following Fisher et al. (2015), species were delimited using a combination of morphology, biogeography, and molecular data (i.e. “barcoding” region of COI). In addition to descriptions and redescriptions, sexual dimorphism within the subfamily is explicitly addressed, a comprehensive testudacarine reference list is included, and a key to world species is presented.

## Taxonomic history

There are currently nine testudacarines described worldwide: *Testudacarus tripeltatus* Walter, 1928 from India; *Testudacarus japonicus* Imamura, 1955 and *Testudacarus okadai* Imamura, 1976 from Japan; *Testudacarus binodipalpis* Guo and Jin, 2005 from China; and *Testudacarus americanus* Marshall, 1943, *Testudacarus minimus* Marshall, 1943, *Testudacarus minimus vulgaris* (Habeeb, 1954), *Testudacarus americanus galloi* Habeeb, 1969, and *Debsacarus oribatoides* (Habeeb, 1961) from the United States. However, the status of several of these testudacarines remains unclear.

*Testudacarus americanus* and *Testudacarus minimus* were described by Marshall (1943) from one “small” male and one “large” female from the same creek in California. Habeeb (1954) described *Testudacarus vulgaris* from New Brunswick. Later, Habeeb (1967) synonymized *Testudacarus minimus* with *Testudacarus americanus* after noticing sexual dimorphism within *Testudacarus* (specifically, females are larger than males). Habeeb (1969) then synonymized *Testudacarus vulgaris* with *Testudacarus americanus* and established *Testudacarus americanus galloi*, from “two female mites rather like [*Testudacarus americanus*], yet atypical.” He stated that *Testudacarus americanus vulgaris* was a blue form found from New Brunswick to as far west as Arizona, and *Testudacarus americanus americanus* and *Testudacarus americanus minimus* were “red to golden” forms found from California. Habeeb (1974a) then resurrected *Testudacarus minimus* and changed *Testudacarus americanus vulgaris* to *Testudacarus minimus vulgaris*, after realizing he had misread Marshall (1943).

Habeeb (1961) described *Testudacarus oribatoides* from a male and female from California. This species has a “protrusable maxillary tube…reminiscent of *Pseudotorrenticola*,” and is in other respects atypical for *Testudacarus* (Habeeb 1961). Habeeb (1974b) erected *Debsacarus* and designated *Testudacarus oribatoides* as the type specimen “due to the fact that many recent authors have no respect for subgeneric names.”

Only two authors, Viets (1987) and Smith (1982), address the hypotheses proposed by Habeeb (1969, 1974a, 1974b). Viets (1987) did not take a stance on the validity of any species, instead he catalogued all the names presented in the literature and asked the reader to “vergl.” (short for the German vergleichen, or “compare”). However, concerning *Debsacarus*, Viets (1987) did state: “Diagnose und abbildungen dürftig; Genus- und Artberechtigung unklar,” (“Diagnosis and illustrations poor; genus and art authority unclear.”). Smith (1982) acknowledged that Habeeb (1969) “proposed a second subspecies from California,” but otherwise took no stance on its validity.

## Methods

### Sampling and curation

Mites were collected and preserved using protocols detailed in Fisher et al. (2015).

### Morphological terminology

**Figure 1.**
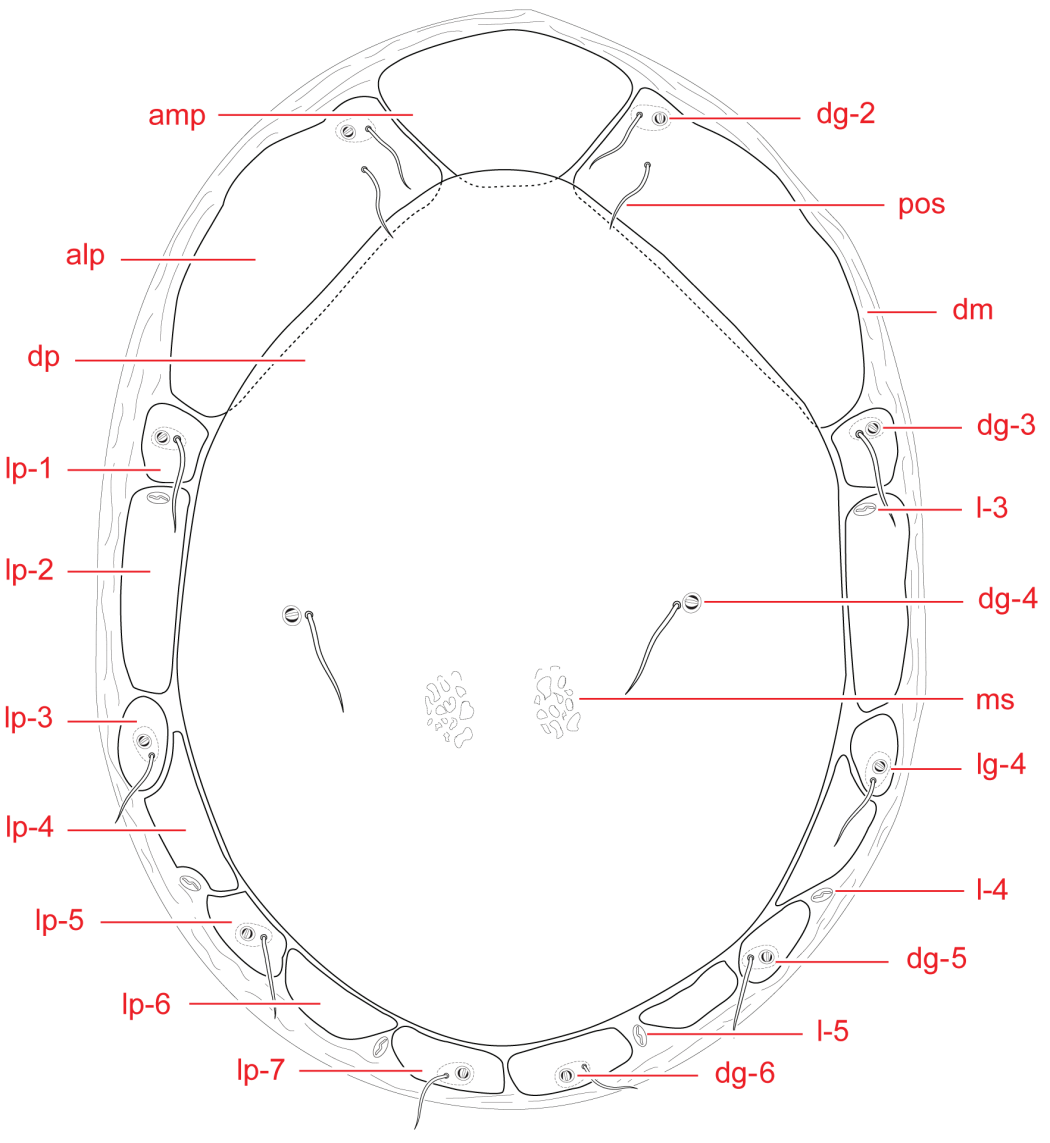
Testudacarine male dorsum (generalized): (**Left**) – anterio-medial platelet (amp); anterio-lateral platelet (alp); dorsal plate (dp); lateral platelets (lp); (**Right**) – dorso-glandularia (dg); post-ocularial setae (pos); dorsal membrane (dm); lyriffisures (l); muscle scars (ms); latero-glandularia (lg).

**Figure 2.**
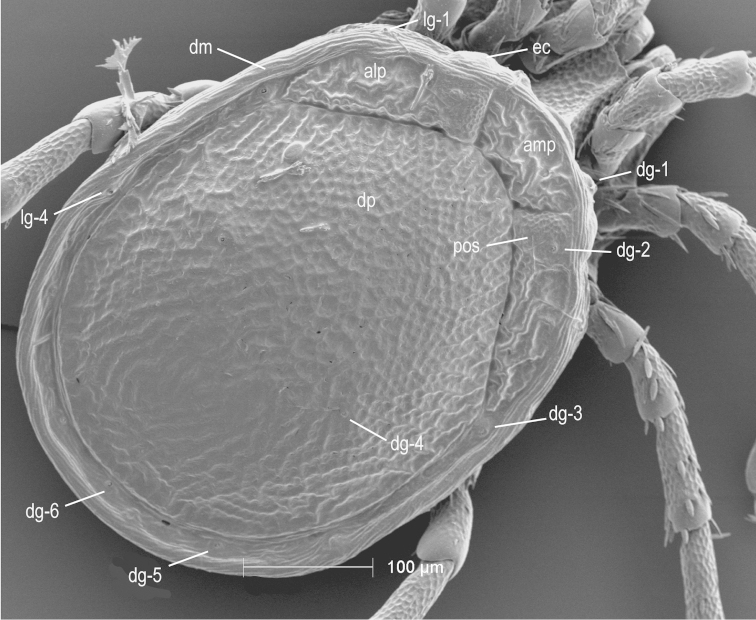
Testudacarine male dorsum (SEM): anterio-medial platelet (amp); anterio-lateral platelet (alp); dorsal plate (dp); dorso-glandularia (dg); post-ocularial setae (pos); dorsal membrane (dm); latero-glandularia (lg). Scale: 100 µm. Photo Michelle Hoppner and Ian Smith (used with permission).

**Figure 3.**
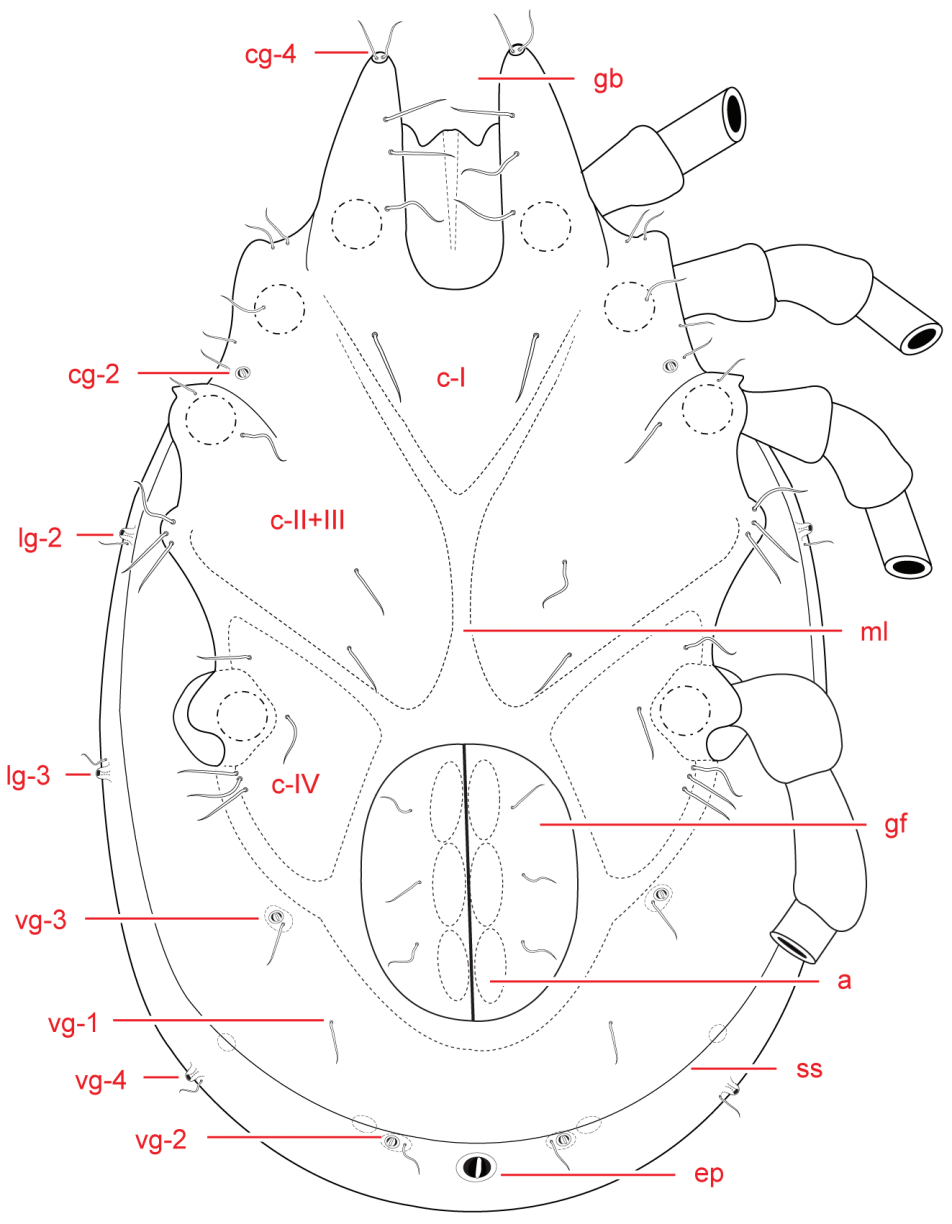
Testudacarine male venter (generalized): **Left** – coxo-glandularia (cg); latero-glandularia (lg); ventro-glandularia (vg); **Middle** – coxae (c). **Right** – gnathosomal bay (gb); coxae-II+III midline (ml); genital field (gf); acetabula (a); line of secondary sclerotization (ss); excretory pore (ep).

**Figure 4.**
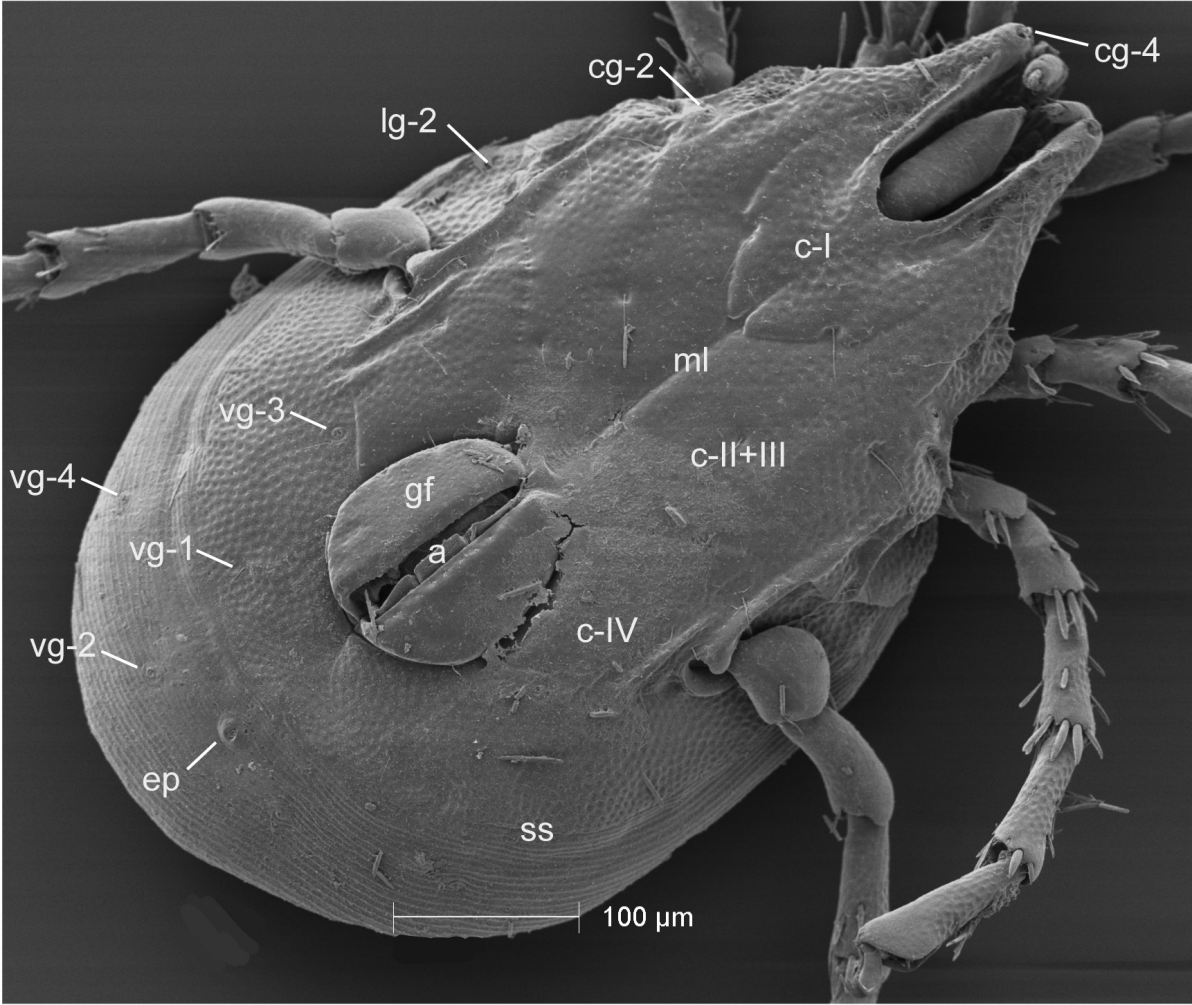
Testudacarine male venter (SEM): coxo-glandularia (cg); latero-glandularia (lg); ventro-glandularia (vg); coxae (c); coxae-II+III midline (ml); genital field (gf); acetabula (a); line of secondary sclerotization (ss); excretory pore (ep). Scale: 100 µm. Photo Michelle Hoppner and Ian Smith (used with permission).

**Figure 5.**
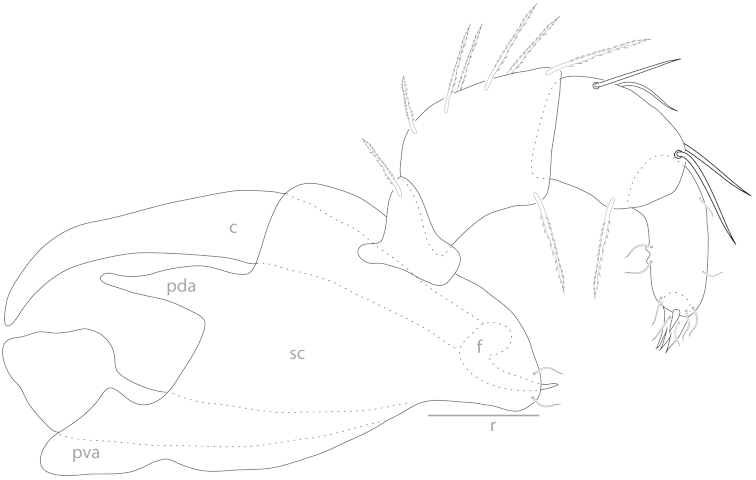
Testudacarine gnathosoma (generalized): chelicerae (c); posterio-dorsal apodeme (pda); posterio-ventral apodeme (pva); subcapitulum (sc); fang (f); rostrum (r).

Terminology used in this study is detailed in Figs 1–5 and follows Goldschmidt (2007) as modified by Fisher et al. (2015). Hyphens are used for directional or numbered morphological features: for example, dorsoglandularia 1 will be expressed as dorso-glandularia-1. This is to prevent confusion when terms are followed by numbers and to make longer, more complicated terminology more accessible to unfamiliar readers. “Colorless” refers to a lack of pigmentation in the cuticle; as the cuticle itself is typically yellowish, “colorless” species are thus yellowish.

### Images and measurements

Images were produced and measurements taken following the protocol detailed in Fisher et al. (2015). Measurements follow Goldschmidt (2007) with additions.

### Material deposition of Nearctic types

All holotypes, allotypes, and some paratypes have been deposited in the Canadian National Collection of Insects, Arachnids, and Nematodes (CNC), Ottawa, Canada. Additional paratypes have been deposited in the Acari Collection of the University of Arkansas (ACUA), Fayetteville, Arkansas. Specific numbers of slides deposited at the CNC and ACUA are noted within each species description. Collection abbreviations are used throughout.

### Morphological and distributional examinations

Material from the CNC and additional collections provided tens of thousands of testudacarines for morphological examination from across North America; a portion of these were examined closely for morphological variation. Previous torrenticolid studies suggested color and size were not necessarily important characters in distinguishing species (e.g., Fisher et al. 2015). Therefore, testudacarine “morphotypes” were chosen conservatively, giving more weight to drastic character differences, such as the presence of four instead of five pedipalp segments, over potentially more ambiguous characters, such as color and size variation. Many morphological characters were examined including general characteristics (e.g., color, size, body shape) and specific morphological features such as the following: shape of the dorsal plate, platelets, coxal field, and genital field; positioning of glandularia and lyrifissures; setae on the dorsum, venter, and gnathosoma; and structure of the gnathosoma and ejaculatory complex. Over 100 measurements per specimen were taken and compared and proportions between many of these measurements were analyzed. Finally, distributional data was considered for each “morphotype” and probable ranges were hypothesized. Differences and similarities in ranges were considered as further supporting evidence of putative species.

### Molecular examination

The “barcoding” region of COI was used as an independent test of morphological species hypotheses. COI was used to determine if any morphological characters, conservative or ambiguous, indicated species boundaries by sorting into distinct genetic lineages. COI was also used in the same way to test distributional hypotheses. Taxon sampling included roughly 300 specimens spanning “morphotypes” from across North America. Unfortunately, ethanol collections were limited from Mexico, northern Canada, and the eastern United States and therefore do not fully represent the ranges of species from these regions. Later, twenty specimens were included for phylogenetic analysis of 28S (D1-3) to investigate interspecific relationships. Genbank accession numbers of specimens for which sequences were obtained and used in this study are located in Table 1. Based upon recommendations by Chakrabarty et al. (2013), GenSeq nomenclature is used in the table to indicate the status of types and non-types sequenced.

**Table 1.** Genbank accession numbers and GenSeq nomenclature for each specimen sequenced for this study.

Species	Genbank Accession #		Specimen Catalog #	GenSeq Nomenclature
	COI	28S		
*Testudacarus vulgaris*	KU243701		ACUA135545 (non-type voucher)	genseq-4 COI
*Testudacarus vulgaris*	KU243702	KU243846	ACUA135544 (non-type voucher)	genseq-4 COI, 28S
*Testudacarus harrisi*	KU243703		ACUA135543 (paratype)	genseq-2 COI
*Testudacarus harrisi*	KU243704		ACUA146756 (paratype)	genseq-2 COI
*Testudacarus harrisi*	KU243705		ACUA138471 (paratype)	genseq-2 COI
*Testudacarus hitchensi*	KU243706		ACUA141898 (holotype)	genseq-1 COI
*Testudacarus hitchensi*	KU243707		ACUA138472 (paratype)	genseq-2 COI
*Testudacarus hitchensi*	KU243708		ACUA138473 (paratype)	genseq-2 COI
*Testudacarus dennetti*	KU243709		ACUA138469 (paratype)	genseq-2 COI
*Testudacarus dennetti*	KU243710		ACUA144021 (paratype)	genseq-2 COI
*Testudacarus vulgaris*	KU243711		ACUA138476 (non-type voucher)	genseq-4 COI
*Testudacarus dawkinsi*	KU243712		ACUA141897 (holotype)	genseq-1 COI
*Testudacarus vulgaris*	KU243713		ACUA138476 (non-type voucher)	genseq-4 COI
*Testudacarus vulgaris*	KU243714		ACUA138478 (non-type voucher)	genseq-4 COI
*Testudacarus vulgaris*	KU243715		ACUA141903 (non-type voucher)	genseq-4 COI
*Testudacarus vulgaris*	KU243716		ACUA138479 (non-type voucher)	genseq-4 COI
*Testudacarus vulgaris*	KU243717		ACUA141904 (non-type voucher)	genseq-4 COI
*Testudacarus vulgaris*	KU243718		ACUA138480 (non-type voucher)	genseq-4 COI
*Testudacarus vulgaris*	KU243719		ACUA138481 (non-type voucher)	genseq-4 COI
*Testudacarus vulgaris*	KU243720		ACUA138482 (non-type voucher)	genseq-4 COI
*Testudacarus vulgaris*	KU243721		ACUA141901 (non-type voucher)	genseq-4 COI
*Testudacarus vulgaris*	KU243722		ACUA141902 (non-type voucher)	genseq-4 COI
*Testudacarus vulgaris*	KU243723		ACUA141900 (non-type voucher)	genseq-4 COI
*Testudacarus vulgaris*	KU243724		ACUA138484 (non-type voucher)	genseq-4 COI
*Testudacarus minimus*	KU243725	KU243847	ACUA138487 (non-type voucher)	genseq-4 COI, 28S
*Testudacarus vulgaris*	KU243726		ACUA141899 (non-type voucher)	genseq-4 COI
*Testudacarus minimus*	KU243727		ACUA141905 (non-type voucher)	genseq-4 COI
*Testudacarus vulgaris*	KU243728		ACUA138486 (non-type voucher)	genseq-4 COI
*Testudacarus minimus*	KU243729		ACUA138488 (non-type voucher)	genseq-4 COI
*Testudacarus rectangulatus*	KU243730		ACUA138494 (holotype)	genseq-1 COI
*Testudacarus elongatus*	KU243731		ACUA138495 (holotype)	genseq-1 COI
*Testudacarus minimus*	KU243732		ACUA138489 (non-type voucher)	genseq-4 COI
*Testudacarus minimus*	KU243733		ACUA141906 (non-type voucher)	genseq-4 COI
*Testudacarus minimus*	KU243734		ACUA138490 (non-type voucher)	genseq-4 COI
*Testudacarus minimus*	KU243735		ACUA138491 (non-type voucher)	genseq-4 COI
*Testudacarus minimus*	KU243736		ACUA138492 (non-type voucher)	genseq-4 COI
*Testudacarus minimus*	KU243737		ACUA138493 (non-type voucher)	genseq-4 COI
*Testudacarus dennetti*	KU243738		ACUA143634 (paratype)	genseq-2 COI
*Testudacarus dennetti*	KU243739		ACUA141892 (paratype)	genseq-2 COI
*Testudacarus dennetti*	KU243740		ACUA141893 (paratype)	genseq-2 COI
*Testudacarus hitchensi*	KU243741	KU243848	ACUA141894 (paratype)	genseq-2 COI, 28S
*Testudacarus vulgaris*	KU243742	KU243850	ACUA142194 (non-type voucher)	genseq-4 COI, 28S
*Testudacarus harrisi*	KU243743		ACUA141896 (paratype)	genseq-2 COI
*Testudacarus harrisi*	KU243744		ACUA143618 (paratype)	genseq-2 COI
*Testudacarus hitchensi*	KU243745		ACUA143629 (paratype)	genseq-2 COI
*Testudacarus hitchensi*	KU243746		ACUA143633 (non-type voucher)	genseq-4 COI
*Testudacarus hitchensi*	KU243747		ACUA141895 (paratype)	genseq-2 COI
*Testudacarus harrisi*	KU243748		ACUA143619 (paratype)	genseq-2 COI
*Testudacarus harrisi*	KU243749		ACUA143623 (paratype)	genseq-2 COI
*Testudacarus kirkwoodae*	KU243750		ACUA141885 (holotype)	genseq-1 COI
*Testudacarus americanus*	KU243751		ACUA141886 (non-type voucher)	genseq-4 COI
*Testudacarus americanus*	KU243752	KU243849	ACUA141887 (non-type voucher)	genseq-4 COI, 28S
*Testudacarus americanus*	KU243753		ACUA142195 (non-type voucher)	genseq-4 COI
*Testudacarus elongatus*	KU243754		ACUA141888 (paratype)	genseq-2 COI
*Testudacarus elongatus*	KU243755	KU243851	ACUA141889 (paratype)	genseq-2 COI, 28S
*Testudacarus elongatus*	KU243756		ACUA142196 (paratype)	genseq-2 COI
*Testudacarus elongatus*	KU243757		ACUA142197 (paratype)	genseq-2 COI
*Testudacarus minimus*	KU243758		ACUA141890 (non-type voucher)	genseq-4 COI
*Testudacarus minimus*	KU243759		ACUA142198 (non-type voucher)	genseq-4 COI
*Testudacarus elongatus*	KU243760		ACUA142199 (paratype)	genseq-2 COI
*Testudacarus kirkwoodae*	KU243761	KU243852	ACUA142200 (non-type voucher)	genseq-4 COI, 28S
*Testudacarus minimus*	KU243762		ACUA141891 (non-type voucher)	genseq-4 COI
*Testudacarus vulgaris*	KU243763		ACUA143643 (non-type voucher)	genseq-4 COI
*Testudacarus vulgaris*	KU243764		ACUA143644 (non-type voucher)	genseq-4 COI
*Testudacarus dennetti*	KU243765		ACUA143645 (holotype)	genseq-1 COI
*Testudacarus vulgaris*	KU243766		ACUA143646 (non-type voucher)	genseq-4 COI
*Testudacarus vulgaris*	KU243767		ACUA143647 (non-type voucher)	genseq-4 COI
*Testudacarus harrisi*	KU243768	KU243853	ACUA143648 (paratype)	genseq-2 COI, 28S
*Testudacarus dennetti*	KU243769		ACUA143649 (paratype)	genseq-2 COI
*Testudacarus deceptivus*	KU243770	KU243854	ACUA143652 (holotype)	genseq-1 COI, 28S
*Testudacarus oribatoides*	KU243771	KU243855	ACUA143654 (non-type voucher)	genseq-4 COI, 28S
*Testudacarus vulgaris*	KU243772		ACUA143655 (non-type voucher)	genseq-4 COI
*Testudacarus minimus*	KU243773		ACUA143657 (non-type voucher)	genseq-4 COI
*Testudacarus vulgaris*	KU243774		ACUA143658 (non-type voucher)	genseq-4 COI
*Testudacarus vulgaris*	KU243775		ACUA143659 (non-type voucher)	genseq-4 COI
*Testudacarus vulgaris*	KU243776		ACUA143661 (non-type voucher)	genseq-4 COI
*Testudacarus minimus*	KU243777		ACUA143664 (non-type voucher)	genseq-4 COI
*Testudacarus minimus*	KU243778	KU243856	ACUA143665 (non-type voucher)	genseq-4 COI, 28S
*Testudacarus deceptivus*	KU243779		ACUA143666 (paratype)	genseq-2 COI
*Testudacarus vulgaris*	KU243780	KU243857	ACUA143667 (non-type voucher)	genseq-4 COI, 28S
*Testudacarus vulgaris*	KU243781		ACUA143669 (non-type voucher)	genseq-4 COI
*Testudacarus vulgaris*	KU243782		ACUA143671 (non-type voucher)	genseq-4 COI
*Testudacarus minimus*	KU243783		ACUA146717 (non-type voucher)	genseq-4 COI
*Testudacarus minimus*	KU243784		ACUA146718 (non-type voucher)	genseq-4 COI
*Testudacarus minimus*	KU243785		ACUA146719 (non-type voucher)	genseq-4 COI
*Testudacarus minimus*	KU243786		ACUA146720 (non-type voucher)	genseq-4 COI
*Testudacarus minimus*	KU243787		ACUA146721 (non-type voucher)	genseq-4 COI
*Testudacarus vulgaris*	KU243788		ACUA146722 (non-type voucher)	genseq-4 COI
*Testudacarus vulgaris*	KU243789		ACUA146723 (non-type voucher)	genseq-4 COI
*Testudacarus rollerae*	KU243790		ACUA146727 (paratype)	genseq-2 COI
*Testudacarus rollerae*	KU243791	KU243858	ACUA146724 (paratype)	genseq-2 COI, 28S
*Testudacarus rollerae*	KU243792		ACUA146725 (holotype)	genseq-1 COI
*Testudacarus oblongatus*	KU243793		ACUA146726 (paratype)	genseq-2 COI
*Testudacarus oblongatus*	KU243794		ACUA146728 (holotype)	genseq-1 COI
*Testudacarus minimus*	KU243795		ACUA146729 (non-type voucher)	genseq-4 COI
*Testudacarus dennetti*	KU243796		ACUA146732 (paratype)	genseq-2 COI
*Testudacarus minimus*	KU243797		ACUA146733 (non-type voucher)	genseq-4 COI
*Testudacarus minimus*	KU243798		ACUA146734 (non-type voucher)	genseq-4 COI
*Testudacarus minimus*	KU243799		ACUA146735 (non-type voucher)	genseq-4 COI
*Testudacarus dawkinsi*	KU243800		ACUA146736 (paratype)	genseq-2 COI
*Testudacarus harrisi*	KU243801		ACUA146738 (paratype)	genseq-2 COI
*Testudacarus harrisi*	KU243802		ACUA146737 (paratype)	genseq-2 COI
*Testudacarus minimus*	KU243803		ACUA146739 (non-type voucher)	genseq-4 COI
*Testudacarus harrisi*	KU243804		ACUA146740 (paratype)	genseq-2 COI
*Testudacarus dawkinsi*	KU243805		ACUA146742 (paratype)	genseq-2 COI
*Testudacarus dawkinsi*	KU243806	KU243859	ACUA146743 (paratype)	genseq-2 COI, 28S
*Testudacarus dawkinsi*	KU243807		ACUA146744 (paratype)	genseq-2 COI
*Testudacarus dawkinsi*	KU243808		ACUA146745 (paratype)	genseq-2 COI
*Testudacarus dennetti*	KU243809		ACUA146746 (paratype)	genseq-2 COI
*Testudacarus harrisi*	KU243810		ACUA146747 (paratype)	genseq-2 COI
*Testudacarus harrisi*	KU243811		ACUA146748 (paratype)	genseq-2 COI
*Testudacarus minimus*	KU243812		ACUA146749 (non-type voucher)	genseq-4 COI
*Testudacarus harrisi*	KU243813		ACUA146750 (paratype)	genseq-2 COI
*Testudacarus hitchensi*	KU243814		ACUA146751 (paratype)	genseq-2 COI
*Testudacarus harrisi*	KU243815		ACUA146752 (holotype)	genseq-1 COI
*Testudacarus harrisi*	KU243816		ACUA146753 (paratype)	genseq-2 COI
*Testudacarus hitchensi*	KU243817		ACUA146754 (non-type voucher)	genseq-4 COI
*Testudacarus hitchensi*	KU243818		ACUA146755 (paratype)	genseq-2 COI
*Testudacarus hitchensi*	KU243819		ACUA146756 (paratype)	genseq-2 COI
*Testudacarus hitchensi*	KU243820		ACUA146757 (paratype)	genseq-2 COI
*Testudacarus hitchensi*	KU243821		ACUA146758 (non-type voucher)	genseq-4 COI
*Testudacarus dawkinsi*	KU243822		ACUA146759 (paratype)	genseq-2 COI
*Testudacarus minimus*	KU243823		ACUA146760 (non-type voucher)	genseq-4 COI
*Testudacarus hyporhynchus*	KU243824		ACUA146762 (holotype)	genseq-1 COI
*Testudacarus hyporhynchus*	KU243825	KU243860	ACUA146763 (paratype)	genseq-2 COI, 28S
*Testudacarus hyporhynchus*	KU243826		ACUA146764 (paratype)	genseq-2 COI
*Testudacarus americanus*	KU243827		ACUA146768 (non-type voucher)	genseq-4 COI
*Testudacarus smithi*	KU243828	KU243861	ACUA146769 (holotype)	genseq-1 COI, 28S
*Testudacarus smithi*	KU243829		ACUA146770 (paratype)	genseq-2 COI
*Testudacarus smithi*	KU243830		ACUA146772 (paratype)	genseq-2 COI
*Testudacarus oblongatus*	KU243831		ACUA146774 (paratype)	genseq-2 COI
*Testudacarus oblongatus*	KU243832		ACUA146775 (paratype)	genseq-2 COI
*Testudacarus oblongatus*	KU243833		ACUA146776 (paratype)	genseq-2 COI
*Testudacarus oblongatus*	KU243834		ACUA146777 (paratype)	genseq-2 COI
*Debsacarus oribatoides*	KU243835		ACUA146778 (non-type voucher)	genseq-4 COI
*Debsacarus oribatoides*	KU243836		ACUA146779 (non-type voucher)	genseq-4 COI
*Debsacarus oribatoides*	KU243837		ACUA146780 (non-type voucher)	genseq-4 COI
*Debsacarus oribatoides*	KU243838	KU243862	ACUA146781 (non-type voucher)	genseq-4 COI, 28S
*Debsacarus oribatoides*	KU243839		ACUA146778 (non-type voucher)	genseq-4 COI
*Testudacarus oblongatus*	KU243840	KU243863	ACUA146782 (paratype)	genseq-2 COI, 28S
*Testudacarus oblongatus*	KU243841		ACUA146783 (paratype)	genseq-2 COI
*Testudacarus dennetti*	KU243842	KU243864	ACUA146784 (paratype)	genseq-2 COI, 28S
*Testudacarus minimus*	KU243843		ACUA146785 (non-type voucher)	genseq-4 COI
*Testudacarus minimus*	KU243844		ACUA146786 (non-type voucher)	genseq-4 COI
*Testudacarus vulgaris*	KU243845		No voucher	No classification

Genomic DNA extraction was completed with Qiagen DNeasy Tissue Kits (Qiagen Inc.,Valencia, California). Amplifications of the target region of COI were performed with LCO1490 and HCO2198 (Folmer et al. 1994). Amplifications of the target region of 28S were performed with D23F and D6R (Park and Ó Foighill 2000). PCR was performed in a DNA Engine Peltier thermal cycler. COI samples were denatured for two minutes at 94 °C, followed by forty cycles of fifty seconds at 94 °C, thirty seconds at 48 °C, and one minute at 72 °C, with a final ten minute extension on the last cycle. 28S samples were denatured for two minutes and thirty seconds at 94 °C, followed by forty cycles of thirty seconds at 94 °C, twenty seconds at 53 °C, and one minute at 72 °C, with a final ten minute extension on the last cycle. Purification was done with Qiagen QIAquick PCR Purification Kits and test gels of 1.5% agarose were used to confirm PCR product quality. The purified product was then sequenced by Macrogen USA, based in Rockville, Maryland (http://macrogenusa.com/). DNASTAR© Lasergene SeqMan (Madison, Wisconsin) was used to reconcile forward and reverse sequences. The contigs that resulted were examined for contamination with GenBank BLAST searches. Clustal X (Thompson et al. 1997) was used to align sequences, and then BioEdit (Hall 1999) was used to conservatively edit the resulting sequences. COI sequences were around 650bp and 28S sequences were around 800bp. MrBayes (3.2.2) was used to perform Bayesian analyses over 5 million generations with *Lebertia* Neuman, 1880 as an outgroup. Monophyly was tested across Torrenticolidae as part of a forthcoming study. Molecular analysis was performed with the Extreme Science and Engineering Discovery Environment infrastructure available through the Cipres Portal (Miller et al. 2010).

### Species delimitation results

**Figure 6.**
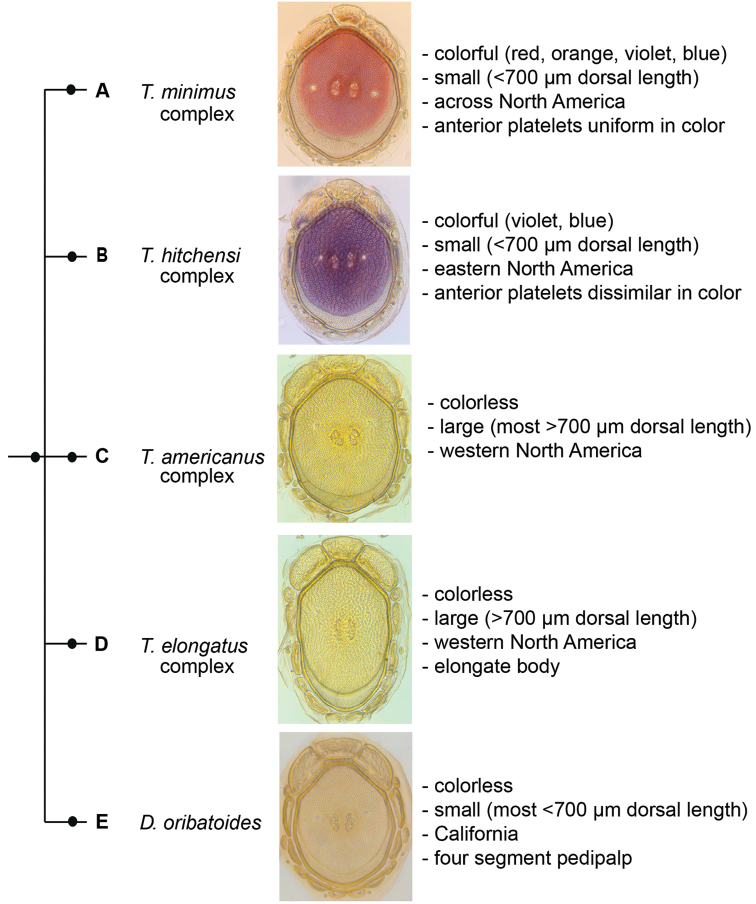
Testudacarinae molecular phylogeny and species complexes: (**Left**) combined 28S and COI Bayesian analysis resulting in a five branched soft polytomy (●: >95% posterior probability); monophyly tested across Torrenticolidae but not depicted; (**A–E** represent tree continuation in Figs 8, 12, 23, 32, and 43 respectively; (**Right**) species complexes with illustrative descriptions.

Phylogenetic analysis of COI and 28S resulted in five well-supported (posterior probability greater than 95%) clades; however, analyses did not produce resolution at the base of Testudacarinae, resulting in a five-branched polytomy (Fig. 6). Each of the five lineages show at least 15% COI divergence from another. Within these five lineages are 16 distinct and well-supported species. With few exceptions, these species exhibited relatively high COI divergence (greater than 5%) between clades and relatively low divergence within a given clade (less than 1.5%). Genetic extractions were unsuccessful for a 17^th^ species, *Testudacarus radwellae*.

Three morphotypes (*Testudacarus minimus*, *Testudacarus hitchensi*, and *Testudacarus elongatus*) exhibited more intraspecific variation than expected, suggesting potential cryptic species. Further investigation of specimens identified morphological and biogeographic differences suggesting three *Testudacarus minimus*-like species, four *Testudacarus hitchensi*-like species, and three *Testudacarus elongatus*-like species. However, some of these “species” exhibit high intraspecies COI divergence with restricted geographic ranges and no diagnosable morphological variability, and should be the target of further research.

In summary, we find strong support through a combination of morphology, biogeography, and phylogenetic analysis of COI and 28S for 17 species sorted into four robustly supported species complexes. The following species complexes are proposed to better organize the subfamily: *Testudacarus minimus* complex, *Testudacarus hitchensi* complex, *Testudacarus americanus* complex, and *Testudacarus elongatus* complex. Each complex is treated below within the taxonomic descriptions.

### Key to Testudacarinae species complexes:

**Table:** 

1	Pedipalp four-segmented, anterior tips of coxae-I with projections	***Debsacarus oribatoides***
–	Pedipalp five-segmented, anterior tips of coxae-I without projections	**2**
2	Body elongate to rectangular	***Testudacarus elongatus* complex**
–	Body oval	**3**
3	Body large (>700 µm female and >650 male dorsal length), dull coloration common; within and west of the Rocky Mountains	***Testudacarus americanus* complex (except *Testudacarus rollerae*)**
–	Body small (<700 µm female and <650 male dorsal length), bright coloration (orange, red, violet, blue) common; present throughout North America	(*Testudacarus minimus* complex, *Testudacarus hitchensi* complex, *Testudacarus rollerae*)...**4**
4	Anterio-medial platelet wide (>140 µm) and more than or nearly twice as wide as long	***Testudacarus rollerae***
–	Anterio-medial platelet unmodified (<140 µm) and far less than twice as wide as long	**5**
5	Anterio-medial and anterio-lateral platelets with consistent coloration (either colored or colorless across)	***Testudacarus minimus* complex**
–	Anterio-lateral platelets with coloration and anterio-medial platelet colorless	***Testudacarus hitchensi* complex**

## Taxonomy

### Torrenticolidae

Taxon classificationAnimaliaTrombidiformesTorrenticolidae

Piersig, 1902

http://zoobank.org/F4D093F6-B225-4E9B-999E-9956A9866564

#### Note.

See Fisher et al. (2015) for diagnosis.

### Testudacarinae

Taxon classificationAnimaliaTrombidiformesTorrenticolidae

Cook, 1974

http://zoobank.org/82730C11-1A78-4B39-8F74-6AD8ACF83A04

Testudacarinae Cook 1974: 145–146; Imamura 1976: 279; Fuste 1980: H7; Viets 1987: 222, 724; Bader 1988: 90; Smith and Cook 1991: 529, 552, 564–565, 574, 582; Cramer 1992: 13–14; Wiles 1997a: 192, 194, 199–200, 205, 209; Harvey 1998: 67; Smith and Cook 1999: 115; Smith et al. 2001: 579, 592, 608, 625, 645; Guo and Jin 2005: 70; Abé 2005: 120; Abé 2006: 6; Davids et al. 2007: 243; Goldschmidt 2007: 444; Boyaci and Özkan 2008: 364; Walter et al. 2009: 264; Zhang and Guo 2010: 117–118; Jin et al. 2010: 111; Smith et al. 2010: 492, 522, 535, 550, 566; Guo and Zhang 2011: 46, 48–49; Esen and Erman 2014: 39; Proctor et al. 2015: 622; Fisher et al. 2015: 83–84.

#### Subfamilial diagnosis.

For larval diagnosis see Smith (1982). Adults differ from torrenticolines in having three pairs of acetabula (six in Torrenticolinae); condyles present over the insertions of leg-IV; long posterio-dorsal subcapitular apodemes (also long in *Monatractides*); a ridge extending anteriorly from the leg-IV socket; and a ring of platelets closely affiliated with the central dorsal plate, i.e., not hidden within a dorsal furrow as in torrenticolines (Fig. 1). They are further characterized by having a single anterio-medial dorsal platelet and pedipalps without ventral projections, although some torrenticolines also have these characters. Testudacarinae can be further diagnosed by the following combination of characters. Medial dorsal plate exhibiting secondary and occasionally tertiary sclerotization. Dorsal platelets variable in size, shape, and coloration. Anterio-medial platelet smaller than anterio-lateral platelets and trapeziform (rounded to rectangular). Anterio-lateral platelets long with anterior bulge and posterior tapering. Seven pairs of lateral platelets present. Lateral-platelet-2, -4, and -6 large and elongate and -1, -3, -5, and -7 smaller and rounded. Lateral-platelet-3 highly variable and positioned either anterior or lateral to lateral-platelet-4. Lateral-platelet-4 highly variable in shape mostly depending on lateral-platelet-3 position. Dorso-glandularia-2 and post-ocularial setae located together on anterio-lateral platelet. Dorso-glandularia-3, -5, and -6 located on lateral-platelet-1, -5, and -7 respectively. Dorso-glandularia-4 located on the large medial dorsal plate. Latero-glandularia-4 located on lateral-platelet-3. Ventro-glandularia-3 posterior to coxae-IV (on coxae-IV in other torrenticolids). Coxo-glandularia-4 located at tip of coxae-I (as in *Monatractides* and many *Torrenticola*). Pedipalp, femur, and genu with plumose setae ventrally. Also similar to *Monatractides*, posterio-dorsal subcapitular apodemes are long. Rostrum short.

#### Distribution.

Testudacarines have been reported on many occasions outside of their original descriptions. Furthermore, the Canadian National Collection in Ottawa, Canada includes thousands of testudacarines collected from across most of North America (Smith et al. 2010). In Asia there have only been a handful of additional reports (Walter 1929, Pešić and Smit 2007, Jin et al. 2010, Morimoto 2012). This is not completely due to a lack of torrenticolid work in Asia, for an extensive list see Walter et al. (2009, pg. 256) and Fisher et al. (2015). Extensive work has also been done on water mites in Europe, Africa, and Australia without any reports of testudacarines. Therefore, testudacarines are currently thought to be widely distributed throughout most of North America (with southern limits in Mexico and northern limits around the 60^th^ parallel), and sparsely distributed in parts of Asia.

#### Remarks.

**Figure 7.**
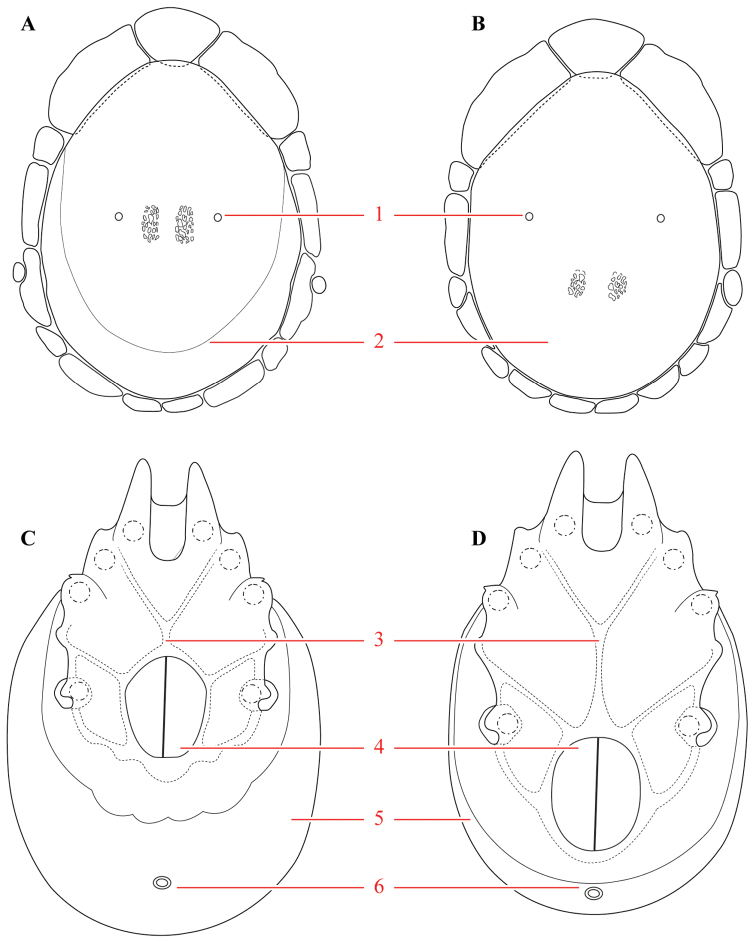
Testudacarine sexual dimorphism: female dorsal shield (**A**) and ventral shield (**C**) differing from male (**B, D**) by the following characters: 1) dorso-glandularia-4 positioned far closer to muscle scares; 2) area of secondary sclerotization always present (males rarely present; very small if present); 3) with shorter coxae-II+III midline; 4) genital field enveloped by coxal field; 5) larger and rounder body (males around 80% of female size); 6) excretory pore well separated from ventral line of secondary sclerotization.

The three pairs of acetabula, coxae-IV condyles, and “generalized” pedipalps are plesiomorhphic states that clearly show testudacarines as retaining ancestral torrenticolid characteristics (Wiles 1997a). Wiles (1997a) and other authors suggest latero-glandularia-3 is present on the dorsum of testudacarines. However, we suggest that this is latero-glandularia-4 due to its posterior-most positioning. We also detail sexually dimorphic characters (Fig. 7). Although Habeeb (1954) first noted differences between the sexes of *Testudacarus vulgaris*, he did not present these distinctions in their wider context as overall conditions of Testudacarinae. Sexual dimorphism present in Testudacarinae include: 1) female dorso-glandularia-4 positioned closer to the muscle scars; 2) dorsal secondary sclerotization always present in females and usually absent in males (very small if present in males); 3) female coxae-II+III midline short; 4) genital field almost entirely enveloped by coxal field in females but only around half of male genital field within coxal field; 5) females idiosoma larger and rounder (males around 80% of female size) with less of the ventral shield composed of coxal field; and 6) excretory pore well separated from ventral line of secondary sclerotization in females, and is either in direct contact with or nearly so in males.

### Debsacarus

Taxon classificationAnimaliaTrombidiformesTorrenticolidae

Habeeb, 1974

http://zoobank.org/9C344329-32F6-4C4B-8167-196E030B2ED8

Debsacarus Habeeb 1974b: 1; Viets 1987: 222, 724; Zhang and Guo 2010: 117.

#### Type species.

*Debsacarus oribatoides* (Habeeb, 1961).

#### Generic diagnosis.

*Debsacarus* differ from all other Testudacarinae in having four-segmented pedipalps (instead of five) and projections on the anterio-tips of coxae-I. With the exception of *Testudacarus hyporhynchus*, *Debsacarus* differ from all other Testudacarinae in having an elongate gnathosoma and an extremely narrow gnathosomal bay that is covered dorsally and ends anterior to the leg-I insertion ventrally.

#### Distribution.

Known from only two counties (Los Angeles and Monterey) in California.

### Debsacarus oribatoides

Taxon classificationAnimaliaTrombidiformesTorrenticolidae

(Habeeb, 1961)

http://zoobank.org/7749B09F-CA26-416A-8FE7-F445A5451B85

Debsacarus oribatoides : Habeeb 1974b: 1; Viets 1987: 222, 724.
Testudacarus oribatoides : Habeeb 1961: 5–6; Lundblad 1967: 418; Habeeb 1967: 4; Habeeb 1969: 2; Viets 1987: 222, 724.

#### Type series.

**Lectotype (1♀): California, USA**: 1♀ from Los Angeles County, Coldbrook Guard Station, North Fork of San Gabriel River, 25 June 1961, by H Habeeb, HH610024; **Paralectotype (1♂): California, USA**: 1♂ from Los Angeles County, Coldbrook Guard Station, North Fork of San Gabriel River, 25 June 1961, by H Habeeb, HH610024.

#### Other material examined.

**Other (10♀, 8♂): California, USA**: 1♂ from Monterey County, Salmon Falls Creek, beside Route 1 12.5 km south of Gorda (35°48'56.00"N, 121°21'30.00"W), 2 June 2010, by IM Smith, IMS100045; 5♀ and 3♂ from Monterey County, Los Padres National Forest, Lucia beside Ferguson-Nacimiento Road 5.6 km east of Route 1 (36°0'3.00"N, 121°28'31.00"W), 3 June 2010, by IM Smith, IMS100048; 1♀ and 3♂ from Monterey County, Los Padres National Forest, Lucia beside Nacimiento-Ferguson Road 11.3 km west of Nacimiento Campground (36°1'N, 121°27'W), 30 July 1987, by IM Smith, IMS8700119; 1♀ and 1♂ from Monterey County, Los Padres National Forest, Salmon Creek, beside Route 1 south of Gorda (35°49'N, 121°22'W), 29 July 1987, by IM Smith, IMS870118; 1♀ from Monterey County, Limekiln State Park, Hare Canyon Creek, near campground (36°0'41.00"N, 121°31'1.00"W), 6 September 2013, by JR Fisher, JRF13-0906-001; 1♀ from Monterey County, Salmon Creek, beside Route 1 south of Gorda (35°49'N, 121°22'W), 28 July 1987, by IM Smith, IMS870114A; 1♀ from Los Angeles County, Angeles National Forest, North Fork of San Gabriel River, off Route 39 (34°16'16.00"N, 117°50'46.00"W), 8 September 2013, by JR Fisher, JRF13-0908-001.

#### Type deposition.

Lectotype (1♀), and paralectotype (1♂) deposited at CNC.

#### Redescription.

**Female (n=11)** with characteristics of the genus with following specifications.

Gnathosoma (Fig. 9) — Subcapitulum [260–290 ventral length; 125–145 dorsal length; 73–84 tall] elongate with long rostrum. Chelicerae [195–220 long] noticeably straight with short, almost straight fangs [28–33 long]. Pedipalp [217–234 long] highly modified: lanceolate and with four segments. Trochanter [7–9 long; 38–40 wide] shortened. Femur [39–44 long; 30–34 wide]. Fused genu and tibia [41–47 long; 25–28 wide]. Tarsus [17–20 long; 12–15 wide].

Dorsum (Fig. 10) — [574–741 long; 471–561 wide] round to ovoid. Dorsal plate [465–586 long; 391–451 wide]. Primary sclerotization [436–510 long] grey-blue. Dorso-glandularia-4 [163–194 apart] in line with and lateral to [29-48] muscle scars. Platelets extremely robust and colorless. All three anterior platelets similar in size and noticeably rectangular. Anterio-medial platelet [173–209 long; 74–128 wide] large trapezoid with slightly rounded anterior margin. Anterio-lateral platelet [185–207 long; 97–127 wide] without noticeable bulge or posterior narrowing. Lateral platelets as follows: lateral-1 [38–50 long; 25–38 wide]; lateral-2 [143–172 long; 40–66 wide]; lateral-3 [39–64 long; 16–32 wide]; lateral-4 [107–132 long; 28–51 wide]; lateral-5 [51–78 long; 28–48 wide]; lateral-6 [92–128 long; 25–55 wide]; lateral-7 [49–101 long; 22–50 wide].

**Figure 8.**
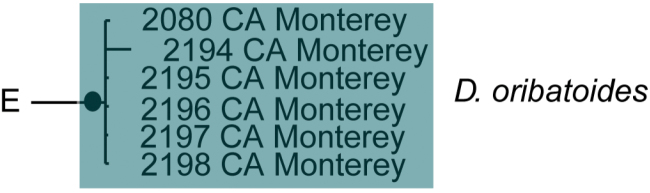
*Debsacarus oribatoides* molecular phylogeny: 28S and COI Bayesian analysis showing strong support single distinct clade (●: >95% posterior probability); clade exhibits <.6% divergence in COI within and >15% divergence between any other clade (not pictured); continuation of (**E**) lineage from Fig. 6.

**Figure 9.**
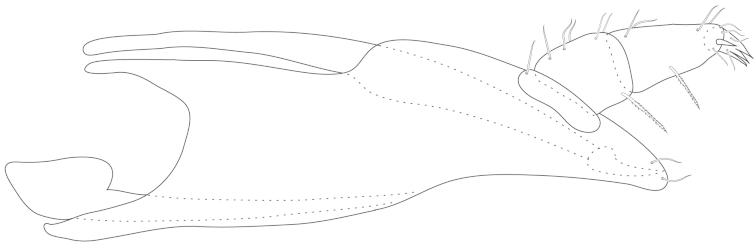
*Debsacarus oribatoides* gnathosoma (generalized).

**Figure 10.**
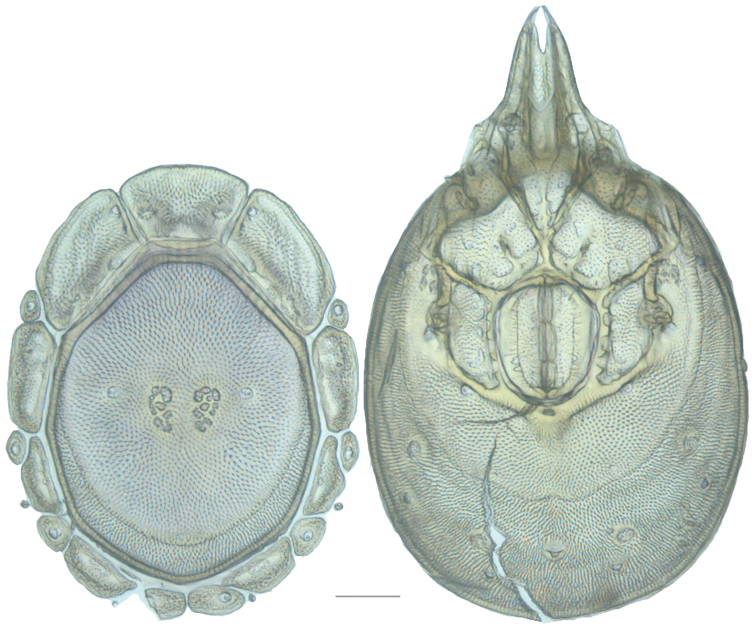
*Debsacarus oribatoides* female: (**Left**) dorsum; (**Right**) venter. Scale: 100 µm.

Venter (Fig. 10) — [779–929 long; 510–610 wide] round to ovoid and colorless. Primary sclerotization [668–756 long. Gnathosomal bay [33–45 dorsal length; 128–148 ventral length; 33–38 wide] very narrow; dorsal bay length extremely short giving the bay a “covered” appearance and ventral bay base ending anterior to the leg-I insertion. Coxal field [520–567 long; 325–353 wide]. Coxa-I [292–334 long; 160–186 midlength] long and with characteristic secondary growth attached at the anterior tips. Coxa-II + III [137–153 distance to top of coxa-II; 210–237 distance to top of coxa-III; 379–424 distance to bottom of coxa-III; 228–274 total length]. Coxa-IV [355–400 distance to top; 155–173 total length]. Genital field [362–409 distance to top; 556–601 distance to bottom; 185–208 total length; 155–175 width; 221–274 distance from gnathosomal bay; 59–101 distance from coxa-I; 163–227 distance to excretory pore; 215–366 distance to caudad] large. Eggs [200 long; 1–2 eggs]. Distance to excretory pore [727–809].

Legs — colorless. Total leg and podomere lengths as follows: Leg-I [459–505 total; trochanter 54–62; basifemur 81–91; telofemur 63–68; genu 81–91; tibia 86–100; tarsus 84–95]. Leg-II [516–554 total; trochanter 62–65; basifemur 85–100; telofemur 63–71; genu 84–96; tibia 100–114; tarsus 106–115]. Leg-III [593–644 total; trochanter 63–69; basifemur 97–105; telofemur 70–78; genu 104–118; tibia 125–143; tarsus 130–142]. Leg-IV [779–862 total; trochanter 84–96; basifemur 118–127; telofemur 115–129; genu 141–166; tibia 160–181; tarsus 148–170].

**Male (n=9)** similar to female except for sexually dimorphic characters previously discussed and with following specifications.

Gnathosoma (Fig. 9) — Subcapitulum [229–266 ventral length; 120–132 dorsal length; 64–78 tall]. Chelicerae [175–200 long]. Fangs [25–26 long]. Pedipalp [206–219 long]. Trochanter [7–9 long; 35–38 wide]. Femur [36–40 long; 30–32 wide]. Fused genu and tibia [43–45 long; width 23–26 wide]. Tarsus [16–17 long; 13–15 wide].

**Figure 11.**
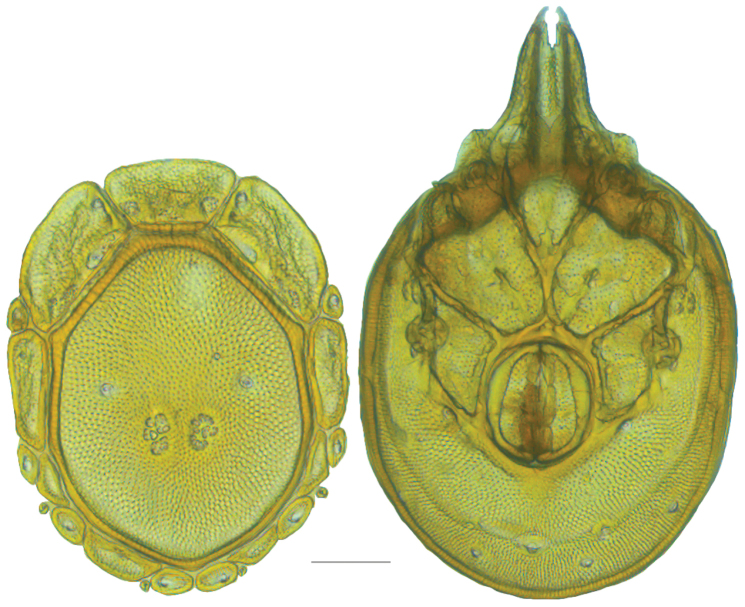
*Debsacarus oribatoides* male: (**Left**) dorsum; (**Right**) venter. Scale: 100 µm.

Dorsum (Fig. 11) — [534–590 long; 416–478 wide]. Dorsal plate [421–477 long; 332–380 wide] without secondary sclerotization. Dorso-glandularia-4 [157–188 apart] equally anterior to [25–55] and lateral to [31–53] muscle scars. Anterio-medial platelet [151–200 long; 90–108 wide]. Anterio-lateral platelet [169–186 long; 97–118 wide]. Lateral platelets as follows: lateral-1 [33–46 long; 22–31 wide]; lateral-2 [134–155 long; 48–55 wide]; lateral-3 [29–51 long; 17–24 wide]; lateral-4 [88–113 long; 31–40 wide]; lateral-5 [41–49 long; 22–35 wide]; lateral-6 [82–101 long; 27–42 wide]; lateral-7 [36–59 long; 20–38 wide].

Venter (Fig. 11) — [686–773 long; 449–515 wide]. Primary sclerotization [637–705 long]. Gnathosomal bay [21–36 dorsal length; 118–132 ventral length; 27–42 wide]. Coxal field [474–532 long; 307–332 wide]. Coxa-I [272–294 long; 143–169 midlength]. Coxa-II + III [123–138 distance to top of coxa-II; 192–208 distance to top of coxa-III; 377–417 distance to bottom of coxa-III; 253–279 total length]. Coxa-IV [327–368 length to top; 136–164 total length]. Genital field [388–435 distance to top; 536–598 distance to bottom; 148–165 total length; 126–150 width; 270–307 distance from gnathosomal bay; 108–144 distance from coxa-I; 88–107 distance to excretory pore; 143–179 distance to caudad]. Genital skeleton [210–265 long; 95–115 wide]. Distance to excretory pore [637–705].

Legs — total leg and podomere lengths as follows: Leg-I [447–476 total; trochanter 59–63; basifemur 80–88; telofemur 61–68; genu 79–88; tibia 85–95; tarsus 80–92]. Leg-II [479–526 total; trochanter 54–67; basifemur 82–89; telofemur 60–72; genu 80–90; tibia 94–105; tarsus 105–114]. Leg-III [544–624 total; trochanter 56–66; basifemur 79–102; telofemur 65–74; genu 95–110; tibia 119–138; tarsus 120–137]. Leg-IV [743–857 total; trochanter 85–110; basifemur 107–125; telofemur 113–130; genu 134–160; tibia 152–177; tarsus 145–158].

#### Diagnosis.

Same as genus.

#### Distribution.

Same as genus.

#### Remarks.

*Debsacarus oribatoides* show at least 15% COI divergence from all other Testudacarinae and less than .6% divergence from one another (Fig. 8). Additionally, Habeeb (1961) describes a protrusible maxillary tube, however, we find no evidence in the additional specimens examined that the maxillary tube or subcapitulum is any more protrusible than what is commonly found in other *Testudacarus*, and certainly is not protrusible like in *Pseudotorrenticola*. Habeeb (1961) did not designate types, however, he described the species from the only two specimens available. From those two specimens, we have designated a lectotype (♀) and paralectotype (♂).

### Testudacarus

Taxon classificationAnimaliaTrombidiformesTorrenticolidae

Walter, 1928

http://zoobank.org/F535321F-2CB2-4F9D-B955-659A39CC564D

Testudacarus Walter 1928: 75; Viets 1935: 601; Viets 1936: 143, 232; Lundblad 1941: 364; *Vitzthum 1942: 848; Marshall 1943: 318; Radford 1950: 120; Baker and Wharton 1952: 295; Pennak 1953: 479, 483–484; Bergstrom 1953: 157; Mitchell 1954: 40; Habeeb 1954: 14; Imamura 1955: 181; Viets 1956: 156, 255; Habeeb 1959a: 21; Newell 1959: 1086, 1099–1100; Habeeb 1961: 6; Lundblad 1967: 418; Conroy 1968: 29; Habeeb 1969: 2; Winger et al. 1972: 217; Barr 1972: 57–58, 67–68, 84, 86; Cook 1974: 145–146; Habeeb 1974a: 1; Habeeb 1974b: 1; Imamura 1976: 283; Barr 1977: 879; Williams et al. 1977: 2136; Pennak 1978: 497, 503; Fuste 1980: H7; Smith 1982: 901, 905, 922–923, 925–927, 929; Barr 1982: 155; Laubitz et al. 1983: 38; Viets 1987: 222, 724; Smith 1987: 51; Williams and Hogg 1988: 45; Bader 1988: 88, 90; Pennak 1989: 523, 528, 530; Peckarsky et al. 1990: 300, 320–321; Smith and Cook 1991: 552, 564, 574; Smith 1991a: 145, 151, 158; Smith 1991b: 811; Proctor 1992b: 238; Cramer 1992: 13–14; Wiles 1997a: 192–194, 197, 200, 202, 209; Wiles 1997b: 1243; Harvey 1998: 67; Smith and Cook 1999: 115; Cramer and Cook 2000: 51; Perrin 2001: 35, 56; Smith et al. 2001: 579, 592, 608, 645; Lewis and McCutchan 2005: 76; Guo and Jin 2005: 70; Abé 2005: 120; Abé 2006: 6; Perrin 2006: 24; Proctor 2006: 8, 13; Richards and Rogers 2006: 36; Pešić and Smit 2007: 50; Goldschmidt 2007: 444–445; GEI 2008: Appendix B-1, F-1, G-1; MMWD 2008: 13; Boyaci and Özkan 2008: 364; Hawkins 2009: 19; Stalingo 2009: 22; Walter et al. 2009: 264, 374; Herbst and Silldorff 2009: 70; Zhang and Guo 2010: 117; Smith 2010: 288; Smith et al. 2010: 492, 522, 535, 550; Herbst et al. 2010: 16; Pernot and Underwood 2010: 43, 46, 49, 52, 56, 59, 62, 65, 68; Pešić et al. 2010: 15; Perrin and Bennett 2011: 37; Guo and Zhang 2011: 46, 48–49; ME Inc. 2011 : 18; Richards and Rogers 2011: 45; Smith et al. 2011: 211; Herbst, Medhurst et al. 2011: 29; Herbst, Roberts et al. 2011: 23; Fernández and Reid 2012: 294–295, 297; Cuellar and Underwood 2012: 48, 54, 60, 66, 72; Morimoto 2012: 86; Herbst et al. 2013: 21; Fisher et al. 2015: 74, 83.
Testudacarus *Vitzthum (1942) is cited in Viets (1956), but this source was not located for this study.

#### Type species.

*Testudacarus tripeltatus* Walter, 1928

#### Generic diagnosis.

Members of this genus, unlike *Debsacarus*, lack projections on the anterior tips of coxae-I and have five-segmented pedipalps (instead of four). Furthermore, with the exception of *Testudacarus hyporhynchus*, they differ from *Debsacarus* in having a rounded gnathosoma (rather than elongate) and a wide gnathosomal bay that is uncovered dorsally and ventrally ends posterior to the leg-I insertion.

#### Distribution.

Same as subfamily.

### *Testudacarus minimus* complex

**Species complex diagnosis.**These species can be distinguished from most other testudacarines by their small size (female and male dorsal length less than 700 and 600 µm, respectively), highly variable coloration (red, orange, blue, violet, and rarely colorless), and small (<140 µm), rounded anterio-medial platelet (differing from *Testudacarus rollerae*, which has a large (>140 µm) anterio-medial platelet more than or nearly twice as wide as long). Additionally, only this complex and the *Testudacarus hitchensi* complex are present east of the Great Plains. These two complexes resemble each other morphologically in many respects, but can be easily distinguished because members of this complex have uniform coloration across all three anterior platelets while *Testudacarus hitchensi*-like mites have a colorless anterio-medial platelet and colored anterio-lateral platelets. With the exception of *Testudacarus radwellae*, males of this complex differ from *Testudacarus hitchensi*-like mites in having dorso-glandularia-4 positioned less anterior to and more lateral to the muscle scars. This complex is abundant and present across most of North America and comprises the following species: *Testudacarus deceptivus*, *Testudacarus minimus*, *Testudacarus radwellae*, and *Testudacarus vulgaris*.

**Figure 12.**
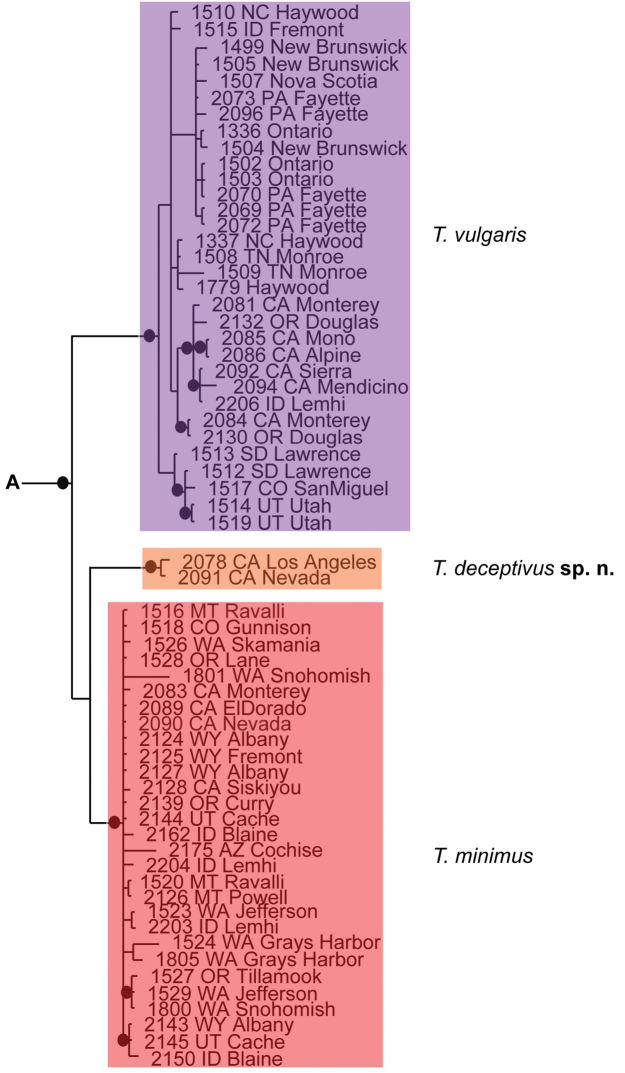
*Testudacarus minimus* complex molecular phylogeny: 28S and COI Bayesian analysis showing strong support for a soft polytomy with three distinct clades (●: >95% posterior probability); colored clades exhibit <2.5% divergence in COI within and >6.5% divergence between; continuation of (**A**) lineage from Fig. 6.

**Remarks.** Molecular data show strong support for three distinct clades (Fig. 12). All three clades exhibit less than 2.5% COI divergence within the clade and greater than 6.5% divergence between clades. In California there is currently no reliable way to diagnose these three clades morphologically as they are all roughly the same size and color (colorless to orange). However, outside of California it is possible to diagnose clades based on color, size, and geographic distribution. Members of this complex exhibit the broadest geographic ranges and thus exhibit the highest and not unexpected intraspecies divergence of the four complexes. Given the broad geographic sampling conducted in this complex, we feel comfortable designating the three main clades, exhibiting intra-clade divergence of more than 6.5%, as multiple species: *Testudacarus minimus*, *Testudacarus vulgaris*, and *Testudacarus deceptivus*. A fourth species, *Testudacarus radwellae*, belongs to this complex based on morphology, but genetic extractions were unsuccessful. *Testudacarus radwellae* males also share morphological similarities with *Testudacarus hitchensi*-like mites (the positioning of dorso-glandularia-4). Therefore *Testudacarus radwellae* is potentially important in discovering the relationship between these two species complexes and deserves further investigation.

#### Testudacarus minimus

Taxon classificationAnimaliaTrombidiformesTorrenticolidae

Marshall, 1943

http://zoobank.org/CD1D1B50-6A37-4099-86D7-A6DAB8A17CA6

Testudacarus minimus : Marshall 1943: 322; Bergstrom 1953: 159; Mitchell 1954: 40; Imamura 1955: 182, 188; Viets 1956: 255; Habeeb 1959a: 21; Crowell 1961: 329; Mitchell 1962: 42; Lundblad 1967: 418; Conroy 1968: 29; Habeeb 1974a: 1; Conroy and Scudder 1975: 307; Imamura 1976: 283; Viets 1987: 724-725; Smith et al. 2011: 262.
Testudacarus americanus : Habeeb 1967: 1.
Testudacarus americanus minimus : Habeeb 1969: 2.

##### Type series.

**Holotype (1♂): California, USA**: 1♂ from Santa Cruz County, Waddell Creek, 30–31 August 1933, by PR Needham, RM330016.

##### Other material examined.

**Other (15♀, 15♂): Montana, USA**: 2♂ from Ravalli County, Bitterroot National Forest, Lost Horse River, downstream of confluence of North Lost Horse (45°7'7.00"N, 114°18'0.00"W), 3 August 2012, by JR Fisher and WA Nelson, ROW12-0803-006; 1♂ from Powell County, Monture Creek, at fishing access off Highway 200 west of Ovando (47°2'15.00"N, 112°13'12.00"W), 9 August 2012, by AJ Radwell and JA Hinsey, AJR12-0809-415A; **Washington, USA**: 2♂ from Snohomish County, Mount Baker National Forest, Clean Creek, (48°13'8.00"N, 121°34'7.00"W), 28 July 2013, by JC O’Neill and WA Nelson, JNOW13-0728-007; 2♀ from Jefferson County, Olympic National Forest, Snow Creek, (47°56'11.00"N, 122°56'53.00"W), 22 July 2013, by WA Nelson and JC O’Neill, JNOW13-0722-001; 2♀ from Grays Harbor County, Capitol State Forest, Porter Creek, (46°58'13.00"N, 123°16'2.00"W), 25 July 2013, JC O’Neill and WA Nelson, JNOW13-0725-005; 1♀ from Skamania County, Gifford Pinchot National Forest, Lewis Creek, (46°7'40.00"N, 121°59'24.00"W), 1 August 2013, by JC O’Neill and WA Nelson, JNOW13-0801-004; **California, USA**: 1♂ from Inyo County, Inyo National Forest, Bishop Creek, downstream of campground (37°17'23.00"N, 118°33'14.00"W), 2 September 2013, by JR Fisher, JRF13-0902-003; 2♀ from Nevada County, Tahoe National Forest, Sagehen Creek, off Route 89 (39°26'2.00"N, 120°12'17.00"W), 26 August 2013, by JR Fisher, JRF13-0826-006; 1♀ from Siskiyou County, Klamath National Forest, Shadow Creek, off Cecilville Road, (41°12'13.00"N, 123°4'18.00"W), 17 August 2013, by JR Fisher, JRF13-0817-002; **Wyoming, USA**: 1♂ from Albany County, North Fork of Little Laramie River, at bridge on Highway 130 (41°19'42.00"N, 106°9'42.00"W), 3 August 2012, by AJ Radwell and JA Hinsey, AJR12-0803-406; 2♂ from Albany County, South Clear Creek, across from Southfork Campground on Highway 16 (44°16'36.00"N, 106°57'4.00"W), 14 August 2012, by AJ Radwell and JA Hinsey, AJR12-0814-419; 1♀ from Fremont County, Wind River, off County Road 773 30 miles east of Moran on Highway 26/287 (43°43'5.00"N, 110°48'0.00"W), 5 August 2012, by AJ Radwell and JA Hinsey, AJR12-0805-410; **Utah, USA**: 2♂ from Cache County, Wasatch-Cache National Forest, Jordan River, (41°44'33.00"N, 111°45'57.00"W), 24 July 2012, by JR Fisher and WA Nelson, ROW12-0724-004; **Idaho, USA**: 2♂ from Blaine County, Sawtooth National Forest, Baker Creek, (43°45'28.00"N, 114°33'44.00"W), 28 July 2012, by JR Fisher and WA Nelson, ROW12-0728-001; 2♂ from Lemhi County, Salmon National Forest, Niapas Creek at confluence with Panther Creek, (45°8'15.00"N, 114°13'4.00"W), 2 August 2012, by JR Fisher and WA Nelson, ROW12-0802-003; **Colorado, USA**: 1♀ from Gunnison County, Quartz Creek, north of Ohio City on County Road 76 mile marker 11 (38°34'2.00"N, 106°34'6.00"W), 1 August 2012, by AJ Radwell and JA Hinsey, AJR12-0801-403A; **Oregon, USA**: 1♀ from Tillamook County, Siuslaw National Forest, Alder Creek, (45°9'27.00"N, 123°47'60.00"W), 6 August 2013, by JC O’Neill, JNOW13-0806-002; 1♀ from Lane County, Gate Creek, (44°8'48.00"N, 122°34'20.00"W), 11 August 2013, by JC O’Neill and WA Nelson, JNOW 13-0811-001; 1♀ from Curry County, Rogue River National Forest, Elk River, off National Forest Road 5325 (42°42'46.00"N, 124°18'41.00"W), 13 August 2013, by JR Fisher, JRF13-0813-003; **Arizona, USA**: 1♀ from Cochise County, Chirichua Mountains west of Portal, East Turkey Creek, off Forest Road 42 above junction with Forest Road 42B (31°54'32.00"N, 109°15'11.00"W), 15 May 2011, by IM Smith, IMS110003; 1♀ from Cochise County, Chiricahua Mountains west of Portal, East Turkey Creek, off Forest Road 42 just above junction with Forest Road 42B (31°54'32.00"N, 109°15'11.00"W), 15 May 2011, by IM Smith, IMS110004.

##### Type deposition.

Holotype (1♂) deposited at the CNC.

##### Diagnosis.

*Testudacarus minimus* most resemble *Testudacarus vulgaris* and *Testudacarus deceptivus*. Throughout the majority of their shared range in the west, *Testudacarus minimus* are orange to red and *Testudacarus vulgaris* are violet to blue. While these two species have overlapping size ranges, *Testudacarus minimus* are generally larger. *Testudacarus vulgaris* females rarely exhibit a dorsal length over 600 µm and males rarely exceed 500 µm while *Testudacarus minimus* females and males are usually larger than 600 and 500 µm, respectively. *Testudacarus deceptivus* have only been found in two counties in California and cannot be distinguished from either *Testudacarus minimus* or *Testudacarus vulgaris* using morphology. *Testudacarus minimus* are the only members of their complex that have been found in Washington and northern Oregon.

##### Redescription.

**Female (n=14)** with characteristics of the genus with following specifications.

Gnathosoma — Subcapitulum [154–173 ventral length; 96–108 dorsal length; 90–105 tall] elliptic to ovoid with short rostrum. Chelicerae [133–152 long] unmodified with slightly curved fangs [29–32 long]. Pedipalp [181–202 long] unmodified. Trochanter [25–30 long; 30–35 wide]. Femur [49–58 long; 38–42 wide]. Genu [38–42 long; 32–35 wide]. Tibia [45–52 long; 22–25 wide]. Tarsus [19–23 long; 9–12 wide].

**Figure 13.**
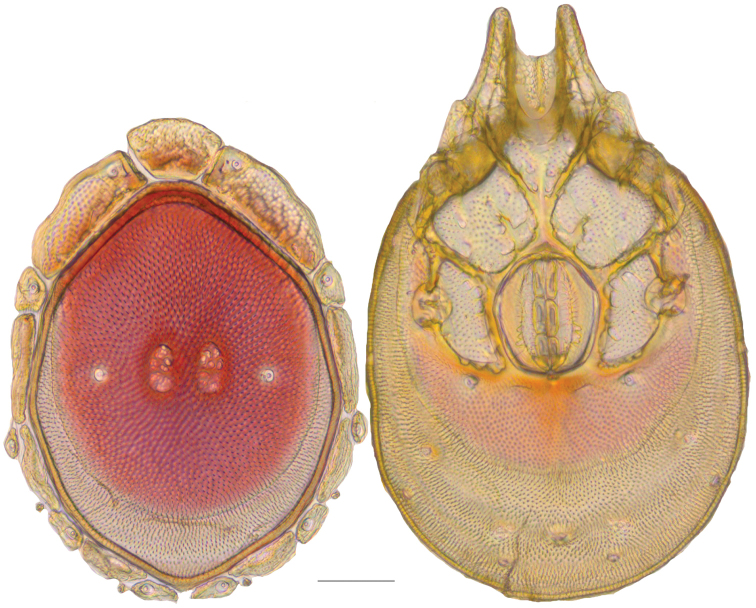
*Testudacarus minimus* female: (**Left**) dorsum; (**Right**) venter. Scale: 100 µm.

**Figure 14.**
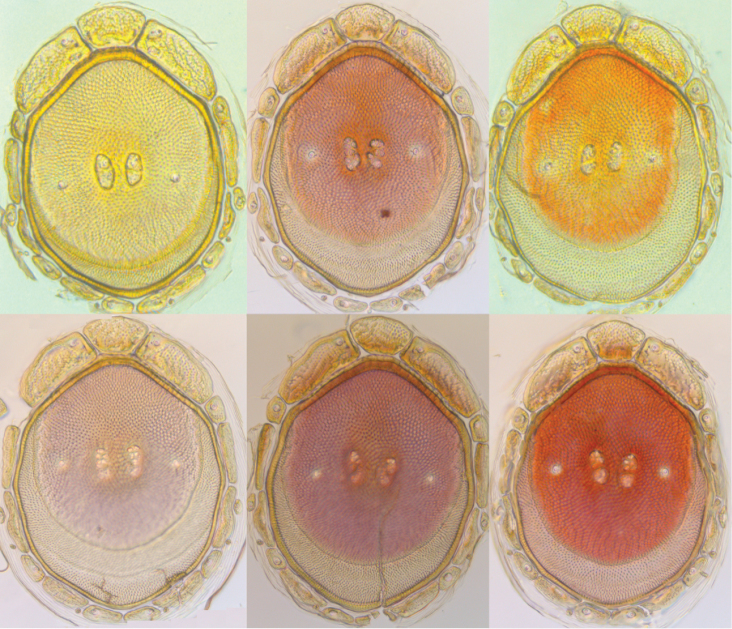
*Testudacarus minimus* color variation.

Dorsum (Fig. 13) — [571–699 long; 442–533 wide] round to ovoid. Dorsal plate [464–591 long; 375–457 wide]. Primary sclerotization [405–467 long] color variable (Fig. 14). Dorso-glandularia-4 [190–250 apart] in line with and lateral to [51-71] muscle scars. Platelets mostly colorless but with hints of primary sclerotization color. All three anterior platelets with color either completely absent or present proximally but restricted distally. Anterio-medial platelet [115–139 long; 73–86 wide] rounded trapezoid noticeably smaller than anterio-lateral platelets [161–190 long; 65–86 wide]. Lateral platelets as follows: lateral-1 [42–63 long; 28–43 wide]; lateral-2 [120–148 long; 24–36 wide]; lateral-3 [32–46 long; 16–24 wide]; lateral-4 [91–138 long; 22–32 wide]; lateral-5 [41–68 long; 21–37 wide]; lateral-6 [76–117 long; 19–41 wide]; lateral-7 [49–78 long; 19–34 wide].

Venter (Fig. 13) — [731–865 long; 466–556 wide] round to ovoid. Primary sclerotization [566–658 long] usually with dorsal plate color or colorless. Gnathosomal bay [54–82 dorsal length; 122–158 ventral length; 49–65 wide]. Coxal field [434–495 long; 303–366 wide]. Coxa-I [231–261 long; 94–111 midlength]. Coxa-II + III [105–127 distance to top of coxa-II; 171–201 distance to top of coxa-III; 312–362 distance to bottom of coxa-III; 201–242 total length]. Coxa-IV [434–495 distance to top; 132–155 total length]. Genital field [288–340 distance to top; 450–512 distance to bottom; 142–183 total length; 124–150 width; 164–184 distance from gnathosomal bay; 57–81 distance from coxa-I; 182–226 distance to excretory pore; 276–353 distance to caudad]. Eggs [130–135 long; 1–4 eggs]. Distance to excretory pore [637–737].

Legs — colorless, or with same color as dorsal plate. Total leg and podomere lengths as follows: Leg-I [428–477 total; trochanter 48–55; basifemur 72–85; telofemur 60–69; genu 78–90; tibia 83–95; tarsus 79–92]. Leg-II [453–530 total; trochanter 54–62; basifemur 74–87; telofemur 58–68; genu 83–96; tibia 96–110; tarsus 99–113]. Leg-III [440–625 total; trochanter 55–65; basifemur 76–88; telofemur 64–76; genu 106–117; tibia 120–137; tarsus 131–148]. Leg-IV [677–843 total; trochanter 87–97; basifemur 106–120; telofemur 111–122; genu 146–160; tibia 160–173; tarsus 147–180].

**Male (n=16)** similar to female except for sexually dimorphic characters previously discussed and with following specifications.

Gnathosoma — Subcapitulum [138–164 ventral length; 88–105 dorsal length; 83–93 tall]. Chelicerae [120–145 long]. Fangs [27–30 long]. Pedipalp [181–206 long]. Trochanter [24–32 long; 28–33 wide]. Femur [48–59 long; 35–40 wide]. Genu [38–46 long; 29–34 wide]. Tibia [43–54 long; 19–25 wide]. Tarsus [16–22 long; 9–12 wide].

**Figure 15.**
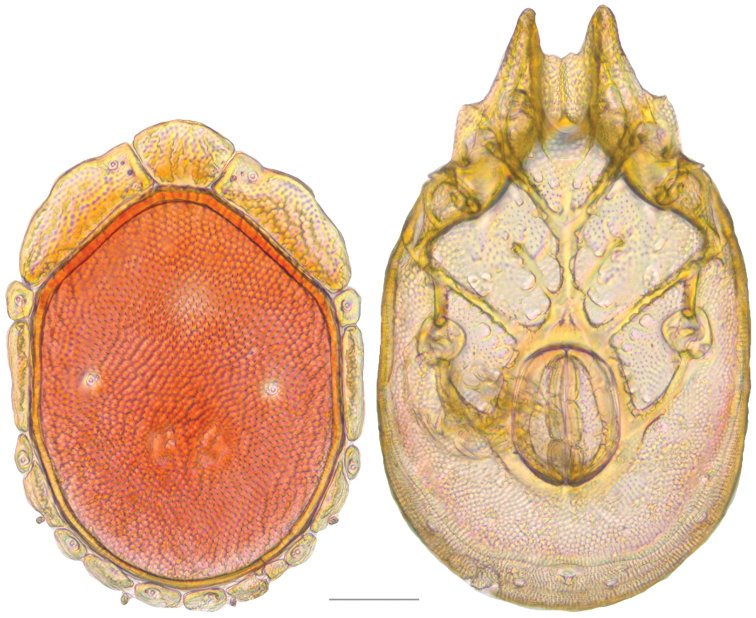
*Testudacarus minimus* male: (**Left**) dorsum; (**Right**) venter. Scale: 100 µm.

Dorsum (Fig. 15) — [486–549 long; 356–417 wide]. Dorsal plate [406–470 long; 315–372 wide]. Dorso-glandularia-4 [141–219 apart] slightly anterior to [15-52] and well lateral to [31–64] muscle scars. Anterio-medial platelet [99–129 long; 63–80 wide]. Anterio-lateral platelets [151–179 long; 59–76 wide]. Lateral platelets as follows: lateral-1 [31–46 long; 23–32 wide]; lateral-2 [99–124 long; 20–28 wide]; lateral-3 [34–48 long; 14–23 wide]; lateral-4 [65–97 long; 17–28 wide]; lateral-5 [39–56 long; 15–27 wide]; lateral-6 [51–69 long; 17–28 wide]; lateral-7 [42–56 long; 18–28 wide].

Venter (Fig. 15) — [596–717 long; 379–457 wide]. Primary sclerotization [564–650 long]. Gnathosomal bay [53–68 dorsal length; 120–150 ventral length; 51–63 wide]. Coxal field [412–480 long; 290–329 wide]. Coxa-I [215–249 long; 83–105 midlength]. Coxa-II + III [95–115 distance to top of coxa-II; 158–191 distance to top of coxa-III; 329–380 distance to bottom of coxa-III; 230–265 total length]. Coxa-IV [293–328 length to top; 119–153 total length]. Genital field [357–406 distance to top; 493–569 distance to bottom; 129–164 total length; 114–127 width; 228–258 distance from gnathosomal bay; 128–160 distance from coxa-I; 63–91 distance to excretory pore; 101–154 distance to caudad]. Genital skeleton [190–215 long; 93–109 wide]. Distance to excretory pore [564–650].

Legs — total leg and podomere lengths as follows: Leg-I [435–483 total; trochanter 53–63; basifemur 75–84; telofemur 57–69; genu 78–89; tibia 82–93; tarsus 80–90]. Leg-II [458–518 total; trochanter 52–64; basifemur 75–87; telofemur 59–69; genu 79–90; tibia 92–104; tarsus 96–109]. Leg-III [530–599 total; trochanter 54–62; basifemur 75–88; telofemur 63–72; genu 97–111; tibia 114–133; tarsus 124–137]. Leg-IV [722–813 total; trochanter 81–95; basifemur 102–122; telofemur 103–118; genu 130–159; tibia 150–167; tarsus 145–158].

##### Distribution.

Abundant throughout North America, ranging from the Pacific Northwest to the southwestern United States (and potentially into northern Mexico), and east into the western Great Plains.

##### Remarks.

Commonly colorless or orange in the southwestern United States; red, pink, or orange–red in the northwest, Rocky Mountains, and western Great Plains; and uncommonly red–violent in the northwest, Rocky Mountains, and western Great Plains.

#### Testudacarus vulgaris

Taxon classificationAnimaliaTrombidiformesTorrenticolidae

Habeeb, 1954

http://zoobank.org/AD09023D-849F-4F13-BD0C-1CF7B6623748

Testudacarus vulgaris : Habeeb 1954: 14; Habeeb 1956: 2; Viets 1956: 256; Habeeb 1959a: 21; Crowell 1961: 329; Lundblad 1967: 418; Habeeb 1967: 4; Imamura 1976: 283; Smith 1987: 51; Viets 1987: 724–725 Smith 2010: 295, 302, 305.
Testudacarus american vulgaris : Habeeb 1969: 1, 2; Viets 1987: 724–725.
Testudacarus minimus vulgaris : Habeeb 1974a: 1; Viets 1987: 724–725.

##### Type series.

**Syntypes (1**♀, **1**♂): **New Brunswick, Canada**: from Victoria County, Salmon River, 21 June 1953, by H. Habeeb, 87-53

##### Other material examined.

**Other (18♀, 19♂): Ontario, Canada**: 1♀ and 1♂ from Lennox and Addington County, Hydes Creek, beside Highway 41 23.7km north of Highway 28 at Denbigh (45°11'22.00"N, 77°13'38.00"W), 29 April 2010, by IM Smith, IMS100023; 1♀ from Hastings County, Maple Leaf and Papineau Creek, east of Davis Road before Highway 62, 18 August 2011, by IM Smith, IMS110053; **New Brunswick, Canada**: 2♀ and 1♂ from Victoria County, Little Wapske River, Plaster Rock beside Highway108 20.5km east of Highway109, 5 September 2011, by IM Smith, IMS110061; **Nova Scotia, Canada**: 1♂ from Inervess County, Cheticamp River, 10 September 2011, by IM Smith, IMS110071; **Tennessee, USA**: 1♀ and 1♂ from Monroe County, Turkey Creek, beside Forest Road #210 just east of Forest Road #35 7.1km southeast of Route 165 (35°20'28.00"N, 84°11'30.00"W), 12 September 2009, by IM Smith, IMS090110; 2♂ from Sevier County, Great Smoky Mountains Nation Park, Rhododendron Creek, beside Greenbrier Road 2.2 km south of Route 321 (35°43'32.00"N, 83°24'2.00"W), 2 September 2009, by IM Smith, IMS090093; **North Carolina, USA**: 2♀ and 1♂ from Haywood County, Great Smoky Mountains National Park, Big Creek, Waterville Big Creek Picnic Area (35°44'59.00"N, 83°6'42.00"W), 16 September 2010, by IM Smith, IMS100138; 1♀ and 1♂ from Haywood County, Great Smoky Mountains National Park, Cataloochee Creek, beside Mount Sterling Road near bridge 1.7km north of road to campground (35°38'45.00"N, 83°4'34.00"W), 6 September 2009, by IM Smith, IMS090099; 1♀ from Haywood County, Great Smoky Mountains National Park, Cataloochee Creek, beside Mount Sterling Road near bridge 1.7km north of road to campground (35°38'45.00"N, 83°4'32.00"W), 20 September 2010, by IM Smith, IMS100150; **South Dakota, USA**: 1♀ and 1♂ from Lawrence County, Jim Creek, south of Nemo Road on Goodhope Road behind cab at Green Mountain Black Hills (44°9'9.00"N, 103°28'51.00"W), 15 August 2012, by AJ Radwell and JA Hinsey, AJR12-0815-421; **Colorado, USA**: 1♂ from San Miguel County, San Miguel River, beside Route 145 12.5km northwest of junction with road to Telluride (37°59'17.00"N, 107°59'34.00"W), 31 July 2012, by AJ Radwell and JA Hinsey, AJR12-0731-400; Pennsylvania, USA: 1♂ from Fayette County, Ohiophyle State Park, Laurel Run, fishing access #2 off T798 (Meadow Run Road) (39°50'58.00"N, 79°30'51.00"W), 10 August 2014, by MJ Skvarla, MS14-0810-005; 2♀ and 2♂ from Fayette County, State Game Lands #51, Dunbar Creek, off Furnace Hill Road East of Dunbar (39°56'16.10"N, 79°35'3.70"W), 10 August 2014, by MJ Skvarla, MS14-0810-002; **California, USA**: 1♂ from Monterey County, Andrew Molera State Park, Big Sur River, off Route 1 (36°16'31.00"N, 121°49'14.00"W), 4 September 2013, by JR Fisher, JRF13-0904-003; 1♂ from Inyo County, Inyo National Forest, Bishop Creek, downstream of campground (37°17'23.00"N, 118°33'14.00"W), 2 September 2013, by JR Fisher, JRF13-0902-003; 1♂ from Alpine County, Markleeville Creek, off Route 89 downstream of bridge (38°41'39.00"N, 119°46'41.00"W), 30 August 2013, by JR Fisher, JRF13-0830-001; 1♂ from Mendocino County, Jackson Demonstration State Park, North Fork of Big River, (39°20'46.00"N, 123°30'35.00"W), 22 August 2013, by JR Fisher, JRF13-0822-002; 1♀ from Mono County, Humboldt-Toiyabe National Forest, Little Walker River, off Route 108 downstream of tunnel (38°20'57.00"N, 119°27'15.00"W), 31 August 2013, by JR Fisher, JRF13-0831-002; 1♀ from Trinity County, Shasta-Trinity National Forest, North Fork of Trinity River, (40°46'47.00"N, 123°7'46.00"W), 18 August 2013, JRF13-0818-005; **Oregon, USA**: 2♂ from Douglas County, Umpqua National Forest, Calf Creek, (43°17'28.00"N, 122°37'12.00"W), 12 August 2013, by JC O’Neill and WA Nelson, JNOW13-0812-006; **Utah, USA**: 2♀ from Utah County, Uinta National Forest, Hobble Creek, just upstream on right fork Hobble Creek Road from Cherry Campground (40°10'9.00"N, 111°28'26.00"W), 22 July 2012, by JR Fisher and WA Nelson, ROW12-0722-001; **Idaho, USA**: 1♀ from Fremont County, Targhee National Forest, Rock Creek, downstream of tributary (44°6'44.00"N, 111°15'4.00"W), 25 July 2012, by JR Fisher and WA Nelson, ROW12-0725-001; **Arkansas, USA**: 1♀ from Searcy County, Tomahawk Creek, (36°1'20.00"N, 92°40'43.00"W), 20 July 2009, by AJ Radwell, AJR090101.

##### Type deposition.

Syntypes (1♀, 1♂) deposited at the CNC.

##### Diagnosis.

*Testudacarus vulgaris* most resemble *Testudacarus minimus* and *Testudacarus deceptivus*. Throughout the majority of their shared range in the west, *Testudacarus minimus* are orange to red and *Testudacarus vulgaris* are violet to blue. While these two species have overlapping size ranges, *Testudacarus minimus* are generally larger. *Testudacarus vulgaris* females rarely exhibit a dorsal length over 600 µm and males rarely exceed 500 µm while *Testudacarus minimus* females and males are usually larger than 600 and 500 µm, respectively. *Testudacarus deceptivus* have only been found in two counties in California and cannot be distinguished from either *Testudacarus minimus* or *Testudacarus vulgaris* using morphology. *Testudacarus vulgaris* are the only members of their complex that have been found east of the Great Plains.

##### Redescription.

**Female (n=18)** with characteristics of the genus with following specifications.

Gnathosoma — Subcapitulum [151–190 ventral length; 90–114 dorsal length; 84–115 tall] elliptical to ovoid with short rostrum. Chelicerae [133–170 long] unmodified with lightly curved fangs [28–35 long]. Pedipalp [169–211 long] unmodified. Trochanter [23–32 long; 28–37 wide]. Femur [46–62 long; 33–45 wide]. Genu [33–42 long; 28–36 wide]. Tibia [42–53 long; 19–26 wide]. Tarsus [18–23 long; 9–12 wide].

**Figure 16.**
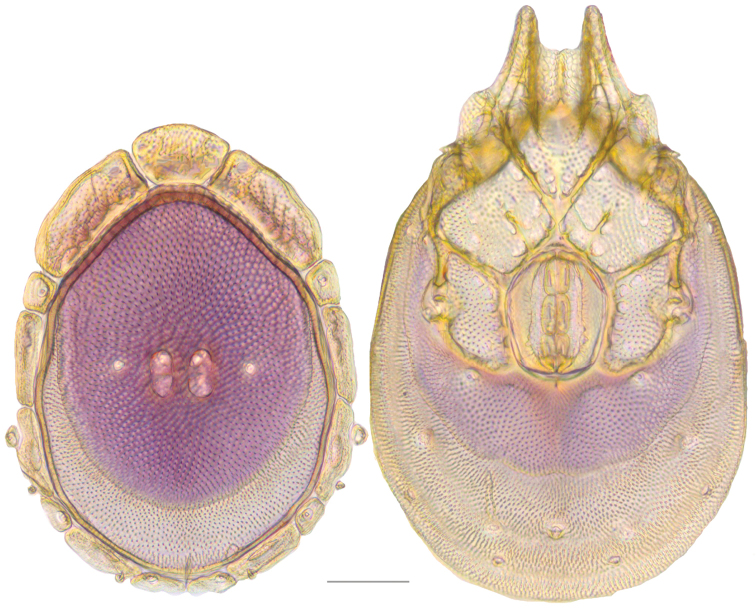
*Testudacarus vulgaris* female: (**Left**) dorsum; (**Right**) venter. Scale: 100 µm.

**Figure 17.**
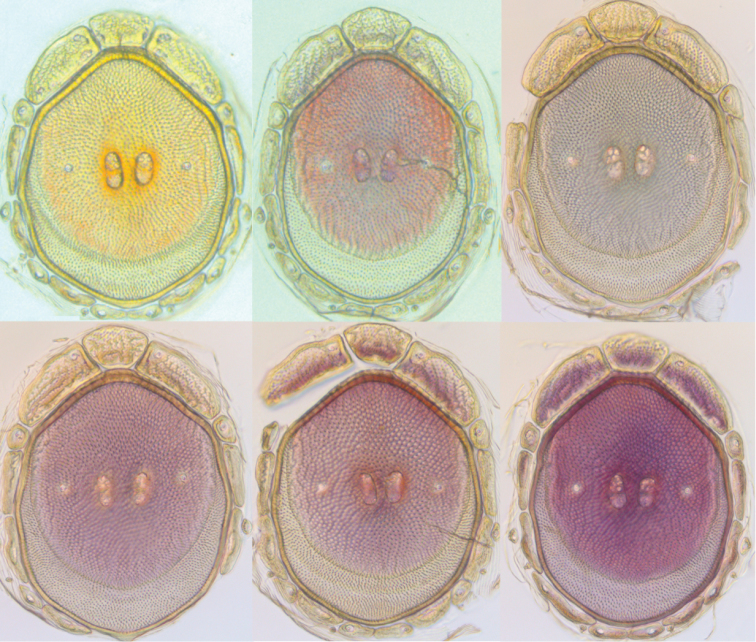
*Testudacarus vulgaris* color variation.

Dorsum (Fig. 16) — [547–654 long; 394–517 wide] round to ovoid. Dorsal plate [391–582 long; 330–470 wide]. Primary sclerotization [357–500 long] color variable (Fig. 17). Dorso-glandularia-4 [143–247 apart] in line with and lateral to [39–65] muscle scars. Platelets mostly colorless but with hints of primary sclerotization color. All three anterior platelets with color either completely absent or present proximally but restricted distally. Anterio-medial platelet [111–142 long; 67–94 wide] rounded trapezoid noticeably smaller than anterio-lateral platelets. Anterio-lateral platelets [152–203 long; 68–88 wide]. Lateral platelets as follows: lateral-1 [39–72 long; 29–44 wide]; lateral-2 [108–141 long; 25–35 wide]; lateral-3 [16–60 long; 15–22 wide]; lateral-4 [99–136 long; 21–36 wide]; lateral-5 [43–72 long; 20–29 wide]; lateral-6 [77–109 long; 15–38 wide]; lateral-7 [59–73 long; 20–31 wide].

Venter (Fig. 16) — [670–835 long; 436–557 wide] round to ovoid. Primary sclerotization [522–686 long] with dorsal plate color or colorless. Gnathosomal bay [53–80 dorsal length; 118–169 ventral length; 51–70 wide]. Coxal field [404–500 long; 289–398 wide]. Coxa-I [213–273 long; 82–115 midlength]. Coxa-II + III [97–125 distance to top of coxa-II; 157–192 distance to top of coxa-III; 299–371 distance to bottom of coxa-III; 196–257 total length]. Coxa-IV [285–339 distance to top; 110–161 total length]. Genital field [275–348 distance to top; 421–516 distance to bottom; 141–171 total length; 105–143 width; 148–187 distance from gnathosomal bay; 50–81 distance from coxa-I; 140–234 distance to excretory pore; 231–340 distance to caudad]. Eggs [130–150 long; 1–4 eggs]. Distance to excretory pore [582–750].

Legs — colorless, or with same color as dorsal plate. Total leg and podomere lengths as follows: Leg-I [401–497 total; trochanter 50–61; basifemur 74–85; telofemur 55–72; genu 72–96; tibia 75–97; tarsus 78–97]. Leg-II [417–564 total; trochanter 51–63; basifemur 71–92; telofemur 57–72; genu 75–100; tibia 92–118; tarsus 96–120]. Leg-III [513–664 total; trochanter 55–68; basifemur 71–96; telofemur 58–82; genu 91–124; tibia 112–147; tarsus 124–157]. Leg-IV [726–911 total; trochanter 85–105; basifemur 103–132; telofemur 99–138; genu 134–174; tibia 145–177; tarsus 148–185].

**Male (n=17)** similar to female except for sexually dimorphic characters previously discussed and with following specifications.

Gnathosoma — Subcapitulum [128–155 ventral length; 83–96 dorsal length; 78–95 tall]. Chelicerae [115–145 long]. Fangs [25–29 long]. Pedipalp [156–190 long]. Trochanter [22–28 long; 28–33 wide]. Femur [42–55 long; 32–42 wide]. Genu [32–41 long; width 25–32 wide]. Tibia [43–52 long; 19–23 wide]. Tarsus [16–21 long; 9–11 wide].

**Figure 18.**
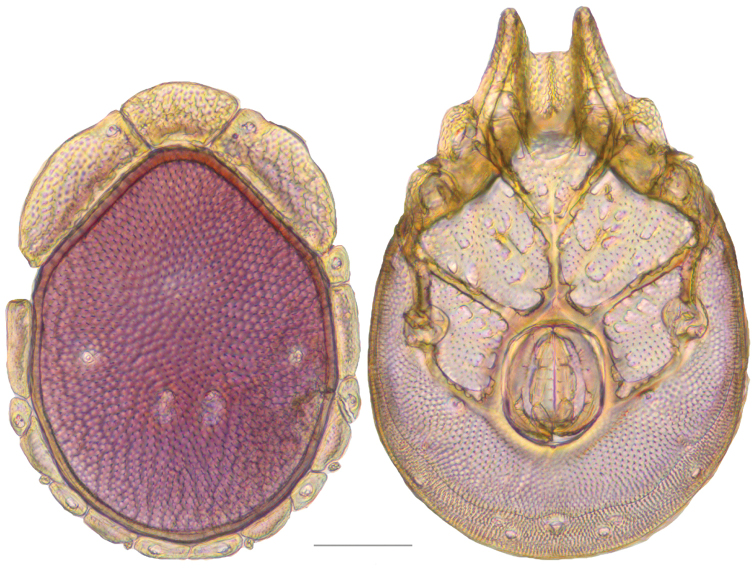
*Testudacarus vulgaris* male: (**Left**) dorsum; (**Right**) venter. Scale: 100 µm.

Dorsum (Fig. 18) — [439–525 long; 314–390 wide]. Dorsal plate [359–438 long; 283–342 wide]. Dorso-glandularia-4 [140–205 apart] anterior to [15–51] and well lateral to [33–70] muscle scars. Anterio-medial platelet [100–125 long; 64–76 wide]. Anterio-lateral platelet [142–175 long; 57–74 wide]. Lateral platelets as follows: lateral-1 [33–49 long; 20–34 wide]; lateral-2 [86–117 long; 20–28 wide]; lateral-3 [30–44 long; 13–23 wide]; lateral-4 [58–92 long; 16–28 wide]; lateral-5 [37–52 long; 18–24 wide]; lateral-6 [43–73 long; 16–26 wide]; lateral-7 [43–57 long; 14–25 wide].

Venter (Fig. 18) — [534–676 long; 341–427 wide]. Primary sclerotization [491–631 long]. Gnathosomal bay [42–68 dorsal length; 116–150 ventral length; 50–60 wide]. Coxal field [365–460 long; 265–321 wide] Coxa-I [195–251 long; 73–104 midlength]. Coxa-II + III [85–106 distance to top of coxa-II; 139–176 distance to top of coxa-III; 296–377 distance to bottom of coxa-III; 208–276 total length]. Coxa-IV [249–310 length to top; 113–150 total length]. Genital field [311–399 distance to top; 434–544 distance to bottom; 123–147 total length; 98–118 width; 195–251 distance from gnathosomal bay; 106–147 distance from coxa-I; 48–95 distance to excretory pore; 98–132 distance to caudad]. Genital skeleton [153–193 long; 80–94 wide]. Distance to excretory pore [491–631].

Legs — total leg and podomere lengths as follows: Leg-I [402–452 total; trochanter 49–59; basifemur 67–80; telofemur 53–63; genu 70–82; tibia 75–88; tarsus 78–88]. Leg-II [421–488 total; trochanter 51–61; basifemur 68–81; telofemur 51–63; genu 73–86; tibia 84–96; tarsus 91–105]. Leg-III [501–552 total; trochanter 52–61; basifemur 72–82; telofemur 59–68; genu 89–100; tibia 105–119; tarsus 118–130]. Leg-IV [664–746 total; trochanter 79–90; basifemur 95–106; telofemur 92–108; genu 124–144; tibia 130–155; tarsus 129–150].

##### Distribution.

Abundant throughout the majority of North America. Unreported in Washington and northern Oregon.

##### Remarks.

Commonly orange and uncommonly violet in the southwestern United States; commonly violet or blue and uncommonly red–violet in the Rocky Mountains and Great Plains; commonly violet or blue east of the Great Plains.

#### Testudacarus deceptivus

Taxon classificationAnimaliaTrombidiformesTorrenticolidae

O’Neill & Dowling sp. n.

http://zoobank.org/13FDE612-2F95-4498-939E-E95CAD6403CD

##### Type series.

**Holotype (1♀): California, USA**: 1♀ from Los Angeles County, Angeles National Forest, North Fork of San Gabriel River, off Route 39 (34°16'16.00"N, 117°50'46.00"W), 8 September 2013, by JR Fisher, JRF13-0908-001 (Specimen 143652 – DNA#2078); **Paratype (1♂): California, USA**: (allotype) 1♂ from Sierra County, Tahoe National Forest, Milton Creek near confluence of North Yuba River, (39°34'4.00"N, 120°36'54.00"W), 25 August 2013, by JR Fisher, JRF13-0825-004 (Specimen 143666 – DNA#2091)

##### Type deposition.

Holotype (1♀) and allotype (1♂) deposited at the CNC.

##### Diagnosis.

*Testudacarus deceptivus* have only been found in two counties (Los Angeles and Sierra) in California and cannot be distinguished from either *Testudacarus minimus* or *Testudacarus vulgaris* using morphology.

**Description. Female (n=1)** with characteristics of the genus with following specifications.

Gnathosoma — Subcapitulum [174 ventral length; 104 dorsal length; 90 tall] elliptical to ovoid with short rostrum and colorless. Chelicerae [144 long] unmodified with lightly curved fangs [32 long]. Pedipalp [190 long] unmodified. Trochanter [28 long; 29 wide]. Femur [53 long; 42 wide]. Genu [39 long; 32 wide]. Tibia [50 long; 23 wide]. Tarsus [19 long; 10 wide].

**Figure 19.**
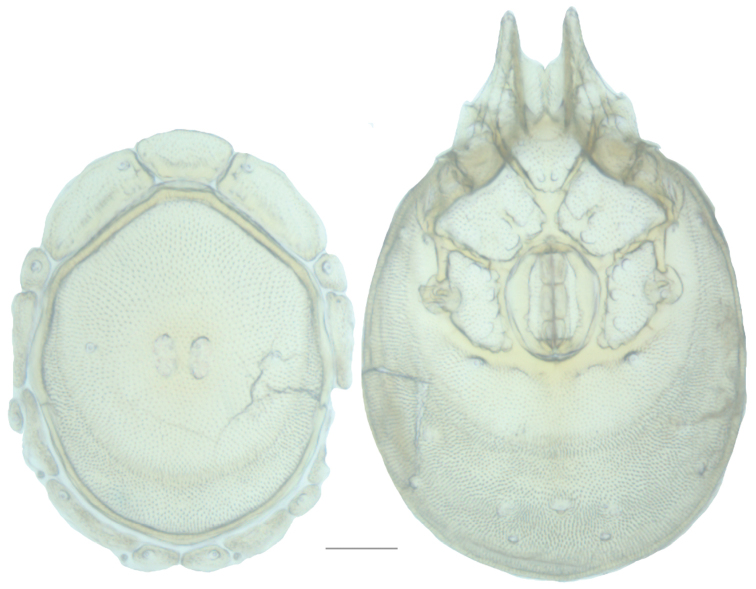
*Testudacarus deceptivus* female: (**Left**) dorsum; (**Right**) venter. Scale: 100 µm.

Dorsum (Fig. 19) — [597 long; 468 wide] ovoid and colorless. Dorsal plate [500 long; 410 wide]. Primary sclerotization [420 long]. Dorso-glandularia-4 [244 apart] in line with and well lateral to [78] muscle scars. Platelets completely colorless. Anterio-medial platlet [133 long; 74 wide]. Anterio-lateral platelet [168 long; 70 wide]. Lateral platelets as follows: lateral-1 [54 long; 43 wide]; lateral-2 [126 long; 31 wide]; lateral-3 [42 long; 20 wide]; lateral-4 [115 long; 29 wide]; lateral-5 [45 long; 27 wide]; lateral-6 [89 long; 30 wide]; lateral-7 [62 long; 27 wide].

Venter (Fig. 19) — [777; 521 wide] ovoid and colorless. Primary sclerotization [600 long]. Gnathosomal bay [76 dorsal length; 145 ventral length; 60 wide]. Coxal field [458 long; 336 wide]. Coxa-I [248 long; 102 midlength]. Coxa-II + III [117 distance to top of coxa-II; 192 distance to top of coxa-III; 340 distance to bottom of coxa-III; 223 total length]. Coxa-IV [322 distance to top; 136 total length]. Genital field [318 distance to top; 479 distance to bottom; 160 total length; 133 width; 173 distance from gnathosomal bay; 70 distance from coxa-I; 188 distance to excretory pore; 299 distance to caudad]. Distance to excretory pore [666].

Legs — colorless. Total leg and podomere lengths as follows: Leg-I [480 total; trochanter 62; basifemur 80; telofemur 64; genu 91; tibia 92; tarsus 90]. Leg-II [519 total; trochanter 63; basifemur 83; telofemur 69; genu 94; tibia 104; tarsus 106]. Leg-III [615 total; trochanter 63; basifemur 85; telofemur 72; genu 115; tibia 133; tarsus 145]. Leg-IV [821 total; trochanter 93; basifemur 112; telofemur 122; genu 161; tibia 178; tarsus 155].

**Male (n=1)** similar to female except for sexually dimorphic characters previously discussed and with following specifications.

Gnathosoma — Subcapitulum [139 ventral length; 90 dorsal length; 83 tall]. Chelicerae [125 long]. Fangs [29 long]. Pedipalp [179 long]. Trochanter [26 long; 29 wide]. Femur [48 long; 35 wide]. Genu [40 long; width 29 wide]. Tibia [44 long; 23 wide]. Tarsus [20 long; 10 wide].

**Figure 20.**
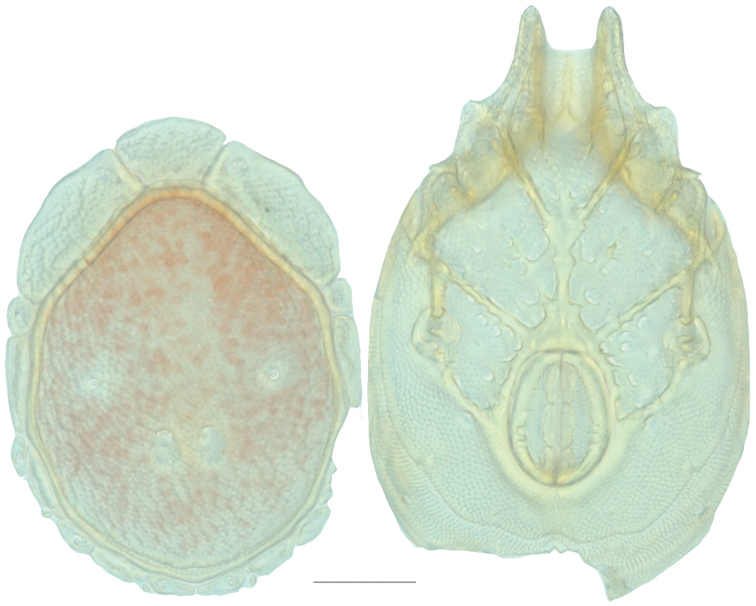
*Testudacarus deceptivus* male: (**Left**) dorsum; (**Right**) venter. Scale: 100 µm.

Dorsum (Fig. 20) — [470 long; 350 wide]. Dorsal plate [397 long; 317 wide]. Dorso-glandularia-4 [169 apart] anterior [39] and lateral to [47] muscle scars. Anterio-medial platelet [105 long; 67 wide]. Anterio-lateral platelets [154 long; 62 wide]. Lateral platelets as follows: lateral-1 [36 long; 29 wide]; lateral-2 [90 long; 20 wide]; lateral-3 [36 long; 14 wide]; lateral-4 [70 long; 20 wide]; lateral-5 [39 long; 15 wide]; lateral-6 [59 long; 16 wide]; lateral-7 [44 long; 16 wide].

Venter (Fig. 20) — [600; 386 wide]. Primary sclerotization [554 long]. Gnathosomal bay [54 dorsal length; 131 ventral length; 52 wide]. Coxal field [413 long; 290 wide]. Coxa-I [219 long; 88 midlength]. Coxa-II + III [96 distance to top of coxa-II; 168 distance to top of coxa-III; 331 distance to bottom of coxa-III; 235 total length]. Coxa-IV [291 length to top; 122 total length]. Genital field [354 distance to top; 491 distance to bottom; 137 total length; 107 width; 223 distance from gnathosomal bay; 135 distance from coxa-I; 63 distance to excretory pore; 91 distance to caudad]. Genital skeleton [192 long; 89 wide]. Distance to excretory pore [554].

Legs — total leg and podomere lengths as follows: Leg-I [413 total; trochanter 51; basifemur 69; telofemur 61; genu 73; tibia 79; tarsus 78]. Leg-II [462 total; trochanter 60; basifemur 75; telofemur 59; genu 80; tibia 94; tarsus 93]. Leg-III [517 total; trochanter 56; basifemur 73; telofemur 65; genu 95; tibia 111; tarsus 116]. Leg-IV [688 total; trochanter 76; basifemur 97; telofemur 97; genu 132; tibia 146; tarsus 138].

##### Etymology.

Specific epithet *deceptivus* (*decept*-, L. deceptive) refers to the lack of morphological characters differentiating this species from related species.

##### Distribution.

Known from only two counties (Los Angeles and Sierra) in California.

#### Testudacarus radwellae

Taxon classificationAnimaliaTrombidiformesTorrenticolidae

O’Neill & Dowling sp. n.

http://zoobank.org/D9D64AA5-FBE6-4156-BB22-FC4A0B834D96

##### Type series.

**Holotype (1♀): Arkansas, USA**: 1♀ from Montgomery County, Ouachita National Forest, Collier Springs, at spring structure picnic area (34°29'7.04"N, 93°35'38.12"W), 11 November 2009, by AJ Radwell, AJR090317C (Specimen 144016); **Paratypes (1♀, 7♂): Arkansas, USA**: (allotype) 1♂ from Montgomery County, Ouachita National Forest, Collier Springs, at spring structure picnic area (34°29'7.04"N, 93°35'38.12"W), 29 July 2011, by AJ Radwell and B Crump, AJR110301 (Specimen 144011); 4♂ from Montgomery County, Ouachita National Forest, Collier Springs, at spring structure picnic area (34°29'7.04"N, 93°35'38.12"W), 29 July 2011, by AJ Radwell and B Crump, AJR110301; 1♂ from Polk County, Ouachita National Forest, upper small pond on stream running along trail (34°27'36.73"N, 93°59'52.38"W), 21 July 2008, by AJ Radwell, AJR080303A; 1♂ from Montgomery County, Ouachita National Forest, Collier Springs, picnic area beside Forest Road 177 (34°29'3.00"N, 93°35'35.00"W), 19 September 2008, by IM Smith, IMS080061A; 1♀ from Montgomery County, Ouachita National Forest, Collier Springs, at spring structure picnic area (34°29'7.04"N, 93°35'38.12"W), 11 November 2009, by AJ Radwell, AJR090317C.

##### Type deposition.

Holotype (1♀), allotype (1♂), and three paratypes (3♂) deposited at the CNC; four paratypes (1♀, 3♂) at the ACUA.

##### Diagnosis.

*Testudacarus radwellae* and *Testudacarus vulgaris* are the only testudacarines known to occur in Arkansas. *Testudacarus radwellae* are conspicuously violet over the entirety of their body, whereas the violet coloration of *Testudacarus vulgaris* is less vibrant and often absent, particularly on the platelets, legs, and secondary sclerotization of the venter. Males of *Testudacarus radwellae* also have dorsal-glandularia-4 located far lateral to the muscle scars, unlike others in the complex.

##### Description.

**Female (n=2)** with characteristics of the genus with following specifications.

Gnathosoma — Subcapitulum [153–155 ventral length; 117–133 dorsal length; 88–97 tall] ovoid with short rostrum. Chelicerae [148–156 long] unmodified with lightly curved fangs [28–29 long]. Pedipalp [177–187 long] unmodified and violet. Trochanter [27–30 long; 26–29 wide]. Femur [46–51 long; 35–38 wide]. Genu [38–42 long; 27–28 wide]. Tibia [44–49 long; 19–20 wide]. Tarsus [18–19 long; 10–11 wide].

**Figure 21.**
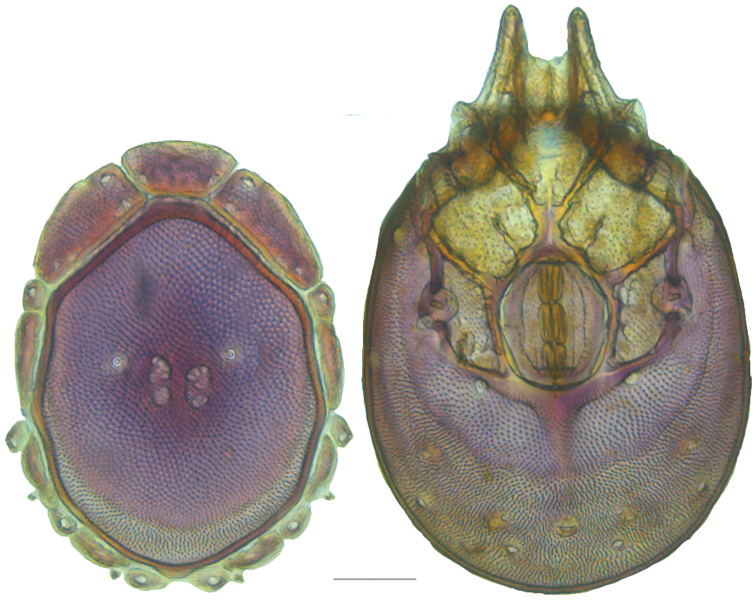
*Testudacarus radwellae* female: (**Left**) dorsum; (**Right**) venter. Scale: 100 µm.

Dorsum (Fig. 21) — [556–568 long; 425–444 wide] round to ovoid, completely violet to red–violet in color. Dorsal plate [463–473 long; 366–367 wide]. Primary sclerotization [389–415 long]. Dorso-glandularia-4 [128–132 apart] just anterior to [0–10] and lateral to [33] muscle scars. Platelets completely red–violet including all three anterior platelets. Anterio-medial platelet [134–142 long; 75–81 wide] rounded trapezoid. Anterio-lateral platelets [150–167 long; 69–78 wide]. Lateral platelets as follows: lateral-1 [47–49 long; 28–29 wide]; lateral-2 [113–114 long; 28–34 wide]; lateral-3 [40–47 long; 25–26 wide]; lateral-4 [97–99 long; 25–26 wide]; lateral-5 [38–55 long; 20–28 wide]; lateral-6 [80–83 long; 21–22 wide]; lateral-7 [49–56 long; 25–28 wide].

Venter (Fig. 21) — [717–726 long; 460–476 wide] round to ovoid and completely violet. Primary sclerotization [580–589 long]. Gnathosomal bay [64–72 dorsal length; 148–154 ventral length; 54–59 wide]. Coxal field [442–451 long; 303–309 wide]. Coxa-I [246–250 long; 92–102 midlength]. Coxa-II + III [118–125 distance to top of coxa-II; 181–183 distance to top of coxa-III; 332–335 distance to bottom of coxa-III; 210–214 total length]. Coxa-IV [300–304 distance to top; 142–147 total length]. Genital field [308–311 distance to top; 470–472 distance to bottom; 161–162 total length; 134–136 width; 154–163 distance from gnathosomal bay; 61–62 distance from coxa-I; 156–158 distance to excretory pore; 244–256 distance to caudad]. Distance to excretory pore [628–629].

Legs — violet. Total leg and podomere lengths as follows: Leg-I [464–466 total; trochanter 57–58; basifemur 81–82; telofemur 65–68; genu 83–84; tibia 88–90; tarsus 86–87]. Leg-II [489–490 total; trochanter 54–55; basifemur 81–83; telofemur 64–66; genu 86–87; tibia 97–101; tarsus 102–105]. Leg-III [559–564 total; trochanter 57–58; basifemur 77–85; telofemur 73–76; genu 102–105; tibia 116–117; tarsus 126–130]. Leg-IV [760–767 total; trochanter 86–87; basifemur 107–108; telofemur 108–109; genu 145–146; tibia 158–159; tarsus 152–159].

**Male (n=7)** similar to female except for sexually dimorphic characters previously discussed and with following specifications.

Gnathosoma — Subcapitulum [132–143 ventral length; 85–90 dorsal length; 81–86 tall]. Chelicerae [107–115 long]. Fangs [25–28 long]. Pedipalp [170–181 long]. Trochanter [25–27 long; 28–30 wide]. Femur [45–52 long; 33–35 wide]. Genu [38–39 long; width 27–29 wide]. Tibia [45–50 long; 18–21 wide]. Tarsus [14–17 long; 8–11 wide].

**Figure 22.**
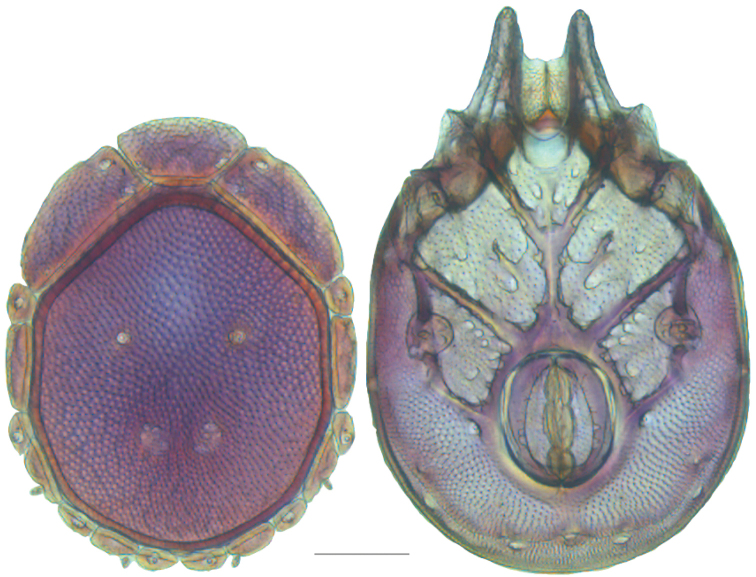
*Testudacarus radwellae* male: (**Left**) dorsum; (**Right**) venter. Scale: 100 µm.

Dorsum (Fig. 22) — [454–478 long; 330–372 wide]. Dorsal plate [376–405 long; 296–321 wide] without secondary sclerotization. Dorso-glandularia-4 [99–127 apart] far anterior to [74–83] and slightly lateral to [11–27] muscle scars. Anterio-medial platelet [119–138 long; 71–74 wide]. Anterio-lateral platelet [145–163 long; 64–72 wide]. Lateral platelets as follows: lateral-1 [36–45 long; 27–31 wide]; lateral-2 [89–99 long; 24–30 wide]; lateral-3 [39–44 long; 16–25 wide]; lateral-4 [64–77 long; 17–27 wide]; lateral-5 [38–49 long; 17–24 wide]; lateral-6 [48–56 long; 19–22 wide]; lateral-7 [38–45 long; 19–22 wide].

Venter (Fig. 22) — [575–606 long; 369–400 wide]. Primary sclerotization [536–555 long]. Gnathosomal bay [49–66 dorsal length; 130–137 ventral length; 48–56 wide]. Coxal field [405–424 long; 281–305 wide]. Coxa-I [223–238 long; 90–102 midlength]. Coxa-II + III [100–113 distance to top of coxa-II; 156–169 distance to top of coxa-III; 326–346 distance to bottom of coxa-III; 223–244 total length]. Coxa-IV [270–283 length to top; 126–146 total length]. Genital field [343–366 distance to top; 485–510 distance to bottom; 139–146 total length; 115–123 width; 210–232 distance from gnathosomal bay; 90–102 distance from coxa-I; 44–54 distance to excretory pore; 87–101 distance to caudad]. Genital skeleton [179–182 long; 94–103 wide]. Distance to excretory pore [536–555].

Legs — total leg and podomere lengths as follows: Leg-I [440–454 total; trochanter 53–58; basifemur 76–80; telofemur 58–67; genu 75–80; tibia 84–89; tarsus 82–90]. Leg-II [464–478 total; trochanter 52–57; basifemur 75–80; telofemur 58–62; genu 78–86; tibia 94–97; tarsus 99–103]. Leg-III [512–535 total; trochanter 49–55; basifemur 74–83; telofemur 62–69; genu 93–96; tibia 106–116; tarsus 110–125]. Leg-IV [699–726 total; trochanter 77–87; basifemur 101–110; telofemur 99–108; genu 130–133; tibia 144–148; tarsus 133–147].

##### Etymology.

Specific epithet *radwellae* after the late Dr Andrea J. Radwell, the American water mite researcher who collected the specimens needed for this description. Dr Radwell collaborated with us on the larger torrenticolid project as a whole, giving us invaluable advice and mentorship. Without her, large portions of this project would not have been possible. She is dearly missed.

##### Distribution.

Reported from only two counties (Polk and Montgomery) in Arkansas.

### *Testudacarus hitchensi* complex

**Species complex diagnosis.** Only this complex and the *Testudacarus minimus* complex are present east of the Great Plains. These two complexes resemble each other morphologically in many respects, but can be easily distinguished because members of this complex have non-uniform coloration across all three anterior platelets (colorless anterio-medial platelet and colored anterio-lateral platelets) while *Testudacarus minimus*-like mites possess uniform coloration across all three platelets. Males of this complex differ from *Testudacarus minimus*-like mites in having dorso-glandularia-4 positioned more anterior to and less lateral to the muscle scars. These mites are common in eastern United States and rare in eastern Canada and Florida, small (female and male dorsal length less than 700 and 600 µm, respectively), and violet to blue in color. This complex comprises the following species: *Testudacarus harrisi*, *Testudacarus dennetti*, *Testudacarus dawkinsi*, and *Testudacarus hitchensi*.

**Figure 23.**
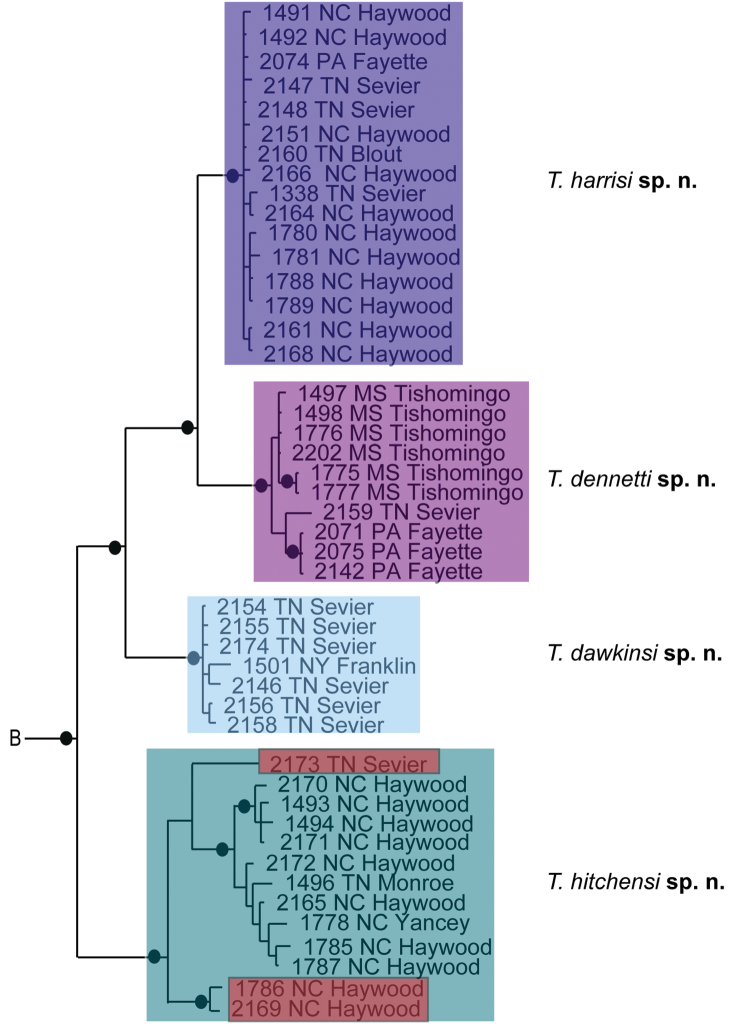
*Testudacarus hitchensi* complex molecular phylogeny: 28S and COI Bayesian analysis showing strong support for at least four distinct clades, but suggesting more (●: >95% posterior probability); excepting green clade, clades exhibit <1.5% divergence in COI within and >6% between; green clade exhibits <4.5% within and >9.5% between other clades; specimens in red constitute additional suspected species based on genetic data, but lack morphological or distributional variation from green clade; continuation of (**B**) lineage from Fig. 6.

**Remarks.** Distinguishable morphological characters can be found for four lineages while genetic data indicates more diversity (Fig. 23), suggesting cryptic speciation within the clade. Three clades (violet and blue clades in Fig. 23) exhibit less than 1.5% COI divergence within the clade and greater than 6% divergence between clades. This relatively low divergence within clades over their large ranges compared to the high divergence exhibited between clades even in the same streams strongly supports multiple species. The fourth clade (green in Fig. 23) proves problematic as no morphological variability has been found within the clade, but COI divergence of up to 4.5% is present and within a small geographic area (North Carolina and Tennessee). Ethanol collections were limited from this region and more data is needed. Furthermore, examinations of GAW collections provided by the CNC suggest there are other potential “morphotypes” of this species complex unrepresented in the genetic data presented. More species almost certainly exist in this complex, and further research is needed.

### Testudacarus hitchensi

Taxon classificationAnimaliaTrombidiformesTorrenticolidae

O’Neill & Dowling sp. n.

http://zoobank.org/0A6954E1-84CF-4F79-B966-B9F6E0587739

#### Type series.

**Holotype (1♀): North Carolina, USA**: 1♀ from Haywood, Great Smoky Mountains National Park, Cataloochee Creek, beside Mount Sterling Road at Hannah Hoglen Cemetery site (35°38'29.00"N, 83°3'22.00"W), 22 September 2010, by IM Smith, IMS100154 (Specimen 141898 – DNA#1493); **Paratypes (9♀, 10♂): North Carolina, USA**: (allotype) 1♂ from Haywood, Great Smoky Mountains National Park, Cataloochee Creek, beside Mount Sterling Road at Hannah Hoglen Cemetery site (35°38'29.00"N, 83°3'22.00"W), 22 September 2010, by IM Smith, IMS100154 (Specimen 146756 – DNA#2171); 1♀ and 2♂ from Haywood, Great Smoky Mountains National Park, Cataloochee Creek, beside Mount Sterling Road at Hannah Hoglen Cemetery site (35°38'29.00"N, 83°3'22.00"W), 22 September 2010, by IM Smith, IMS100154; 2♀ and 1♂ from Haywood County, Great Smoky Mountains National Park, Cataloochee Creek, beside Mount Sterling Road near bridge 1.7km north of road to campground (35°38'45.00"N, 83°4'32.00"W), 20 September 2010, by IM Smith, IMS100150; 2♂ from Macon County, Rainbow Springs, beside Forest Road 67 4.4 km south of Standing Indian Campground (35°3'6.00"N, 83°30'45.00"W), 1 July 2006, by IM Smith, IMS060040; 2♂ from Yancey County, Pisgah National Forest, South Toe River, Lost Cove beside Toe River Road (Forest Road 472) 0.4km east of Forest Road 2074 (35°45'0.00"N, 82°12'53.00"W), 9 September 2007, IM Smith, IMS070059; 1♀ from Yancey County, Pisgah National Forest, South Toe River, Lost Cove Picnic Area beside Toe River Road (Forest Road 472) 2.8 km east of Route 80 (35°45'13.00"N, 82°12'42.00"W), 27 September 2009, by IM Smith, IMS090127; **Tennessee, USA**: 1♂ from Monroe, beside Forest Route #35 2.3km northeast of road from Route 165 to Miller Chapel Baptist Church (35°21'47.00"N, 84°9'47.00"W), 12 September 2009, by IM Smith, IMS090112; 3♀ and 1♂ from Sevier County, Great Smoky Mountains National Park, Bullhead Branch, Sugarlands Nature Trail off Route 441/71 (35°40'47.00"N, 83°31'52.00"W), 7 September 2009, by IM Smith, IMS090101; 1♀ from Sevier County, Great Smoky Mountains National Park, Bullhead Branch, Sugarlands Nature Trail off Route 441/71 (35°40'48.00"N, 83°31'53.00"W), 3 September 2009, by IM Smith, IMS090095; **Georgia, USA**: 1♀ from Floyd County, beside road from Everrett Springs to Villanow 1.4 km south of The Pocket Recreation Area, 4 July 1990, by IM Smith, IMS900077.

#### Paratypes examined but measurements not included.

**(1♀, 2♂): North Carolina, USA**: 1♀ from Haywood County, Great Smoky Mountains National Park, Cataloochee Creek, beside Mount Sterling Road near bridge 1.7km north of road to campground (35°38'45.00"N, 83°4'32.00"W), 20 September 2010, by IM Smith, IMS100150; 1♂ from Haywood County, Great Smoky Mountains National Park, tributary of Hemphill Creek, Appalachian Highlands Science Learning Center near Ferguson Cabin site, (35°34'56.00"N, 83°4'30.00"W), 21 September 2010, by IM Smith, IMS100153; **Tennessee, USA**: 1♀ from Sevier County, Great Smoky Mountains National Park, Catron Branch, Elkmont Road off Little River Road (35°39'51.00"N, 83°35'19.00"W), 24 September 2010, IMS100156.

#### Type deposition.

Holotype (1♀), allotype (1♂), and eight paratypes (4♀, 4♂) deposited at CNC; ten paratypes (5♀, 5♂) at ACUA.

#### Diagnosis.

These mites differ from all others in the complex in having large medial pores on the dorsal plate surrounded by a ring of smaller pores (all pores uniform in other species). Males also have a “bleached” or colorless area posterior to the coxal plate that is colored in other members of the complex.

#### Description.

**Female (n=10)** with characteristics of genus with following specifications.

Gnathosoma — Subcapitulum [165–175 ventral length; 99–106 dorsal length; 90–100 tall] ovoid with short rostrum. Chelicerae [139–150 long] unmodified with lightly curved fangs [29–32 long]. Pedipalp [192–205 long] unmodified. Trochanter [25–28 long; 29–32 wide]. Femur [54–57 long; 37–40 wide]. Genu [40–46 long; 29–33 wide]. Tibia [51–55 long; 20–23 wide]. Tarsus [19–21 long; 10–11 wide].

**Figure 24.**
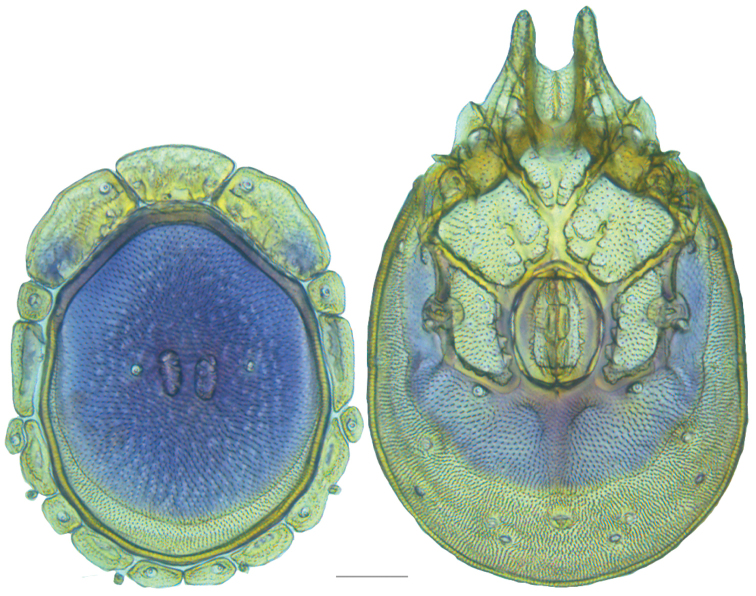
*Testudacarus hitchensi* female: (**Left**) dorsum; (**Right**) venter. Scale: 100 µm.

Dorsum (Fig. 24) — [591–669 long; 445–504 wide] round to ovoid. Dorsal plate [485–556 long; 375–424 wide] with noticeable pore variation: medial pores large surrounded by smaller distal pores. Primary sclerotization [425–470 long] violet to blue. Dorso-glandularia-4 [124–175 apart] lateral to [19–43] and around the anterior tips of the muscle scars. Platelets violet to blue or colorless. Anterio-medial platelet [146–168 long; 81–101 wide] colorless rounded trapezoid noticeably smaller than anterio-lateral platelets. Anterio-lateral platelet [170–197 long; 89–102 wide] with violet to blue restricted to posterior half or third of the platelet. Lateral platelets as follows: lateral-1 [53–69 long; 46–57 wide]; lateral-2 [125–140 long; 35–52 wide]; lateral-3 [39–53 long; 20–27 wide]; lateral-4 [96–115 long; 32–43 wide]; lateral-5 [50–62 long; 29–39 wide]; lateral-6 [81–96 long; 29–43 wide]; lateral-7 [61–77 long; 27–33 wide].

Venter (Fig. 24) — [765–870; 482–553 wide] round to ovoid. Primary sclerotization [631–717 long] violet to blue. Gnathosomal bay [71–90 dorsal length; 149–170 ventral length; 53–62 wide]. Coxal field [482–543 long; 325–409 wide]. Coxa-I [256–289 long; 99–126 midlength]. Coxa-II + III [118–140 distance to top of coxa-II; 187–215 distance to top of coxa-III; 347–401 distance to bottom of coxa-III; 224–264 total length]. Coxa-IV [333–375 distance to top; 139–168 total length]. Genital field [329–382 distance to top; 493–542 distance to bottom; 158–172 total length; 125–150 width; 178–212 distance from gnathosomal bay; 69–100 distance from coxa-I; 175–234 distance to excretory pore; 272–349 distance to caudad]. Eggs [150–168 long; 1–4 eggs]. Distance to excretory pore [688–777].

Legs — orange and restricted violet to blue. Total leg and podomere lengths as follows: Leg-I [473–524 total; trochanter 60–62; basifemur 83–93; telofemur 65–76; genu 86–95; tibia 92–105; tarsus 83–95]. Leg-II [501–552 total; trochanter 54–63; basifemur 83–93; telofemur 65–72; genu 88–99; tibia 101–111; tarsus 102–115]. Leg-III [586–635 total; trochanter 61–65; basifemur 89–100; telofemur 70–80; genu 105–113; tibia 122–137; tarsus 132–144]. Leg-IV [805–876 total; trochanter 93–109; basifemur 115–132; telofemur 115–125; genu 151–167; tibia 167–180; tarsus 158–177].

**Male (n=10)** similar to female except for sexually dimorphic characters previously discussed and with following specifications.

Gnathosoma — Subcapitulum [150–160 ventral length; 95–106 dorsal length; 86–95 tall]. Chelicerae 127–139 long]. Fangs [26–29 long]. Pedipalp [180–195long]. Trochanter [25–27 long; 27–30 wide]. Femur [50–55 long; 34–37 wide]. Genu [38–41 long; width 26–29 wide]. Tibia [49–52 long; 19–22 wide]. Tarsus [17–20 long; 9–11 wide].

**Figure 25.**
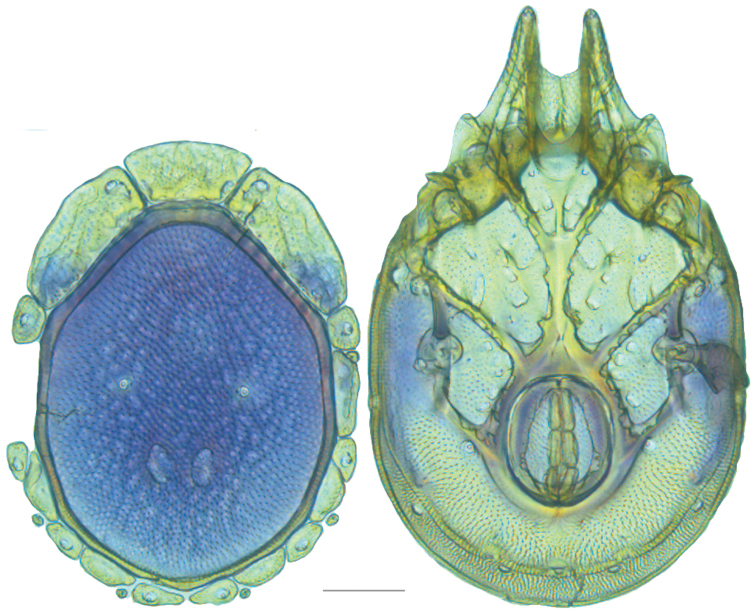
*Testudacarus hitchensi* male: (**Left**) dorsum; (**Right**) venter. Scale: 100 µm.

Dorsum (Fig. 25) — [491–567 long; 387–436 wide]. Dorsal plate [404–474 long; 326–375 wide]. Dorso-glandularia-4 [116–152 apart] far anterior to [53–75] and lateral to [13–32] muscle scars. Anterio-medial platelet [137–152 long; 71–91 wide]. Anterio-lateral platelets [163–184 long; 74–88 wide]. Lateral platelets as follows: lateral-1 [45–54 long; 37–44 wide]; lateral-2 [101–120 long; 34–41 wide]; lateral-3 [39–50 long; 19–32 wide]; lateral-4 [74–110 long; 30–35 wide]; lateral-5 [46–58 long; 25–33 wide]; lateral-6 [53–75 long; 27–34 wide]; lateral-7 [46–62 long; 24–33 wide].

Venter (Fig. 25) — [641–718 long; 418–481 wide]. Primary sclerotization [593–671 long]. Gnathosomal bay [62–89 dorsal length; 131–164 ventral length; 45–67 wide]. Coxal field [441–500 long; 309–340 wide]. Coxa-I [233–276 long; 95–114 midlength]. Coxa-II + III [105–128 distance to top of coxa-II; 171–202 distance to top of coxa-III; 357–409 distance to bottom of coxa-III; 245–288 total length]. Coxa-IV [304–355 length to top; 127–159 total length]. Genital field [378–440 distance to top; 524–598 distance to bottom; 143–157 total length; 115–131 width; 239–284 distance from gnathosomal bay; 143–173 distance from coxa-I; 55–91 distance to excretory pore; 110–153 distance to caudad]. Genital skeleton [190–207 long; 115–126 wide]. Distance to excretory pore [593–671].

Legs — total leg and podomere lengths as follows: Leg-I [444–508 total; trochanter 55–62; basifemur 75–89; telofemur 63–73; genu 80–91; tibia 85–99; tarsus 84–96]. Leg-II [474–533 total; trochanter 60–64; basifemur 77–90; telofemur 61–71; genu 82–93; tibia 92–106; tarsus 99–113]. Leg-III [537–598 total; trochanter 57–64; basifemur 80–92; telofemur 65–73; genu 96–108; tibia 113–128; tarsus 121–137]. Leg-IV [721–778 total; trochanter 88–99; basifemur 96–117; telofemur 102–113; genu 135–151; tibia 147–168; tarsus 142–156].

#### Etymology.

Specific epithet *hitchensi* after the late Christopher Eric Hitchens, the English author, journalist, and literary critic. As Sam Harris’ wife, Annaka, said: “Nothing Hitchens does is ever boring.” Hitchens has inspired thousands of free-thinkers to remain clever and engaged in our attempts to understand the world around us.

#### Distribution.

Eastern United States east of the Mississippi River with southern limits in Florida.

#### Remarks.

As it is likely that this species represents a cryptic species complex, measurements were only included from specimens exhibiting less than 2% COI divergence within the clade (those highlighted in red in Fig. 23 were excluded). This was done so measurements would remain useful if more species were diagnosed in the future.

### Testudacarus harrisi

Taxon classificationAnimaliaTrombidiformesTorrenticolidae

O’Neill & Dowling sp. n.

http://zoobank.org/EDE0FB53-D060-4879-8628-7DBBBF1749EA

#### Type series.

**Holotype (1♀): North Carolina, USA**: 1♀ from Haywood County, Great Smoky Mountains National Park, Cataloochee Creek, beside Mount Sterling Road near bridge 1.7km north of road to campground (35°38'45.00"N, 83°4'32.00"W), 20 September 2010, by IM Smith, IMS100150 (Specimen 146752 – DNA#2166); **Paratypes (12♀, 10♂): North Carolina, USA**: (allotype) 1♂ from Haywood County, Great Smoky Mountains National Park, Cataloochee Creek, beside Mount Sterling Road near bridge 1.7km north of road to campground (35°38'45.00"N, 83°4'32.00"W), 20 September 2010, by IM Smith, IMS100150 (Specimen 146750 – DNA#2164); **Tennessee, USA**: 1♂ from Sevier County, Great Smoky Mountains National Park, Crosby Creek, Cosby Recreation Area beside Cosby Campground Road 0.3km from Route 321 (35°46'54.00"N, 83°13'2.00"W), 16 September 2010, by IM Smith, IMS100140; 2♀ and 2♂ from Sevier County, Great Smoky Mountains National Park, Bullhead Branch, Sugarlands Nature Trail off Route 441/71 (35°40'47.00"N, 83°31'52.00"W), 7 September 2009, by IM Smith, IMS090101; 1♀ from Sevier County, Great Smoky Mountains National Park, Bullhead Branch, Sugarlands Nature Trail off Route 441/71 (35°40'48.00"N, 83°31'53.00"W), 3 September 2009, by IM Smith, IMS090095; 1♂ from Blount County, Great Smoky Mountains National Park, Cades Cove, near parking lot for Abrams Falls Trail (35°35'26.00"N, 83°51'10.00"W), 17 September 2010, by IM Smith, IMS100143; 2♀ from Sevier Co, Great Smoky Mountains National Park, Bullhead Branch, Sugarlands Nature Trail off Route 441/71 (35°40'47.00"N, 83°31'51.00"W), 10 September 2010, by IM Smith, IMS100125; **North Carolina, USA**: 2♀ and 2♂ from Haywood County, Great Smoky Mountains National Park, Cataloochee Creek, beside Cataloochee Road 0.3km north of Cataloochee Campground (35°38'1.00"N, 83°5'2.00"W), 6 September 2009, IMS090097; 2♀ and 1♂ from Haywood County, Great Smoky Mountains National Park, Big Creek, Waterville Big Creek Picnic Area (35°44'59.00"N, 83°6'42.00"W), 16 September 2010, by IM Smith, IMS100138; 1♀ and 1♂ from Haywood County, Great Smoky Mountains National Park, Cataloochee Creek, beside Mount Sterling Road near bridge 1.7km north of road to campground (35°38'45.00"N, 83°4'32.00"W), 20 September 2010, by IM Smith, IMS100150; 1♂ from Yancey County, Pisgah National Forest, South Toe River, Lost Cove beside Toe River Road (Forest Road 472) 0.4km east of Forest Road 2074 (35°45'0.00"N, 82°12'53.00"W), 9 September 2007, IM Smith, IMS070059; 1♀ from Haywood County, Great Smoky Mountains National Park, Rough Fork Creek, beside road to Nellie 0.3 km west of Pretty Hollow Gap Trailhead (35°37'31.00"N, 83°6'46.00"W), 20 September 2010, by IM Smith, IMS100148; **Pennsylvania, USA**: 1♀ from Fayette County, State Game Lands #51, Dunbar Creek, off Furnace Hill Road east of Dunbar (39°57'50.00"N, 79°35'8.70"W), 10 August 2014, MJ Skvarla, MS14-0810-001.

#### Type deposition.

Holotype (1♀), allotype (1♂) and ten paratypes (5♀, 5♂) deposited at Canadian National Collection; eleven paratypes (7♀, 4♂) at ACUA.

#### Diagnosis.

These mites have violet to blue coloration over the majority of their anterio-lateral platelets while the rest of the complex have coloration restricted to the posterior half of the platelet.

#### Description.

**Female (n=13)** with characteristics of the genus with following specifications.

Gnathosoma — Subcapitulum [143–165 ventral length; 90–105 dorsal length; 84–95 tall] ovoid with short rostrum. Chelicerae [119–136 long] unmodified with lightly curved fangs [24–32 long]. Pedipalp [167–191 long] unmodified. Trochanter [23–30 long; 29–31 wide]. Femur [47–53 long; 33–38 wide]. Genu [37–42 long; 27–30 wide]. Tibia [40–53 long; 17–22 wide]. Tarsus [15–20 long; 9–12 wide].

**Figure 26.**
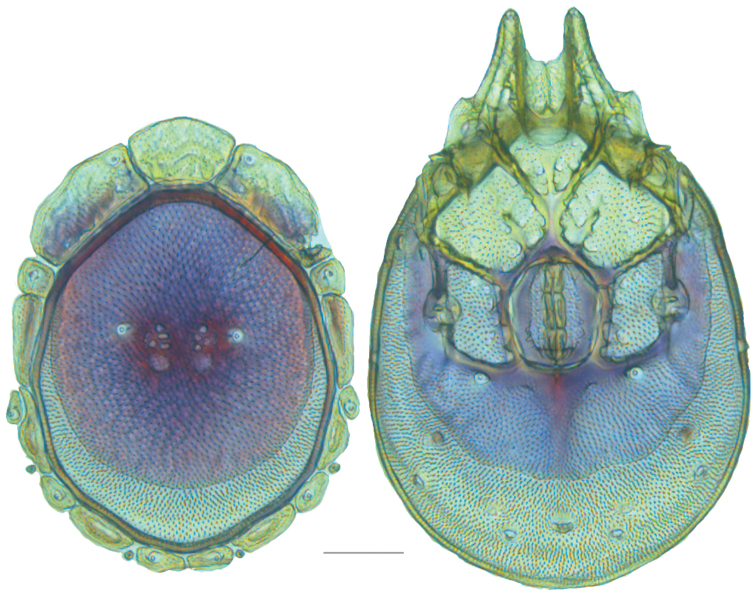
*Testudacarus harrisi* female: (**Left**) dorsum; (**Right**) venter. Scale: 100 µm.

Dorsum (Fig. 26) — [527–617 long; 420–482 wide] ovoid. Dorsal plate [375–495 long; 355–515 wide]. Primary sclerotization [358–419 long] violet to blue. Dorso-glandularia-4 [113–167 apart] lateral to [16–42] and around the anterior tips of the muscle scars. Platelets violet to blue or clear. Anterio-medial platelet [112–144 long; 70–94 wide] colorless rounded trapezoid noticeably smaller than anterio-lateral platelets. Anterio-lateral platelets [156–183 long; 74–86 wide] mostly violet to blue with anterio-most corner generally colorless; anterio-medial corner often with orange spot. Lateral platelets as follows: lateral-1 [38–50 long; 35–44 wide]; lateral-2 [103–133 long; 30–40 wide]; lateral-3 [29–45 long; 16–30 wide]; lateral-4 [90–119 long; 26–37 wide]; lateral-5 [47–64 long; 22–34 wide]; lateral-6 [65–90 long; 27–34 wide]; lateral-7 [56–69 long; 24–36 wide].

Venter (Fig. 26) — [668–786 long; 453–509 wide] round to ovoid. Primary sclerotization violet to blue. Gnathosomal bay [61–78 dorsal length; 128–156 ventral length; 45–56 wide]. Coxal field [418–480 long; 281–363 wide]. Coxa-I [213–254 long; 85–105 midlength]. Coxa-II + III [109–131 distance to top of coxa-II; 170–195 distance to top of coxa-III; 295–356 distance to bottom of coxa-III; 186–234 total length]. Coxa-IV [291–335 distance to top; 125–154 total length]. Genital field [284–335 distance to top; 426–489 distance to bottom; 139–154 total length; 114–127 width; 152–184 distance from gnathosomal bay; 65–82 distance from coxa-I; 167–207 distance to excretory pore; 241–307 distance to caudad]. Distance to excretory pore [593–693].

Legs — violet to blue and orange. Total leg and podomere lengths as follows: Leg-I [449–485 total; trochanter 54–62; basifemur 77–83; telofemur 62–70; genu 80–90; tibia 89–99; tarsus 80–90]. Leg-II [471–510 total; trochanter 54–60; basifemur 74–84; telofemur 61–66; genu 82–94; tibia 98–107; tarsus 87–109]. Leg-III [548–612 total; trochanter 55–64; basifemur 79–91; telofemur 66–74; genu 96–114; tibia 116–137; tarsus 119–141]. Leg-IV [737–825 total; trochanter 79–99; basifemur 103–123; telofemur 103–121; genu 138–154; tibia 154–169; tarsus 147–167].

**Male (n=10)** similar to female except for sexually dimorphic characters previously discussed and with following specifications.

Gnathosoma — Subcapitulum [133–144 ventral length; 83–90 dorsal length; 72–84 tall]. Chelicerae [110–120 long]. Fangs [25–30 long]. Pedipalp [168–183 long]. Trochanter [22–25 long; 25–29 wide]. Femur [45–50 long; 32–36 wide]. Genu [36–40 long; width 24–30 wide]. Tibia [44–52 long; 18–20 wide]. Tarsus [16–20 long; 8–11 wide].

**Figure 27.**
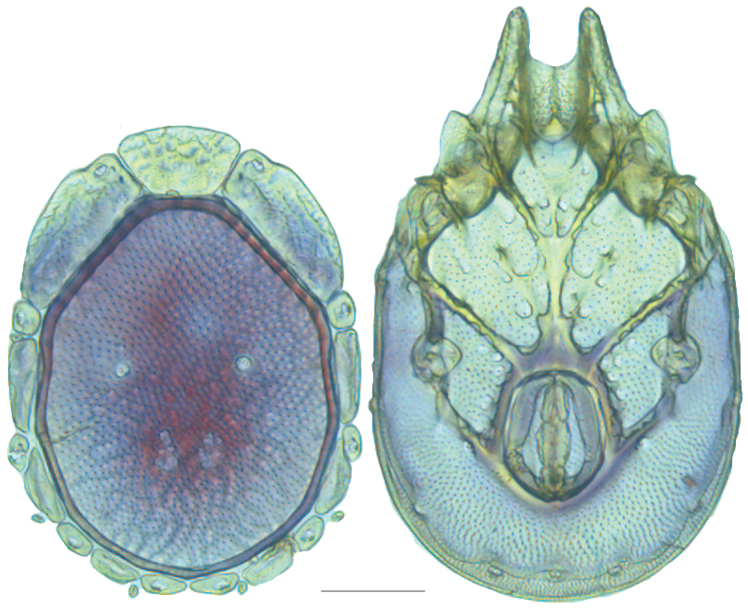
*Testudacarus harrisi* male: (**Left**) dorsum; (**Right**) venter. Scale: 100 µm.

Dorsum (Fig. 27) — [418–488 long; 312–380 wide]. Dorsal plate [340–402 long; 271–322 wide]. Dorso-glandularia-4 [89–129 apart] far anterior to [31–71] and lateral to [12–24] muscle scars. Anterio-medial platelet [111–132 long; 63–80 wide]. Anterio-lateral platelets [141–164 long; 63–79 wide]. Lateral platelets as follows: lateral-1 [30–38 long; 29–32 wide]; lateral-2 [85–96 long; 24–33 wide]; lateral-3 [30–40 long; 15–25 wide]; lateral-4 [61–78 long; 21–32 wide]; lateral-5 [35–44 long; 18–27 wide]; lateral-6 [38–56 long; 19–27 wide]; lateral-7 [39–50 long; 17–29 wide].

Venter (Fig. 27) — [544–612 long; 346–399 wide]. Primary sclerotization [504–578 long]. Gnathosomal bay [49–64 dorsal length; 119–133 ventral length; 48–54 wide]. Coxal field [387–443 long; 272–316 wide]. Coxa-I [210–229 long; 88–96 midlength]. Coxa-II + III [97–112 distance to top of coxa-II; 156–173 distance to top of coxa-III; 312–346 distance to bottom of coxa-III; 215–240 total length]. Coxa-IV [267–297 length to top; 120–154 total length]. Genital field [329–369 distance to top; 451–501 distance to bottom; 121–132 total length; 97–104 width; 208–238 distance from gnathosomal bay; 119–143 distance from coxa-I; 53–79 distance to excretory pore; 93–111 distance to caudad]. Genital skeleton [150–167 long; 77–92 wide]. Distance to excretory pore [504–578].

Legs — total leg and podomere lengths as follows: Leg-I [419–451 total; trochanter 45–56; basifemur 70–77; telofemur 58–68; genu 74–82; tibia 81–90; tarsus 79–84]. Leg-II [429–472 total; trochanter 47–52; basifemur 69–77; telofemur 56–63; genu 76–86; tibia 86–98; tarsus 93–99]. Leg-III [491–540 total; trochanter 49–53; basifemur 70–86; telofemur 59–66; genu 89–98; tibia 107–120; tarsus 114–124]. Leg-IV [665–739 total; trochanter 74–90; basifemur 95–109; telofemur 95–108; genu 128–138; tibia 139–150; tarsus 131–145].

#### Etymology.

Specific epithet after Samuel Benjamin Harris, the American author, philosopher, and co-founder of Project Reason. Sam Harris, more than any speaker and author, has challenged my (JCO) views and assumptions and kept me on my toes.

#### Distribution.

Eastern United States east of the Mississippi river, with southern limits in Florida.

### Testudacarus dennetti

Taxon classificationAnimaliaTrombidiformesTorrenticolidae

O’Neill & Dowling sp. n.

http://zoobank.org/C112BB32-CAD0-42A4-A757-5C318FAF067C

#### Type series.

**Holotype (1♀): Pennsylvania, USA**: 1♀ from Fayette County, Ohiopyle State Park, Laurel Run, fishing access #2 off T798 (Meadow Run Rd) Ohiopyle State Park (39°50'58.00"N, 79°30'51.00"W), 10 August 2014, by MJ Skvarla, MS14-0810-005 (Specimen 143645 – DNA#2071); **Paratypes (8♀, 7♂): Mississippi, USA**: (allotype) 1♂ from Tishomingo County, Tishomingo State Park, Rocky Quarry Branch, beside road just outside park entrance (34°36'43.00"N, 88°12'4.00"W), 20 September 2009, by IM Smith, IMS090115 (Specimen 146784 – DNA#2202); 3♀ and 4♂ from Tishomingo County, Tishomingo State Park, Rocky Quarry Branch, beside road just outside park entrance (34°36'43.00"N, 88°12'4.00"W), 20 September 2009, by IM Smith, IMS090115; 2♀ and 2♂ from Tishomingo County, Tishomingo State Park, Rocky Quarry Branch, (34°36’” N, 88°11'W), 18 September 1991, by IM Smith, IMS910049; **Pennsylvania, USA**: 2♀ from Fayette County, State Game Lands #51, Dunbar Creek, off Furnace Hill Road east of Dunbar (39°57'50.00"N, 79°35'8.70"W), 10 August 2014, MJ Skvarla, MS14-0810-001; Alabama, USA: 1♀ from DeKalb County, Desoto State Park, beside Trail Y (Yellow) (34°29'N, 85°32'W), 26 September 1992, by IM Smith, IMS920053A.

#### Type deposition.

Holotype (1♀), allotype (1♂), and six paratypes (3♀, 3♂) deposited at CNC; eight paratypes (5♀, 3♂) at ACUA.

#### Diagnosis.

Both *Testudacarus dennetti* and *Testudacarus dawkinsi* have dorsal plates with uniform pores (unlike *Testudacarus hitchensi*) and anterio-lateral platelets with color restricted to the posterior half (unlike *Testudacarus harrisi*). However, they can be distinguished based on size. *Testudacarus dennetti* females and males have dorsal lengths less than 575 and 450 µm, respectively. *Testudacarus dawkinsi* females and males have dorsal lengths greater than 600 and 475 µm, respectively.

#### Description.

**Female (n=9)** with characteristics of the genus with following specifications.

Gnathosoma — Subcapitulum [139–152 ventral length; 85–97 dorsal length; 85–91 tall] ovoid with short rostrum. Chelicerae [117–126 long] unmodified with lightly curved fangs [24–28 long]. Pedipalp [168–189 long] unmodified. Trochanter [23–27 long; 28–31 wide]. Femur [42–52 long; 33–35 wide]. Genu [35–41 long; 25–32 wide]. Tibia [45–52 long; 17–22 wide]. Tarsus [18–20 long; 8–12 wide].

**Figure 28.**
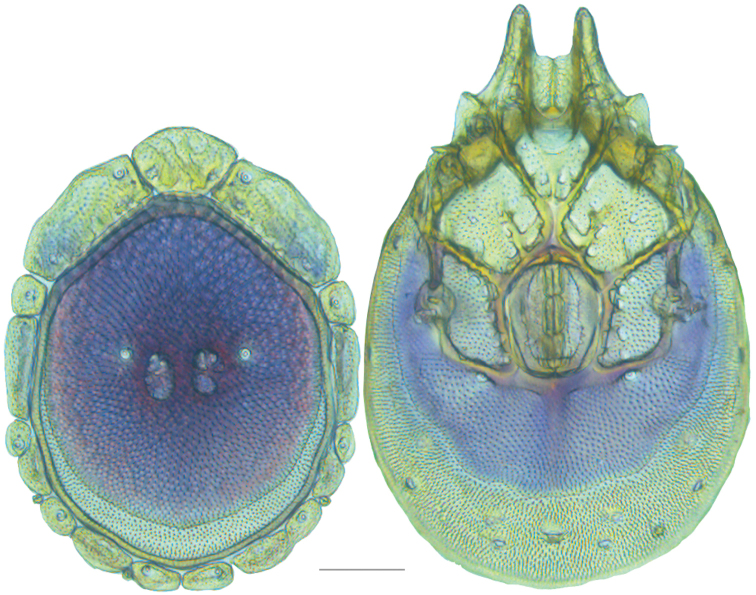
*Testudacarus dennetti* female: (**Left**) dorsum; (**Right**) venter. Scale: 100 µm.

Dorsum (Fig. 28) — [473–558 long; 368–429 wide] round to ovoid. Dorsal plate [348–459 long; 353–442 wide]. Primary sclerotization [319–400 long]. Dorso-glandularia-4 [121–150 apart] lateral to [16–41] and around the anterior tips of muscle scars. Platelets violet to blue or colorless. Anterio-medial platelet [115–128 long; 65–83 wide] colorless rounded trapezoid noticeably smaller than anterio-lateral platelets. Anterio-lateral platelets [150–171 long; 68–78 wide] with violet to blue restricted to posterior half or third of the platelet. Lateral platelets as follows: lateral-1 [36–48 long; 29–44 wide]; lateral-2 [96–129 long; 24–37 wide]; lateral-3 [26–44 long; 14–27 wide]; lateral-4 [68–95 long; 19–39 wide]; lateral-5 [39–56 long; 13–32 wide]; lateral-6 [65–81 long; 16–34 wide]; lateral-7 [42–69 long; 15–30 wide].

Venter (Fig. 28) — [627–738 long; 411–474 wide] round to ovoid. Primary sclerotization [534–600 long] violet to blue. Gnathosomal bay [61–70 dorsal length; 125–142 ventral length; 48–57 wide]. Coxal field [406–438 long; 286–320 wide]. Coxa-I [216–236 long; 89–103 midlength]. Coxa-II + III [103–116 distance to top of coxa-II; 164–171 distance to top of coxa-III; 298–321 distance to bottom of coxa-III; 195–208 total length]. Coxa-IV [281–301 distance to top; 125–142 total length]. Genital field [279–304 distance to top; 416–455 distance to bottom; 137–151 total length; 110–123 width; 149–170 distance from gnathosomal bay; 54–75 distance from coxa-I; 160–185 distance to excretory pore; 211–295 distance to caudad]. Distance to excretory pore [581–640].

Legs — violet to blue and orange. Total leg and podomere lengths as follows: Leg-I [431–463 total; trochanter 48–58; basifemur 70–78; telofemur 59–65; genu 77–84; tibia 85–93; tarsus 81–88]. Leg-II [455–487 total; trochanter 51–56; basifemur 72–79; telofemur 57–64; genu 80–88; tibia 92–102; tarsus 98–109]. Leg-III [538–572 total; trochanter 54–59; basifemur 73–83; telofemur 62–67; genu 95–106; tibia 114–127; tarsus 128–134]. Leg-IV [641–768 total; trochanter 84–89; basifemur 96–115; telofemur 102–110; genu 137–144; tibia 147–163; tarsus 142–158].

**Male (n=7)** similar to female except for sexually dimorphic characters previously discussed and with following specifications.

Gnathosoma — Subcapitulum [125–134 ventral length; 80–86 dorsal length; 74–83 tall]. Chelicerae [100–115 long]. Fangs [24–28 long]. Pedipalp [164–179 long]. Trochanter [22–24 long; 26–29 wide]. Femur [44–50 long; 30–35 wide]. Genu [36–43 long; width 25–28 wide]. Tibia [44–49 long; 17–20 wide]. Tarsus [17–19 long; 9–10 wide].

**Figure 29.**
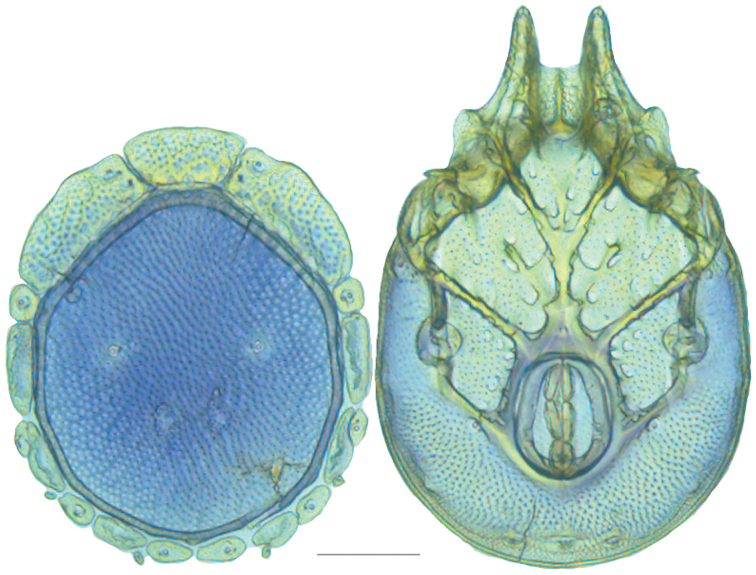
*Testudacarus dennetti* male: (**Left**) dorsum; (**Right**) venter. Scale: 100 µm.

Dorsum (Fig. 29) — [408–440 long; 333–351 wide]. Dorsal plate [333–370 long; 268–305 wide]. Dorso-glandularia-4 [98–131 apart] lateral to [15–32] and far anterior to [46–62] muscle scars. Anterio-medial platelet [104–123 long; 60–66 wide]. Anterio-lateral platelets [133–153 long; 59–69 wide]. Lateral platelets as follows: lateral-1 [29–35 long; 25–31 wide]; lateral-2 [80–101 long; 24–32 wide]; lateral-3 [27–35 long; 18–21 wide]; lateral-4 [56–78 long; 21–28 wide]; lateral-5 [32–42 long; 20–25 wide]; lateral-6 [46–54 long; 23–25 wide]; lateral-7 [30–47 long; 19–23 wide].

Venter (Fig. 29) — [537–570 long; 352–370 wide]. Primary sclerotization [498–536 long]. Gnathosomal bay [55–61 dorsal length; 110–129 ventral length; 42–53 wide]. Coxal field [378–417 long; 275–292 wide]. Coxa-I [195–219 long; 80–95 midlength]. Coxa-II + III [84–103 distance to top of coxa-II; 141–165 distance to top of coxa-III; 299–327 distance to bottom of coxa-III; 215–236 total length]. Coxa-IV [261–284 length to top; 117–133 total length]. Genital field [322–341 distance to top; 443–471 distance to bottom; 121–130 total length; 96–103 width; 207–222 distance from gnathosomal bay; 120–136 distance from coxa-I; 54–66 distance to excretory pore; 85–100 distance to caudad]. Genital skeleton [152–169 long; 80–95 wide]. Distance to excretory pore [498–536].

Legs — total leg and podomere lengths as follows: Leg-I [414–434 total; trochanter 47–54; basifemur 67–73; telofemur 55–62; genu 72–79; tibia 81–85; tarsus 79–85]. Leg-II [432–450 total; trochanter 48–54; basifemur 66–72; telofemur 54–61; genu 73–80; tibia 88–91; tarsus 96–99]. Leg-III [478–525 total; trochanter 49–58; basifemur 66–76; telofemur 58–64; genu 83–93; tibia 102–114; tarsus 114–126]. Leg-IV [658–685 total; trochanter 76–86; basifemur 85–103; telofemur 90–100; genu 124–130; tibia 140–141; tarsus 133–140].

#### Etymology.

Specific epithet *dennetti* after Daniel Clement Dennett III, the American philosopher, writer, and cognitive scientist. Dennett’s work has been the focus of many late night debates in close social circles just as he adds the necessary philosophical spice to the New Athiests.

#### Distribution.

Eastern United States east of the Mississippi River, with southern limits in Florida.

### Testudacarus dawkinsi

Taxon classificationAnimaliaTrombidiformesTorrenticolidae

O’Neill & Dowling sp. n.

http://zoobank.org/4AC3753F-9E9D-4B38-9045-1BA094040E16

#### Type series.

**Holotype (1♀): New York, USA**: 1♀ from Franklin County, Little Aldo Creek, Little Aldo Creek trail from Keese Mill Rd (44°25'32.00"N, 74°20'43.00"W), 19 July 2013, by AJ Radwell and C Milewski, AJR13-0719-205 (Specimen 141897 – DNA#1501); **Paratypes (5♀, 9♂): Tennessee, USA**: (allotype) 1♂ from Sevier County, Great Smoky Mountains National Park, Crosby Creek, Cosby Recreation Area beside Cosby Campground Road 0.3km from Route 321 (35°46'54.00"N, 83°13'2.00"W), 16 September 2010, by IM Smith, IMS100140 (Specimen 146744 – DNA#2156); 1♂ from Sevier County, Great Smoky Mountains National Park, Bullhead Branch, Sugarlands Nature Trail off Route 441/71 (35°40'47.00"N, 83°31'51.00"W), 10 September 2010, by IM Smith, IMS100125; 2♀ and 1♂ from Sevier County, Great Smoky Mountains National Park, Crosby Creek, Cosby Recreation Area beside Cosby Campground Road 0.3km from Route 321 (35°46'54.00"N, 83°13'2.00"W), 16 September 2010, by IM Smith, IMS100140; 1♂ from Sevier County, Great Smoky Mountains National Park, Bullhead Branch, Sugarlands Nature Trail off Route 441/71 (35°40'47.00"N, 83°31'52.00"W), 24 September 2010, by IM Smith, IMS100158; 1♂ from Sevier County, Great Smoky Mountains National Park, Bullhead Branch, Sugarlands Nature Trail off Route 441/71 (35°40'48.00"N, 83°31'53.00"W), 3 September 2009, by IM Smith, IMS090095; 1♀ and 1♂ from Sevier County, Great Smoky Mountains National Park, Bullhead Branch, Sugarlands Nature Trail off Route 441/71 (35°40'47.00"N, 83°31'52.00"W), 7 September 2009, by IM Smith, IMS090101; 1♀ and 2♂ from Sevier County, Great Smoky Mountains National Park, Cosby Creek, beside road to Cosby Campground at Gabes Mountain Trailhead (35°45'27.00"N, 83°12'36.00"W), 19 September, by IM Smith, IMS050093A; Virginia, USA: 1♂ from Alleghany County, Simpson Creek, Longdale Furnace beside Route 850 2.2 km northeast of I-64 overpass (37°49'41.00"N, 79°39'30.00"W), 14 August 2008, by IM Smith, IMS080044; **North Carolina, USA**: 1♀ from Macon County, Rainbow Springs, beside Forest Road 67 4.4 km south of Standing Indian Campground (35°3'6.00"N, 83°30'45.00"W), 1 July 2006, by IM Smith, IMS060040.

#### Type deposition.

Holotype (1♀), allotype (1♂), and six paratypes (3♀, 3♂) deposited at CNC; seven paratypes (2♀, 5♂) at ACUA.

#### Diagnosis.

Both *Testudacarus dennetti* and *Testudacarus dawkinsi* have dorsal plates with uniform pores (unlike *Testudacarus hitchensi*) and anterio-lateral platelets with color restricted to the posterior half (unlike *Testudacarus harrisi*). However, they can be distinguished based on size. *Testudacarus dennetti* females and males have dorsal lengths less than 575 and 450 µm, respectively. *Testudacarus dawkinsi* females and males have dorsal lengths greater than 600 and 475 µm, respectively.

#### Description.

**Female (n=6)** with characteristics of the genus with following specifications.

Gnathosoma — Subcapitulum [160–168 ventral length; 102–105 dorsal length; 91–95 tall] ovoid with short rostrum. Chelicerae [136–141 long] unmodified with lightly curved fangs [29–30 long]. Pedipalp [188–193 long] unmodified. Trochanter [26–29 long; 28–32 wide]. Femur [50–54 long; 35–37 wide]. Genu [39–41 long; 30–33 wide]. Tibia [51–52 long; 19–22 wide]. Tarsus [19–20(–21) long; 9–11 wide].

**Figure 30.**
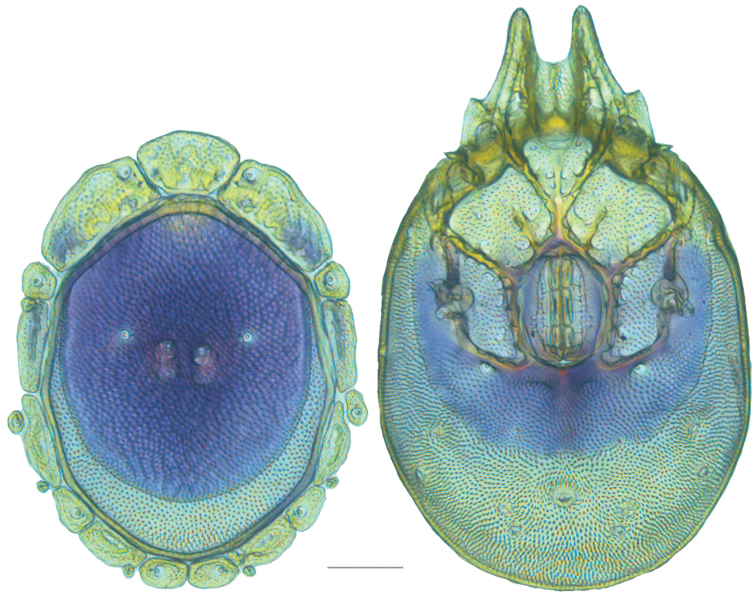
*Testudacarus dawkinsi* female: (**Left**) dorsum; (**Right**) venter. Scale: 100 µm.

Dorsum (Fig. 30) — [615–640 long; 475–501 wide] round to ovoid. Dorsal plate [497–528 long; 402–421 wide]. Primary sclerotization [421–453 long] violet to blue. Dorso-glandularia-4 [136–171 apart] lateral to [23–48] and around the anterior tips of muscle scars. Platelets violet to blue or colorless. Anterio-medial platelet [132–153 long; 80–102 wide] colorless rounded trapezoid noticeably smaller than anterio-lateral platelets. Anterio-lateral platelets [170–179 long; 81–91 wide] with violet to blue restricted to posterior half or third of the platelet. Lateral platelets as follows: lateral-1 [52–56 long; 44–49 wide]; lateral-2 [117–138 long; 31–46 wide]; lateral-3 [29–46 long; 20–26 wide]; lateral-4 [95–129 long; 33–38 wide]; lateral-5 [57–68 long; 32–36 wide]; lateral-6 [79–99 long; 32–43 wide]; lateral-7 [62–76 long; 32–39 wide].

Venter (Fig. 30) — [790–798; 510–534 wide] round to ovoid, fully sclerotized, and with anterior area of primary sclerotization [620–654 long] and posterior area of secondary sclerotization, violet to blue. Gnathosomal bay [77–84 dorsal length; 148–152 ventral length; 51–66 wide]. Coxal field [473–495 long; 330–368 wide]. Coxa-I [250–266 long; 100–114 midlength]. Coxa-II + III [119–125 distance to top of coxa-II; 188–195 distance to top of coxa-III; 350–370 distance to bottom of coxa-III; 229–245 total length]. Coxa-IV [325–343 distance to top; 144–155 total length]. Genital field [329–343 distance to top; 490–501 distance to bottom; 150–162 total length; 122–137 width; 181–194 distance from gnathosomal bay; 69–90 distance from coxa-I; 191–208 distance to excretory pore; 293–304 distance to caudad]. Distance to excretory pore [682–707].

Legs — violet to blue. Total leg and podomere lengths as follows: Leg-I [487–500 total; trochanter 57–63; basifemur 84–86; telofemur 67–73; genu 90–94; tibia 94–99; tarsus 87–94]. Leg-II [532–548 total; trochanter 58–65; basifemur 84–89; telofemur 67–72; genu 94–99; tibia 107–113; tarsus 110–116]. Leg-III [599–629 total; trochanter 63–68; basifemur 88–97; telofemur 73–76; genu 107–117; tibia 128–138; tarsus 134–140]. Leg-IV [830–861 total; trochanter 83–104; basifemur 113–130; telofemur 119–130; genu 156–164; tibia 172–181; tarsus 164–175].

**Male (n=9)** similar to female except for sexually dimorphic characters previously discussed and with following specifications.

Gnathosoma — Subcapitulum [143–156 ventral length; 89–97 dorsal length; 81–91 tall]. Chelicerae [113–129 long]. Fangs [26–30 long]. Pedipalp [180–195 long]. Trochanter [24–30 long; 26–29 wide]. Femur [50–53 long; 33–36 wide]. Genu [38–43 long; width 27–29 wide]. Tibia [47–52 long; 19–22 wide]. Tarsus [17–20 long; 9–10 wide].

**Figure 31.**
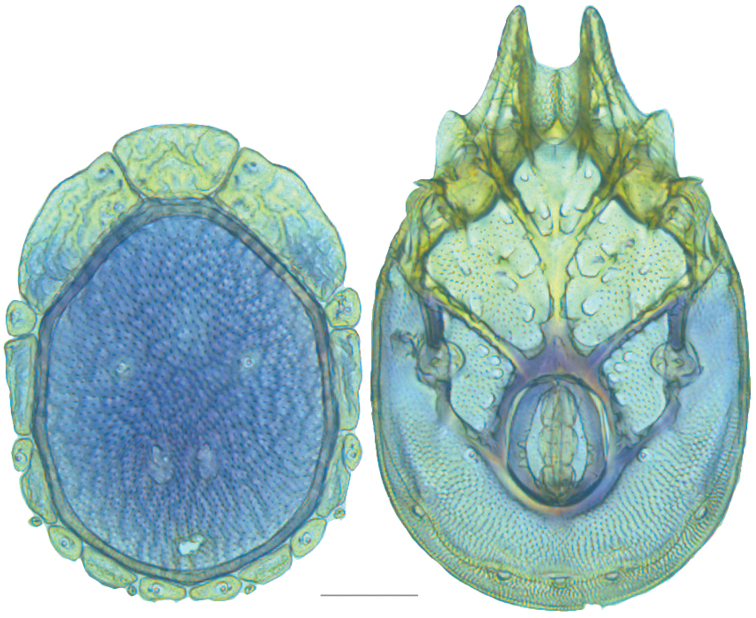
*Testudacarus dawkinsi* male: (**Left**) dorsum; (**Right**) venter. Scale: 100 µm.

Dorsum (Fig. 31) — [491–540 long; 368–421 wide]. Dorsal plate [401–443 long; 322–364 wide]. Dorso-glandularia-4 [101–143 apart] lateral to [6–35] and far anterior to [43–81] muscle scars. Anterio-medial platelet [121–144 long; 73–83 wide]. Anterio-lateral platelets [156–173 long; 74–80 wide]. Lateral platelets as follows: lateral-1 [37–46 long; 33–43 wide]; lateral-2 [95–115 long; 30–43 wide]; lateral-3 [33–48 long; 19–23 wide]; lateral-4 [75–95 long; 27–32 wide]; lateral-5 [40–50 long; 22–28 wide]; lateral-6 [58–69 long; 24–33 wide]; lateral-7 [42–56 long; 23–27 wide].

Venter (Fig. 31) — [618–683 long; 395–454 wide]. Primary sclerotization [583–641 long]. Gnathosomal bay [59–78 dorsal length; 139–153 ventral length; 50–61 wide]. Coxal field [434–484 long; 305–329 wide]. Coxa-I [227–256 long; 86–106 midlength]. Coxa-II + III [106–122 distance to top of coxa-II; 171–194 distance to top of coxa-III; 346–388 distance to bottom of coxa-III; 240–269 total length]. Coxa-IV [303–334 length to top; 130–154 total length]. Genital field [367–419 distance to top; 502–556 distance to bottom; 135–146 total length; 108–120 width; 224–267 distance from gnathosomal bay; 135–171 distance from coxa-I; 70–93 distance to excretory pore; 105–134 distance to caudad]. Genital skeleton [170–173 long; 85–105 wide]. Distance to excretory pore [583–641].

Legs — total leg and podomere lengths as follows: Leg-I [452–497 total; trochanter 52–63; basifemur 78–87; telofemur 63–72; genu 84–92; tibia 89–99; tarsus 84–93]. Leg-II [486–519 total; trochanter 53–61; basifemur 77–85; telofemur 59–70; genu 87–91; tibia 102–107; tarsus 101–112]. Leg-III [551–588 total; trochanter 54–60; basifemur 81–90; telofemur 66–73; genu 100–107; tibia 118–129; tarsus 126–134]. Leg-IV [752–796 total; trochanter 85–94; basifemur 99–120; telofemur 107–117; genu 143–146; tibia 158–163; tarsus 148–158].

#### Distribution.

Eastern United States east of the Mississippi River, with southern limits in Florida.

#### Etymology.

Specific epithet *dawkinsi* after Clinton Richard Dawkins, the English evolutionary biologist and writer. Dawkins has proven repeatedly that one can change the world as a biologist by day and keep going as a free-thinker by night.

### *Testudacarus americanus* complex

**Complex diagnosis.** These mites lack the four-segmented pedipalp of the *Debsacarus oribatoides*-like mites, the elongate body of the *Testudacarus elongatus*-like mites, and with the exception of *Testudacarus rollerae*, are much larger (female and male dorsal length usually more than 700 and 600 µm, respectively) than mites of the *Testudacarus minimus* and *Testudacarus hitchensi* complexes. *Testudacarus rollerae* have a larger (>140 µm) anterio-medial platelet that is more than or nearly twice as wide as long, while *Testudacarus minimus*-like mites have a smaller (<140 µm), more rounded anterio-medial platelet. These mites are present in western North America within and west of the Rocky Mountains, have very light to no coloration, have a large rectangular anterio-medial platelet, and comprise the following species: *Testudacarus kirkwoodae*, *Testudacarus americanus*, *Testudacarus hyporhynchus*, *Testudacarus smithi*, and *Testudacarus rollerae*.

**Figure 32.**
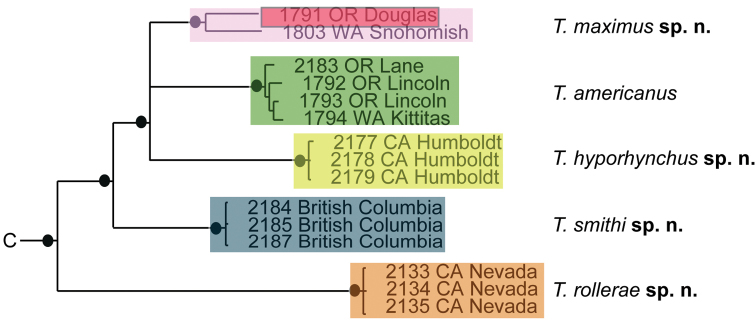
*Testudacarus americanus* complex molecular phylogeny: 28S and COI Bayesian analysis showing strong support for five distinct clades (●: >95% posterior probability); excluding pink clade, colored clades exhibit <1.3% divergence in COI within and >9% divergence between; pink exhibits 4.5% variation within; red specimen is a suspected species based on genetic data, but specimen is teneral and too badly damaged to diagnose; continuation of (**C**) lineage from Fig. 6.

**Remarks.** Molecular data show strong support for five distinct clades (Fig. 32). Four clades exhibit less than 1.3% COI intraclade divergence, and all five clades exhibit greater than 9% divergence between clades. The fifth clade (pink in Fig. 32) exhibits 4.5% divergence within. However, only two specimens of this clade are available. One is teneral and badly damaged and therefore provides no characters for morphological diagnoses. More specimens should be collected and analyzed. Otherwise, all five clades have diagnostic morphological features that further warrant species designations.

### Testudacarus americanus

Taxon classificationAnimaliaTrombidiformesTorrenticolidae

Marshall, 1943

http://zoobank.org/FE0E6228-D8AA-4063-A139-1C4A963454EB

Testudacarus americanus : Marshall 1943 : 320; Bergstrom 1953 : 160; Mitchell 1954 : 40; Imamura 1955 : 182, 188; Viets 1956 : 255; Habeeb 1959a: 21; Habeeb 1959b: 6; Crowell 1961 : 329; Mitchell 1962 : 42; Lundblad 1967 : 418; Habeeb 1967 : 1, 4; Habeeb 1969 : 2; Young 1969 : 373, 376–377, 380–381, 383–384, 386; Cook 1974 : 578–579; Habeeb 1974a : 1; Imamura 1976 : 283, 284; Smith 1982 : 901, 922–923, 981–985; Viets 1987 : 724–725; Smith and Cook 1991 : 582; Cramer 1992 : 14; Smith et al. 2001 : 625; Guo and Jin 2005 : 72; Walter et al. 2009 : 353; Smith et al. 2010 : 566; Smith et al. 2011 : 262.
Testudacarus american galloi : Habeeb 1969: 2

#### Type series.

**Holotype (1♀): California, USA**: 1♀ from Santa Cruz County, Waddell Creek, 29-30 June 1933, by PR Needham, RM330008

#### Other material examined.

**Other (9♀, 8♂): Oregon, USA**: 1♀ and 1♂ from Lincoln County, Siuslaw National Forest, Lord Creek, (44°14'24.00"N, 123°46'11.00"W), 8 August 2013, by JC O’Neill and WA Nelson, JNOW13-0808-002; 3♀ and 4♂ from Lane County, Cape Perpetua, Cape Perpetua Campground (44°16'51.00"N, 124°5'38.00"W), 15 September 2004, by IM Smith, IMS040077; 1♀ and 1♂ from Lane County, Rock Creek, Rock Creek Campground off Route 101 between Heceta Head and Yachats (44°11'6.00"N, 124°6'34.00"W), 14 September 2004, by IM Smith, IMS040076; 1♀ from Lane County, Cape Creek, Cape Perpetua, Cape Perpetua Campground (44°16'51.00"N, 124°5'38.00"W), 24 June 2010, by IM Smith, IMS100083; 1♀ and 1♂ from Curry County, Port Orford, beside road from Humbug Mountain State Park to McGribble Campground (Forest Road 5002) 5.3 km from Route 101 (42°42'11.00"N, 124°23'54.00"W), 25 June 1976, by IM Smith, IMS760161; 1♀ from Curry County, Port Orford, beside road from Humbug Mountain State Pk to McGribble Campground (Forest Road 5002) 4.6 km from Route 101 (42°42'3.00"N, 124°24'21.00"W), 17 June 2010, by IM Smith , IMS100070; 1♀ from Curry County, Siskiyou National Forest, North Fork of Foster Creek, beside Road #33 between Powers and Agness (42°39'N, 124°4'W), 2 July 1983, IMS 830019; **Washington, USA**: 1♂ from Kittitas County, Wenatchee National Forest, Squawk Creek, (47°16'51.00"N, 120°41'53.00"W), 31 July 2013, by JC O’Neill, WA Nelson, JNOW13-0731-002.

#### Type deposition.

Holotype (1♀) deposited at CNC.

#### Diagnosis.

Resembling most *Testudacarus smithi*, these mites differ by shape, color, and several other characters. Most notably, *Testudacarus americanus* are elliptical and colorless to peach and have a small cheliceral fang (<33 µm) while *Testudacarus smithi* are rounded and are grey to colorless with large cheliceral fangs (>40 µm).

#### Redescription.

**Female (n=10)** with characteristics of the genus with following specifications.

Gnathosoma — Subcapitulum [180–199 ventral length; 108–121 dorsal length; 110–124 tall] ovoid with short rostrum and colorless. Chelicerae [148–173 long] unmodified with lightly curved fangs [30–33 long]. Pedipalp [209–236 long] unmodified. Trochanter [29–37 long; 31–33 wide]. Femur [54–59 long; 38–45 wide]. Genu [47–60 long; 32–36 wide]. Tibia [53–59 long; 21–24 wide]. Tarsus [19–23 long; 9–13 wide].

**Figure 33.**
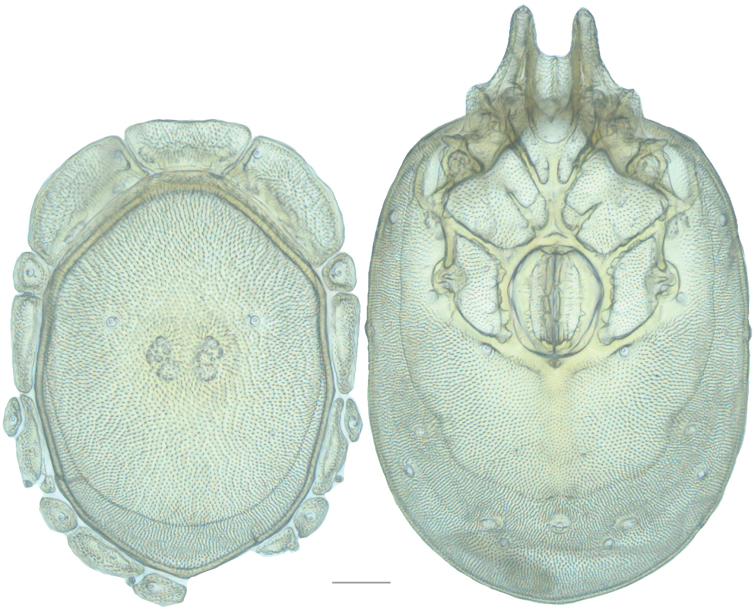
*Testudacarus americanus* female: (**Left**) dorsum; (**Right**) venter. Scale: 100 µm.

Dorsum (Fig. 33) — [826–890 long; 570–688 wide] ovoid to oblong and colorless with a peach tint. Dorsal plate [696–765 long; 497–561 wide]. Primary sclerotization [626–710 long]. Dorso-glandularia-4 [221–260 apart] anterior to [0–35] and lateral to [50–67] muscle scars. Anterio-medial platelet [198–241 long; 85–105 wide] broad, thin, very slightly rounded trapezoid similar in size to anterio-lateral platelets. Anterio-lateral platelets [219–253 long; 103–130 wide]. Lateral platelets as follows: lateral-1 [562–78 long; 48–59 wide]; lateral-2 [175–191 long; 41–59 wide]; lateral-3 [50–81 long; 22–45 wide]; lateral-4 [137–180 long; 40–56 wide]; lateral-5 [62–78 long; 40–55 wide]; lateral-6 [140–153 long; 39–56 wide]; lateral-7 [76–102 long; 36–52 wide].

Venter (Fig. 33) — [973–1095 long; 620–731 wide] ovoid to oblong and colorless. Primary sclerotization [828–934 long]. Gnathosomal bay [83–100 dorsal length; 171–196 ventral length; 67–82 wide]. Coxal field [555–615 long; 393–478 wide] noticeably small in relation to the venter when compared to other Testudacarines. Coxa-I [279–326 long; 106–141 midlength]. Coxa-II + III [128–145 distance to top of coxa-II; 211–256 distance to top of coxa-III; 391–444 distance to bottom of coxa-III; 263–304 total length]. Coxa-IV [368–427 distance to top; 170–198 total length]. Genital field [375–438 distance to top; 570–641 distance to bottom; 188–203 total length; 157–170 width; 203–259 distance from gnathosomal bay; 94–118 distance from coxa-I; 282–325 distance to excretory pore; 403–472 distance to caudad]. Eggs [182–200 long; 1–2 eggs]. Distance to excretory pore [880–964].

Legs — colorless. Total leg and podomere lengths as follows: Leg-I [542–604 total; trochanter 68–75; basifemur 91–108; telofemur 75–83; genu 95–111; tibia 108–122; tarsus 101–112]. Leg-II [548–611 total; trochanter 63–72; basifemur 95–108; telofemur 70–83; genu 93–103; tibia 107–123; tarsus 110–127]. Leg-III [595–683 total; trochanter 66–73; basifemur 99–108; telofemur 76–84; genu 103–124; tibia 127–150; tarsus 124–153]. Leg-IV [854–987 total; trochanter 94–112; basifemur 125–152; telofemur 134–154; genu 170–192; tibia 171–216; tarsus 154–181].

**Male (n=8)** similar to female except for sexually dimorphic characters previously discussed and with following specifications.

Gnathosoma — Subcapitulum [164–178 ventral length; 97–109 dorsal length; 96–114 tall]. Chelicerae [132–152 long]. Fangs [28–33 long]. Pedipalp [202–222 long]. Trochanter [28–33 long; 28–33 wide]. Femur [50–58 long; 38–42 wide]. Genu [50–55 long; width 30–35 wide]. Tibia [53–58 long; 20–22 wide]. Tarsus [18–23 long; 11–13 wide].

**Figure 34.**
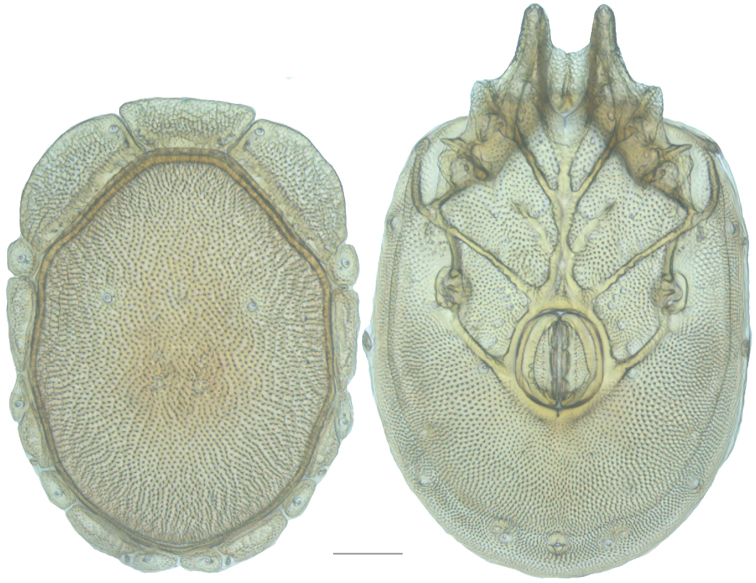
*Testudacarus americanus* male: (**Left**) dorsum; (**Right**) venter. Scale: 100 µm.

Dorsum (Fig. 34) — [678–755 long; 475–534 wide]. Dorsal plate [573–645 long; 405–463 wide]. Dorso-glandularia-4 [180–208 apart] lateral to [40–58] and well anterior to [53–90] muscle scars. Anterio-medial platelet [194–220 long; 82–103 wide]. Anterio-lateral platelets [198–227 long; 101–120 wide] without noticeable bump. Lateral platelets as follows: lateral-1 [52–62 long; 35–48 wide]; lateral-2 [125–164 long; 30–45 wide]; lateral-3 [47–68 long; 21–31 wide]; lateral-4 [92–120 long; 31–41 wide]; lateral-5 [55–68 long; 20–39 wide]; lateral-6 [87–125 long; 25–43 wide]; lateral-7 [47–63 long; 26–38 wide].

Venter (Fig. 34) — [840–893 long; 516–605 wide]. Primary sclerotization [763–841 long]. Gnathosomal bay [76–93 dorsal length; 150–174 ventral length; 64–89 wide]. Coxal field [520–594 long; 361–402 wide]. Coxa-I [276–291 long; 112–126 midlength]. Coxa-II + III [112–130 distance to top of coxa-II; 190–226 distance to top of coxa-III; 413–452 distance to bottom of coxa-III; 298–324 total length]. Coxa-IV [366–395 length to top; 150–203 total length]. Genital field [448–492 distance to top; 593–638 distance to bottom; 130–159 total length; 120–138 width; 292–323 distance from gnathosomal bay; 168–200 distance from coxa-I; 181–201 distance to excretory pore; 236–280 distance to caudad]. Genital skeleton [163–178 long; 80–88 wide]. Distance to excretory pore [780–833].

Legs — total leg and podomere lengths as follows: Leg-I [501–560 total; trochanter 57–64; basifemur 85–99; telofemur 70–80; genu 90–102; tibia 101–113; tarsus 95–108]. Leg-II [508–567 total; trochanter 58–67; basifemur 88–96; telofemur 67–74; genu 83–99; tibia 101–117; tarsus 105–119]. Leg-III [554–615 total; trochanter 59–63; basifemur 83–98; telofemur 70–76; genu 97–115; tibia 117–138; tarsus 119–132]. Leg-IV [526–882 total; trochanter 79–103; basifemur 116–130; telofemur 121–135; genu 157–175; tibia 171–197; tarsus 149–166].

#### Distribution.

Western North America within and west of the Rocky Mountains. California (Marshall 1943), Wyoming (Bergstrom 1953), Colorado (Young 1969), Vancouver Island (Smith 1982), Mexico State (Cramer 1992).

#### Remarks.

Having examined the type material, we suggest that *Testudacarus americanus galloi* Habeeb, 1969 is simply a teneral *Testudacarus americanus* that Habeeb confused for an “a-typical” *Testudacarus americanus*.

### Testudacarus kirkwoodae

Taxon classificationAnimaliaTrombidiformesTorrenticolidae

O’Neill & Dowling sp. n.

http://zoobank.org/B46CE9B8-65BC-4F12-B8C2-791C23CDB4BA

#### Type series.

**Holotype (1♀) Oregon, USA**: 1♀ from Douglas County, Rouge River National Forest, Muir Creek, (43°2'53.00"N, 122°21'4.00"W), 12 August 2013, by JC O’Neill and WA Nelson, JNOW13-0812-004 (Specimen 141885 – DNA#1791); **Other examined but not measured (1♀): Washington, USA**: 1♀ from Snohomish County, Mount Baker National Forest, tributary of South Fork of Sauk River, (48°1'40.00"N, 121°26'24.00"W), 28 July 2013, JC O’Neill and WA Nelson, JNOW13-0728-003

#### Type deposition.

Holotype (1♀) deposited at CNC.

#### Diagnosis.

These are the largest known testudacarines and also differ further from their complex in having a smaller, more rounded anterio-medial platelet.

#### Description.

**Female (n=1)** with characteristics of genus with following specifications.

Gnathosoma — Subcapitulum [245 ventral length; 133 dorsal length; 130 tall] ovoid with short rostrum. Chelicerae [200 long] unmodified with lightly curved fangs [40 long]. Pedipalp [259 long] unmodified. Trochanter [38 long; 43 wide]. Femur [70 long; 50 wide]. Genu [63 long; 40 wide]. Tibia [63 long; 28 wide]. Tarsus [25 long; 15 wide].

**Figure 35.**
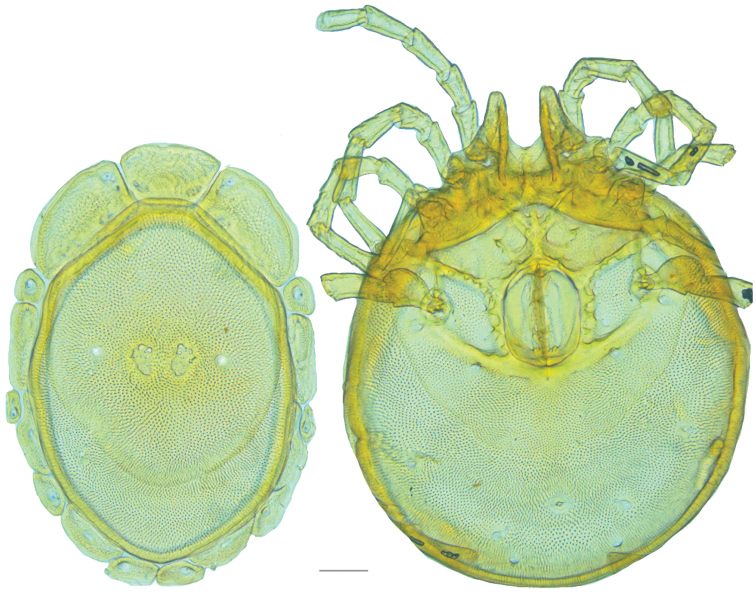
*Testudacarus kirkwoodae* female: (**Left**) dorsum; (**Right**) venter. Scale: 100 µm.

Dorsum (Fig. 35) — [918 long; 645 wide] ovoid to oblong. Dorsal plate [758 long; 566 wide]. Primary sclerotization [603 long] light pink to colorless. Dorso-glandularia-4 [232 apart] lateral to [63] and around muscle scar midline. Platelets colorless. Anterio-medial platelet [201 long; 123 wide]. Anterio-lateral platelets [237 long; 134 wide]. Lateral platelets as follows: lateral-1 [65 long; 55 wide]; lateral-2 [173 long; 46 wide]; lateral-3 [67 long; 24 wide]; lateral-4 [173 long; 46 wide]; lateral-5 [91 long; 47 wide]; lateral-6 [149 long; 54 wide]; lateral-7 [93 long; 46 wide].

Venter (Fig. 35) — [1045 long; 853 wide] round to ovoid and colorless. Primary sclerotization [752 long]. Gnathosomal bay [123 dorsal length; 151 ventral length; 69 wide]. Coxal field [551 long; 484 wide] proportionally small compared to venter. Coxa-I [294 long; 144 midlength]. Coxa-II + III [114 distance to top of coxa-II; 202 distance to top of coxa-III; 393 distance to bottom of coxa-III; 279 total length]. Coxa-IV [376 distance to top; 175 total length]. Genital field [373 distance to top; 578 distance to bottom; 205 total length; 69 width; 222 distance from gnathosomal bay; 78 distance from coxa-I; 272 distance to excretory pore; 467 distance to caudad]. Distance to excretory pore [850].

Legs — colorless. Total leg and podomere lengths as follows: Leg-I [620 total; trochanter 70; basifemur 119; telofemur 89; genu 101; tibia 119; tarsus 121]. Leg-II [681 total; trochanter 77; basifemur 113; telofemur 90; genu 117; tibia 145; tarsus 140]. Leg-III [756 total; trochanter 70; basifemur 116; telofemur 93; genu 144; tibia 165; tarsus 167]. Leg-IV [1081 total; trochanter 136; basifemur 148; telofemur 159; genu 207; tibia 224; tarsus 208].

**Male (n=0)** unknown.

#### Etymology.

Specific epithet *kirkwoodae* after my (JCO) mother’s maiden name. It was her bringing me to the leafcutter ant exhibit at our local science center that helped me become interested in biology as a child, and her endless support and advice that helped me finish my education.

#### Remarks.

As it is likely that this species represents a cryptic species complex (with 4.5% COI divergence between the two available specimens), measurements were included from only one specimen (the other highlighted in red in Fig. 32 was excluded). This was done so measurements would remain useful if more species were diagnosed in the future. Measurments were also not included from the other specimen because it is teneral and badly damaged and would therefore prove poor for any description. While more than a single specimen is certainly desired for new descriptions, the included specimen has unique morphological characters such as its large size (it is larger than any other testudacarine, including species from Asia), and has strong support as a unique clade using COI. Therefore, this single specimen is unique enough that we are comfortable describing it.

#### Distribution.

Only one specimen know from Douglas County, Oregon.

### Testudacarus hyporhynchus

Taxon classificationAnimaliaTrombidiformesTorrenticolidae

O’Neill & Dowling sp. n.

http://zoobank.org/371AF7BE-204F-4CA7-8CE4-34EF7647B751

#### Type series.

**Holotype (1♀): California, USA**: 1♀ Humboldt County, Willow Creek, Willow Creek Campground off Rt. 299 (40°54'17.00"N, 123°42'21.00"W), 14 June 2010, by IM Smith, IMS100065 (Specimen 146762 – DNA#2177); **Paratype (1♀, 2♂): California, USA**: (allotype) 1♂ Humboldt County, Willow Creek, Willow Creek Campground off Rt. 299 (40°54'17.00"N, 123°42'21.00"W), 14 June 2010, by IM Smith, IMS100065 (Specimen 146763 – DNA#2178); 1♂ Humboldt County, Willow Creek, Willow Creek Campground off Rt. 299 (40°54'17.00"N, 123°42'21.00"W), 14 June 2010, by IM Smith, IMS100065; **Oregon, USA**: 1♀ from Curry County, Port Orford, beside Elk River Road 9.0 km east of Elk River Fish Hatchery (42°42'22.00"N, 124°20'28.00"W), 22 June 2010, by IM Smith, IMS100080.

#### Type deposition.

Holotype (1♀) and allotype (1♂) deposited at CNC; two paratypes (1♀, 1♂) at ACUA.

#### Diagnosis.

These mites differ from the rest of the complex in having a dorsally “covered” gnathosomal bay (short doral gnathosomal bay length) and an elongate gnathosoma with a long rostrum that exteneds below the gnathosoma ventral surface.

#### Description.

**Female (n=2)** with characteristics of genus with following specifications.

**Figure 36.**
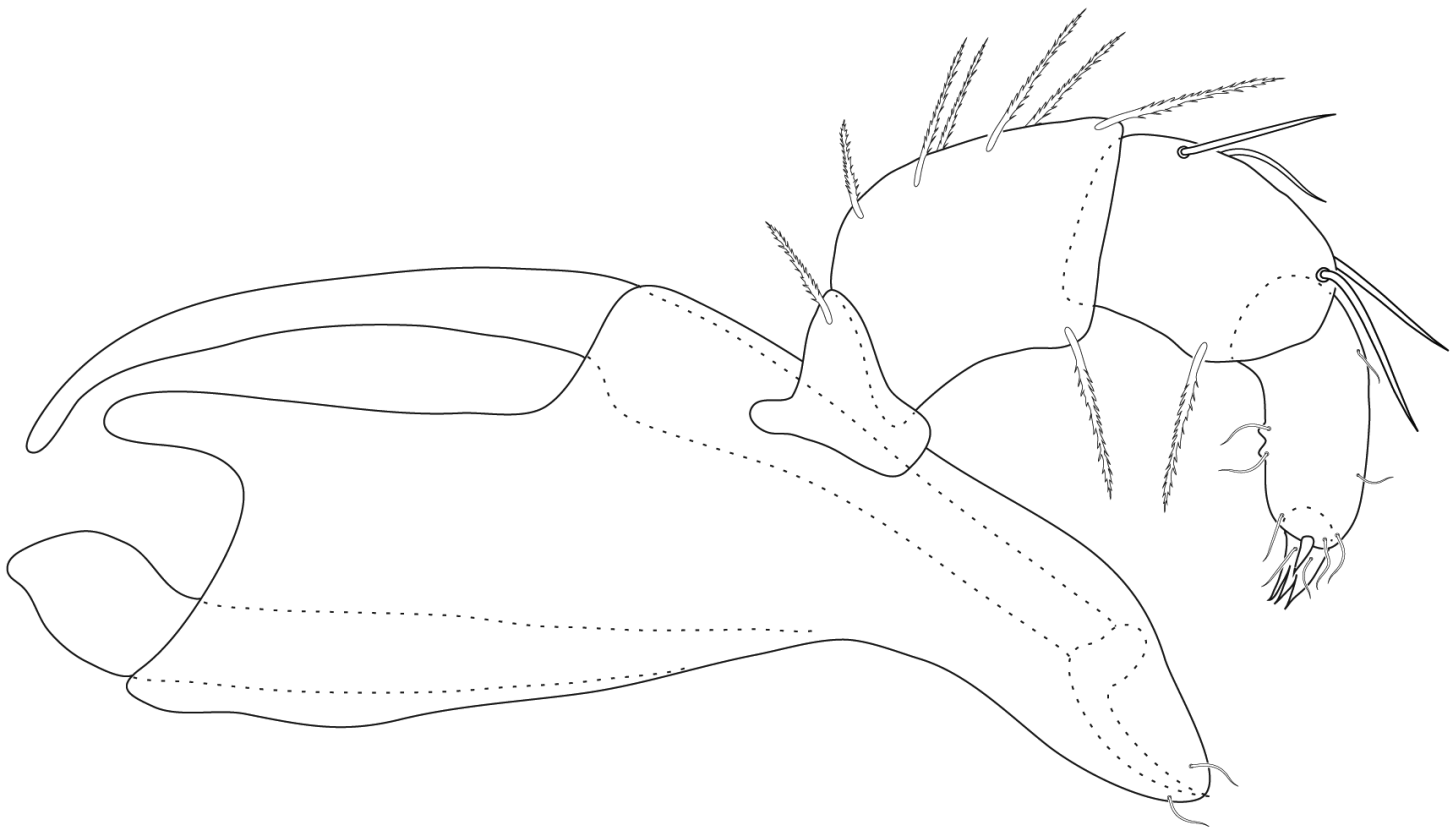
*Testudacarus hyporhynchus* **sp. n.** gnathosoma (generalized).

Gnathosoma (Fig. 36) — Subcapitulum [244–250 ventral length; 150–155 dorsal length; 89–97 tall] elongate with long rostrum extending below ventral surface; colorless. Chelicerae [210–220 long] unmodified with lightly curved fangs [32–36 long]. Pedipalp [194–203 long] unmodified. Trochanter [24–25 long; 38–40 wide]. Femur [53–56 long; 42–43 wide]. Genu [44–45 long; 34–35 wide]. Tibia [50–54 long; 22–23 wide]. Tarsus [21–22 long; 9–10 wide].

**Figure 37.**
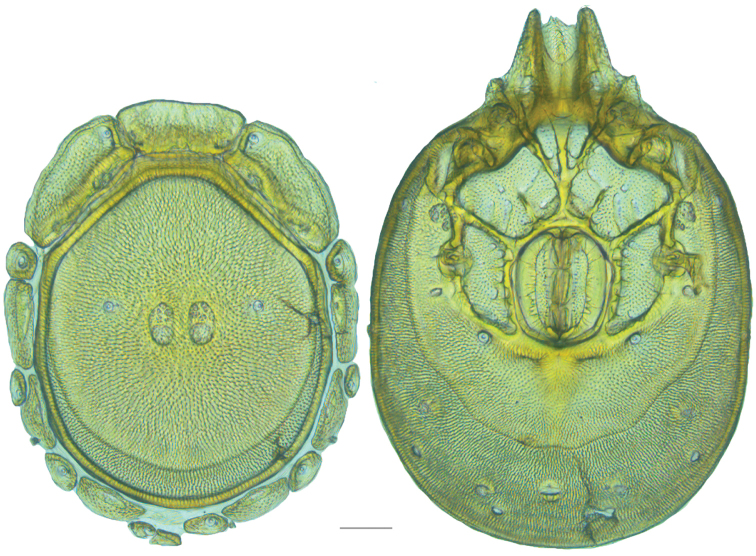
*Testudacarus hyporhynchus* female: (**Left**) dorsum; (**Right**) venter. Scale: 100 µm.

Dorsum (Fig. 37) — [768–849 long; 634–668 wide] round to ovoid and mostly colorless. Dorsal plate [570–578 long; 645–693 wide]. Primary sclerotization [540–583 long] colorless to light pink. Dorso-glandularia-4 [230–252 apart] lateral to [44–61] and just anterior to [0–27] muscle scars. Platelets colorless. Anterio-medial platelet [230–252 long; 103–116 wide]. Anterio-lateral platelets [229–246 long; 117–123 wide]. Lateral platelets as follows: lateral-1 [66–83 long; 56–59 wide]; lateral-2 [156–188 long; 47–51 wide]; lateral-3 [60–84 long; 29–30 wide]; lateral-4 [139–150 long; 35–45 wide]; lateral-5 [79–93 long; 41–42 wide]; lateral-6 [129–144 long; 39–44 wide]; lateral-7 [76–88 long; 35–41 wide].

Venter (Fig. 37) — [1001–1049 long; 700–728 wide] round to ovoid and colorless. Primary sclerotization [814–824 long]. Gnathosomal bay [14–20 dorsal length; 101–102 ventral length; 67–70 wide] short dorsally giving a “covered” appearance and ventrally ending anterior to leg-I insertion. Coxal field [590–616 long; 404–433 wide]. Coxa-I [298–316 long; 196–216 midlength]. Coxa-II + III [127–143 distance to top of coxa-II; 216–264 distance to top of coxa-III; 427–446 distance to bottom of coxa-III; 299–303 total length]. Coxa-IV [399–434 distance to top; 182–191 total length]. Genital field [410–424 distance to top; 627–642 distance to bottom; 217–218 total length; 174–189 width; 308–323 distance from gnathosomal bay; 108–112 distance from coxa-I; 252–270 distance to excretory pore; 374–407 distance to caudad] large. Distance to excretory pore [879–912].

Legs — colorless. Total leg and podomere lengths as follows: Leg-I [566–594 total; trochanter 68–74; basifemur 104–106; telofemur 81–85; genu 108–112; tibia 109–117; tarsus 95–102]. Leg-II [614–645 total; trochanter 75–78; basifemur 102–108; telofemur 77–79; genu 111–116; tibia 124–130; tarsus 125–135]. Leg-III [714–753 total; trochanter 74–79; basifemur 109–119; telofemur 88–94; genu 136–139; tibia 154–160; tarsus 152–161]. Leg-IV [952–961 total; trochanter 105–109; basifemur 142–144; telofemur 138–139; genu 191–192; tibia 199–201; tarsus 175–178].

**Male (n=2)** similar to female except for sexually dimorphic characters previously discussed and with following specifications.

Gnathosoma (Fig. 36) — Subcapitulum [222–239 ventral length; 136–151 dorsal length; 85–89 tall]. Chelicerae [203–218 long]. Fangs [33–34 long]. Pedipalp [195–200 long]. Trochanter [24–25 long; 36–38 wide]. Femur [55–58 long; 42–46 wide]. Genu [45–46 long; width 34–35 wide]. Tibia [50–54 long; 21–23 wide]. Tarsus [17–20 long; 8–9 wide].

**Figure 38.**
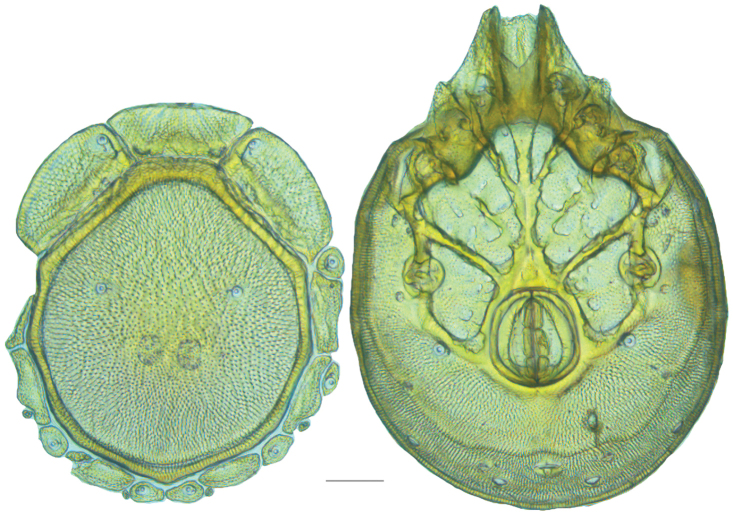
*Testudacarus hyporhynchus* male: (**Left**) dorsum; (**Right**) venter. Scale: 100 µm.

Dorsum (Fig. 38) — [667–712 long; 548–582 wide]. Dorsal plate [546–616 long; 470–471 wide]. Dorso-glandularia-4 [192–212 apart] lateral to and well anterior to muscle scars [65–67 anterior to; 40–55 lateral to]. Anterio-medial platelet [244–249 long; 91–100 wide]. Anterio-lateral platelets [225–229 long; 111–116 wide]. Lateral platelets as follows: lateral-1 [49–61 long; 43–60 wide]; lateral-2 [135–152 long; 47–50 wide]; lateral-3 [53–60 long; 24–25 wide]; lateral-4 [135–144 long; 37–41 wide]; lateral-5 [60–75 long; 35–37 wide]; lateral-6 [101–105 long; 32–37 wide]; lateral-7 [59–63 long; 31–33 wide].

Venter (Fig. 38) — [835–898 long; 625–626 wide]. Primary sclerotization [750–791 long]. Gnathosomal bay [16–20 dorsal length; 99–101 ventral length; 67–68 wide]. Coxal field [553–576 long; 403–408 wide]. Coxa-I [296–318 long; 195–218 midlength]. Coxa-II + III [116–127 distance to top of coxa-II; 212–234 distance to top of coxa-III; 430–473 distance to bottom of coxa-III; 314–346 total length]. Coxa-IV [377–413 length to top; 163–176 total length]. Genital field [473–506 distance to top; 636–681 distance to bottom; 163–175 total length; 149–151 width; 372–407 distance from gnathosomal bay; 177–188 distance from coxa-I; 129–134 distance to excretory pore; 200–217 distance to caudad]. Genital skeleton [180–187 long; 117–122 wide]. Distance to excretory pore [765–815].

Legs — total leg and podomere lengths as follows: Leg-I [593–606 total; trochanter 73–79; basifemur 111–114; telofemur 78–83; genu 107–111; tibia 113–118; tarsus 105–106]. Leg-II [635–645 total; trochanter 74–79; basifemur 102–104; telofemur 84–85; genu 115–120; tibia 129–132; tarsus 124–130]. Leg-III [724–726 total; trochanter 77–78; basifemur 109–116; telofemur 87–91; genu 136–137; tibia 155–156; tarsus 152–155]. Leg-IV [905–964 total; trochanter 107–118; basifemur 139–140; telofemur 129–142; genu 183–191; tibia 179–198; tarsus 167–176].

#### Etymology.

Specific epithet *hyporhynchus* (*hypo*-, G. under; *rhynchus*, G. snout) refers to the long rostrum that extends below the ventral surface of the gnathosoma.

#### Distribution.

Humbolt County, California and Curry County, Oregon.

### Testudacarus smithi

Taxon classificationAnimaliaTrombidiformesTorrenticolidae

O’Neill & Dowling sp. n.

http://zoobank.org/870A0931-9FDB-4293-B6E9-2AB5BAA733C5

#### Type series.

**Holotype (1♀): British Columbia, Canada**: 1♀ from Vancouver Island, spring run, Lake Cowichan, beside North Shore Road 1.7 km north of town (48°49'29.00"N, 124°4'2.00"W), 1 July 2010, by IM Smith, IMS100091 (Specimen 146769 – DNA#2184); **Paratypes (10♀, 12♂): British Columbia, Canada**: (allotype) 1♂ from Vancouver Island, spring run, Lake Cowichan, beside North Shore Road 1.7 km north of town (48°49'29.00"N, 124°4'2.00"W), 1 July 2010, by IM Smith, IMS100091 (Specimen 146770 – DNA#2185); 2♂ from Vancouver Island, spring run, Lake Cowichan, beside North Shore Road 1.7 km north of town (48°49'29.00"N, 124°4'2.00"W), 1 July 2010, by IM Smith, IMS100091; 3♀ and 2♂ from Vancouver Island, Lake Cowichan, spring run, beside North Shore Road 1.7 km north of town (48°49'29.00"N, 124°4'13.00"W), 11 June 1979, by IM Smith, IMS790013A; 3♀ and 3♂ from Vancouver Island, Lake Cowichan, spring run, beside South Shore Road 2.3 km north of town (48°48'25.00"N, 124°5'13.00"W), 7 July 1976, by IM Smith, IMS760194; 3♀ and 2♂ from Vancouver Island, Port Alberni, beside road to Mount Arrowsmith Ski Area 11.6 km from Highway 4 (49°12'50.00"N, 124°36'18.00"W), 19 September 2004, by IM Smith, IMS040084A; 1♀ and 1♂ from Vancouver Island, Lake Cowichan, spring run, beside South Shore Road 2.3 km north of town (48°48'25.00"N, 124°5'13.00"W), 31 July 1979, by IM Smith, IMS790056; 1♂ from Vancouver Island, Lake Cowichan, spring run, beside South Shore Road 2.3 km north of town (48°48'25.00"N, 124°5'13.00"W), 26 July 1985, by IM Smith, IMS850122A.

#### Type deposition.

Holotype (1♀), allotype (1♂), and eight paratypes (4♀, 4♂) deposited at CNC; thirteen paratypes (6♀, 7♂) at ACUA.

#### Diagnosis.

Resembling most *Testudacarus americanus*, these mites differ by shape, color, and several other characters. Most notably, *Testudacarus americanus* are elliptical and colorless to peach and have a small cheliceral fang (<33 µm) while *Testudacarus smithi* are rounded and are grey to colorless with large cheliceral fangs (>40 µm).

#### Description.

**Female (n=10)** with characteristics of genus with following specifications.

Gnathosoma — Subcapitulum [217–245 ventral length; 137–145 dorsal length; 125–152 tall] elliptical to ovoid with short rostrum. Chelicerae [190–205 long] unmodified with lightly curved fangs [40–45 long]; fangs characteristically large. Pedipalp [250–272 long] unmodified. Trochanter [37–45 long; 39–46 wide]. Femur [72–80 long; 52–61 wide]. Genu [55–61 long; 41–49 wide]. Tibia [57–66 long; 23–26 wide]. Tarsus [21–26 long; 11–13 wide].

**Figure 39.**
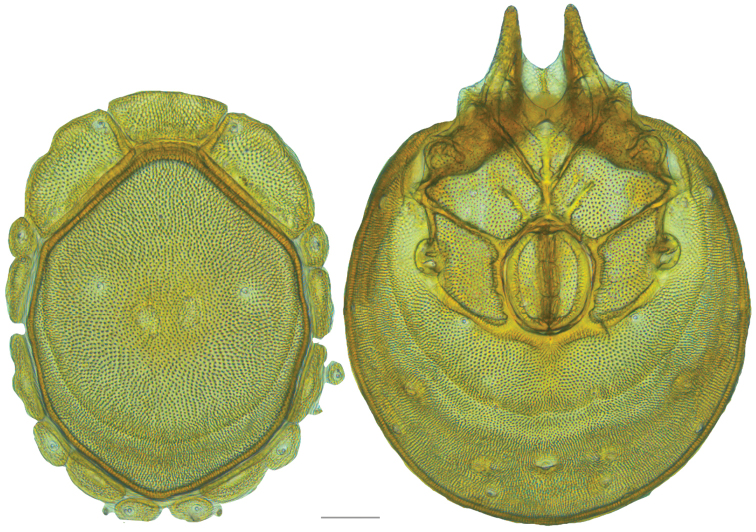
*Testudacarus smithi* female: (**Left**) dorsum; (**Right**) venter. Scale: 100 µm.

Dorsum (Fig. 39) — [790–864 long; 619–683 wide] round to ovoid. Dorsal plate [643–705 long; 500–549 wide]. Primary sclerotization [541–596 long] grey-violet. Dorso-glandularia-4 [215–246 apart] lateral to [45–72] and just anterior to [0–21] muscle scars. Platelets colorless. Anterio-medial platelet [200–233 long; 103–128 wide] large slightly rounded trapezoid approaching size of anterio-lateral platelets. Anterio-lateral platelets [230–266 long; 125–149 wide]. Lateral platelets as follows: lateral-1 [69–82 long; 47–67 wide]; lateral-2 [127–154 long; 38–54 wide]; lateral-3 [37–65 long; 22–40 wide]; lateral-4 [156–185 long; 31–54 wide]; lateral-5 [80–102 long; 40–60 wide]; lateral-6 [106–158 long; 35–60 wide]; lateral-7 [73–103 long; 36–55 wide].

Venter (Fig. 39) — [955–1047; 671–816 wide] round to ovoid and colorless. Primary sclerotization [742–814 long]. Gnathosomal bay [87–130 dorsal length; 164–216 ventral length; 78–105 wide]. Coxal field [578–641 long; 421–488 wide]. Coxa-I [302–361 long; 133–157 midlength]. Coxa-II + III [130–165 distance to top of coxa-II; 223–262 distance to top of coxa-III; 416–476 distance to bottom of coxa-III; 284–317 total length]. Coxa-IV [384–435 distance to top; 184–222 total length]. Genital field [390-455 distance to top; 595–657 distance to bottom; 201–217 total length; 173–184 width; 222–251 distance from gnathosomal bay; 84–102 distance from coxa-I; 229–298 distance to excretory pore; 332–429 distance to caudad]. Eggs [185–200 long; 1–3 eggs]. Distance to excretory pore [853–924].

Legs — colorless. Total leg and podomere lengths as follows: Leg-I [624–660 total; trochanter 74–81; basifemur 110–121; telofemur 84–95; genu 117–125; tibia 123–130; tarsus 108–116]. Leg-II [658–718 total; trochanter 75–87; basifemur 111–123; telofemur 83–95; genu 115–130; tibia 133–149; tarsus 133–145]. Leg-III [755–820 total; trochanter 78–89; basifemur 112–131; telofemur 88–102; genu 139–155; tibia 162–184; tarsus 165–182]. Leg-IV [1017–1058 total; trochanter 117–132; basifemur 139–150; telofemur 140–153; genu 197–210; tibia 212–228; tarsus 194–205].

**Male (n=12)** similar to female except for sexually dimorphic characters previously discussed and with following specifications.

Gnathosoma — Subcapitulum [205–232 ventral length; 125–145 dorsal length; 124–133 tall]. Chelicerae [178–202 long]. Fangs [39–42 long]. Pedipalp [250–279 long]. Trochanter [37–42 long; 38–43 wide]. Femur [71–81 long; 52–60 wide]. Genu [55–65 long; width 40–47 wide]. Tibia [59–69 long; 23–25 wide]. Tarsus [21–27 long; 11–15 wide].

**Figure 40.**
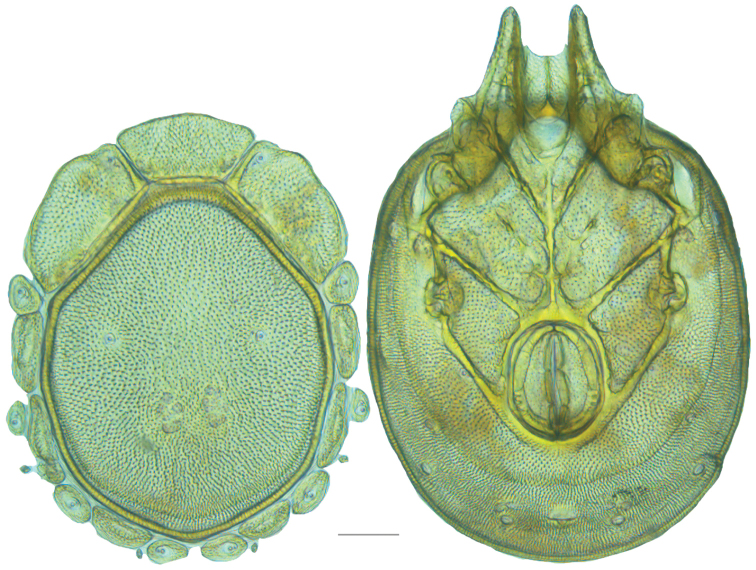
*Testudacarus smithi* male: (**Left**) dorsum; (**Right**) venter. Scale: 100 µm.

Dorsum (Fig. 40) — [682–790 long; 523–626 wide]. Dorsal plate [567–670 long; 440–521 wide] with minute amount of secondary sclerotization. Dorso-glandularia-4 [198–261 apart] roughly equal distance anterior to and lateral to muscle scars [39–92 anterior to; 42–72 lateral to]. Anterio-medial platelet [194–221 long; 99–108 wide]. Anterio–lateral [216–249 long; 116–135 wide]. Lateral platelets as follows: lateral-1 [62–79 long; 45–57 wide]; lateral-2 [114–150 long; 37–52 wide]; lateral-3 [30–67 long; 21–33 wide]; lateral-4 [123–160 long; 30–48 wide]; lateral-5 [67–90 long; 32–48 wide]; lateral-6 [91–121 long; 33–47 wide]; lateral-7 [50–79 long; 29–44 wide].

Venter (Fig. 40) — [868–974; 575–730 wide]. Primary sclerotization [728–820 long]. Gnathosomal bay [79–126 dorsal length; 183–206 ventral length; 72–101 wide]. Coxal field [582–657 long; 394–468 wide]. Coxa-I [320–346 long; 131–146 midlength]. Coxa-II + III [132–158 distance to top of coxa-II; 233–264 distance to top of coxa-III; 470–515 distance to bottom of coxa-III; 327–370 total length]. Coxa-IV [378–436 length to top; 182–234 total length]. Genital field [490–545 distance to top; 675–742 distance to bottom; 185–210 total length; 150–166 width; 307–340 distance from gnathosomal bay; 170–200 distance from coxa-I; 90–137 distance to excretory pore; 177–244 distance to caudad]. Genital skeleton [245–272 long; 125–152 wide]. Distance to excretory pore [790–874]. Excretory pore characteristically well separated from line of secondary sclerotization.

Legs — total leg and podomere lengths as follows: Leg-I [617–679 total; trochanter 73–80; basifemur 110–122; telofemur 84–97; genu 116–129; tibia 118–138; tarsus 107–123]. Leg-II [664–743 total; trochanter 74–84; basifemur 110–125; telofemur 85–103; genu 118–137; tibia 134–157; tarsus 131–148]. Leg-III [753–841 total; trochanter 76–83; basifemur 111–133; telofemur 89–106; genu 138–158; tibia 163–186; tarsus 155–186]. Leg-IV [952–1098 total; trochanter 111–131; basifemur 136–154; telofemur 139–161; genu 189–217; tibia 189–238; tarsus 176–210].

#### Etymology.

Specific epithet *smithi* after Dr Ian Smith, the Canadian water mite researcher who collected the specimens needed for this description. Dr Smith has advanced water mite research in North America more than anyone else and without him, this work would have been impossible.

#### Distribution.

British Columbia, Canada.

### Testudacarus rollerae

Taxon classificationAnimaliaTrombidiformesTorrenticolidae

O’Neill & Dowling sp. n.

http://zoobank.org/74EBEF64-B599-45A8-B79E-6A07D1DA05A8

#### Type series.

**Holotype (1♀): California, USA**: 1♀ from Nevada County, Tahoe National Forest, Bear River, at Sierra Discovery day use area upstream from bridge (39°18'35.00"N, 120°39'56.00"W), 26 August 2013, by JR Fisher, JRF13-0826-001 (Specimen 146725 – DNA# 2135); **Paratypes (2♀, 2♂): California, USA**: (allotype) 1♂ (allotype)from Nevada County, Tahoe National Forest, Bear River, at Sierra Discovery day use area upstream from bridge (39°18'35.00"N, 120°39'56.00"W), 26 August 2013, by JR Fisher, JRF13-0826-001 (Specimen 146724 – DNA# 2134); 1♀ from Nevada County, Tahoe National Forest, Bear River, at Sierra Discovery day use area upstream from bridge (39°18'35.00"N, 120°39'56.00"W), 26 August 2013, by JR Fisher, JRF13-0826-001; 1♀ and 1♂ from Calaveras County, Calaveras Big Trees State Park, Big Trees River, (38°16'N, 120°16'W), 12 June 1976, by IM Smith, IMS760099; 1♂ from Mendocino County, Navarro River, Paul M. Dimmick Recreation Area beside Route 128 (39°`10'N, 123°38'W), 29 September 1993, by IM Smith, IMS9300026A.

#### Type deposition.

Holotype (1♀) and allotype (1♂) deposited at CNC; four paratypes (2♀, 2♂) at ACUA.

#### Diagnosis.

These mites are smaller and more colorful than other species in the complex and therefore resemble most the *Testudacarus minimus*-like mites; however, mites of the *Testudacarus minimus* complex are even smaller and have a smaller (<140 µm, and far less than twice as wide as long), more rounded anterio-medial platelet, while these mites have a larger (>140 µm, and more than or nearly twice as wide as long) more rectangular anterio-medial platelet.

#### Description.

**Female (n=3)** with characteristics of genus with following specifications.

Gnathosoma — Subcapitulum [176–188 ventral length; 105–107 dorsal length; 100–103 tall] elliptical ovoid with short rostrum and colorless. Chelicerae [145–152 long] unmodified with lightly curved fangs [28–30 long]. Pedipalp [202–212 long] unmodified. Trochanter [30–31 long; 28–30 wide]. Femur [56–58 long; 40–41 wide]. Genu [43–47 long; 33–35 wide]. Tibia [52–55 long; 20–23 wide]. Tarsus [20–22 long; 9–10 wide].

**Figure 41.**
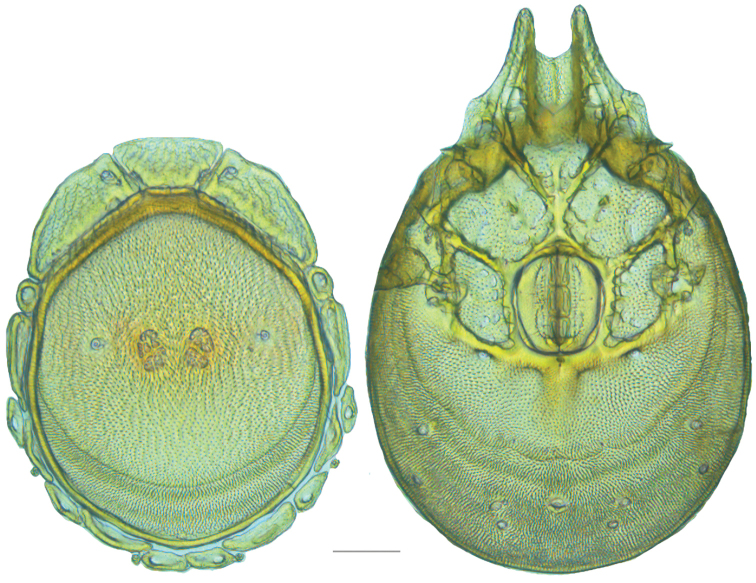
*Testudacarus rollerae* female: (**Left**) dorsum; (**Right**) venter. Scale: 100 µm.

Dorsum (Fig. 41) — [625–680 long; 483–550 wide] ovoid and mostly colorless. Dorsal plate [526–568 long; 410–475 wide]. Primary sclerotization [431–473 long] light pink to colorless. Dorso-glandularia-4 [192–246 apart] lateral to [45–58] and around muscle scar midline. Platelets colorless. Anterio-medial platelet [153–164 long; 83–93 wide] large trapezoid with nearly straight anterior margin. Anterio-lateral platelets [181–211 long; 88–91 wide]. Lateral platelets as follows: lateral-1 [46–52 long; 38–40 wide]; lateral-2 [132–148 long; 33–39 wide]; lateral-3 [50–69 long; 19–26 wide]; lateral-4 [107–112 long; 22–29 wide]; lateral-5 [61–86 long; 27–32 wide]; lateral-6 [112–128 long; 25–34 wide]; lateral-7 [31–77 long; 23–33 wide].

Venter (Fig. 41) — [786–884 long; 548–644 wide] round to ovoid and colorless. Primary sclerotization [624–709 long]. Gnathosomal bay [81–96 dorsal length; 154–164 ventral length; 56–60 wide]. Coxal field [478–532 long; 335–394 wide]. Coxa-I [261–290 long; 106–126 midlength]. Coxa-II + III [122–137 distance to top of coxa-II; 198–224 distance to top of coxa-III; 363–395 distance to bottom of coxa-III; 237–257 total length]. Coxa-IV [346–385 distance to top; 132–148 total length]. Genital field [347–374 distance to top; 500–539 distance to bottom; 153–165 total length; 130–139 width; 193–210 distance from gnathosomal bay; 79–87 distance from coxa-I; 199–231 distance to excretory pore; 286–345 distance to caudad]. Eggs [165–178 long; 1 egg]. Distance to excretory pore [699–770].

Legs — colorless. Total leg and podomere lengths as follows: Leg-I [503–542 total; trochanter 65–68; basifemur 88–93; telofemur 70–81; genu 93–103; tibia 96–102; tarsus 89–97]. Leg-II [510–577 total; trochanter 52–74; basifemur 82–94; telofemur 66–78; genu 94–102; tibia 103–118; tarsus 106–112]. Leg-III [610–657 total; trochanter 64–71; basifemur 92–99; telofemur 73–82; genu 110–122; tibia 127–146; tarsus 137–141]. Leg-IV [843–914 total; trochanter 96–101; basifemur 116–125; telofemur 117–133; genu 166–185; tibia 181–195; tarsus 168–178].

**Male (n=3)** similar to female except for sexually dimorphic characters previously discussed and with following specifications.

Gnathosoma — Subcapitulum [160–170 ventral length; 98–109 dorsal length; 81–92 tall]. Chelicerae [132–140 long]. Fangs [27–29 long]. Pedipalp [184–190 long]. Trochanter [23–26 long; 29–30 wide]. Femur [50–51 long; 36–38 wide]. Genu [41–42 long; width 29–30 wide]. Tibia [49–52 long; 19–20 wide]. Tarsus [17–21 long; 8–9 wide].

**Figure 42.**
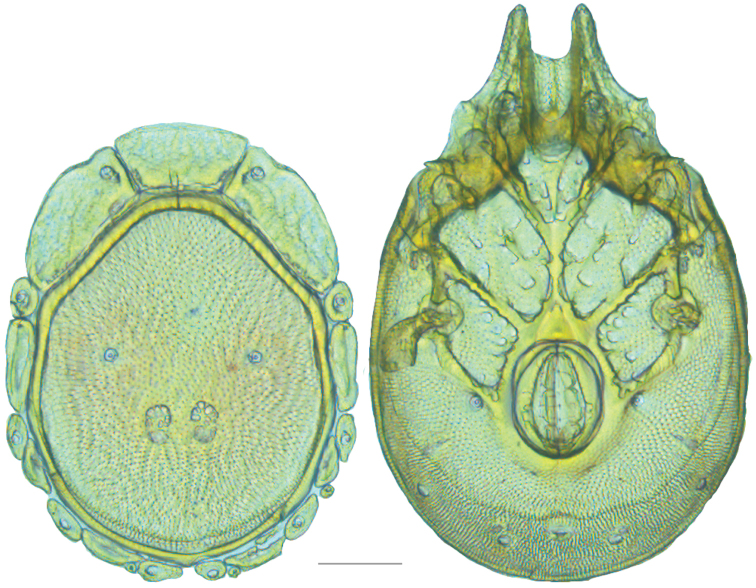
*Testudacarus rollerae* male: (**Left**) dorsum; (**Right**) venter. Scale: 100 µm.

Dorsum (Fig. 42) — [540–585 long; 412–433 wide]. Dorsal plate [444–487 long; 355–384 wide] with minute secondary sclerotization. Dorso-glandularia-4 [151–167 apart] roughly equal in distance anterior to [32–53] and lateral to [31–43] muscle scars. Anterio-medial platelet [151–158 long; 80–85 wide]. Anterio-lateral platelets [180–188 long; 84–91 wide]. Lateral platelets as follows: lateral-1 [44–49 long; 33–38 wide]; lateral-2 [109–114 long; 31–35 wide]; lateral-3 [50–63 long; 19–22 wide]; lateral-4 [75–91 long; 18–29 wide]; lateral-5 [60–65 long; 22–29 wide]; lateral-6 [66–82 long; 20–33 wide]; lateral-7 [52–61 long; 22–30 wide].

Venter (Fig. 42) — [698–740 long; 453–544 wide]. Primary sclerotization [623–655 long]. Gnathosomal bay [71–80 dorsal length; 138–147 ventral length; 54–60 wide]. Coxal field [475–484 long; 325–374 wide]. Coxa-I [253–263 long; 111–117 midlength]. Coxa-II + III [118–131 distance to top of coxa-II; 185–198 distance to top of coxa-III; 382–396 distance to bottom of coxa-III; 251–274 total length]. Coxa-IV [337–356 length to top; 127–139 total length]. Genital field [406–426 distance to top; 547–570 distance to bottom; 142–146 total length; 114–123 width; 263–280 distance from gnathosomal bay; 152–164 distance from coxa-I; 75–91 distance to excretory pore; 148–176 distance to caudad]. Genital skeleton [190–215 long; 110–112 wide]. Distance to excretory pore [623–655].

Legs — total leg and podomere lengths as follows: Leg-I [472 total; trochanter 59–60; basifemur 83–91; telofemur 66–71; genu 88–91; tibia 91–97; tarsus 83–88]. Leg-II [496–515 total; trochanter 61–66; basifemur 84–87; telofemur 65–69; genu 85–97; tibia 96–108; tarsus 102–107]. Leg-III [554–593 total; trochanter 62–69; basifemur 84–89; telofemur 65–74; genu 100–110; tibia 117–126; tarsus 125–136]. Leg-IV [784–822 total; trochanter 80–95; basifemur 109–116; telofemur 114–115; genu 155–162; tibia 164–174; tarsus 155–162].

#### Etymology.

Specific epithet *rollerae* after Elizabeth Ashley Roller, my (JCO) life partner.

#### Distribution.

Reported from only two counties (Mendocino and Nevada) in California.

### *Testudacarus elongatus* complex

**Complex diagnosis.** Unlike all other Testudacarinae, members of this complex have an elongate idiosoma. In contrast to most *Testudacarus minimus*- and *Testudacarus hitchensi*-like mites, these mites are colorless and much larger (female and male dorsal length greater than 700 and 600 µm, respectively). These mites are found in western North America west of the Rocky Mountains. This complex comprises three species: *Testudacarus elongatus*, *Testudacarus oblongatus*, and *Testudacarus rectangulatus*.

**Remarks.**

**Figure 43.**
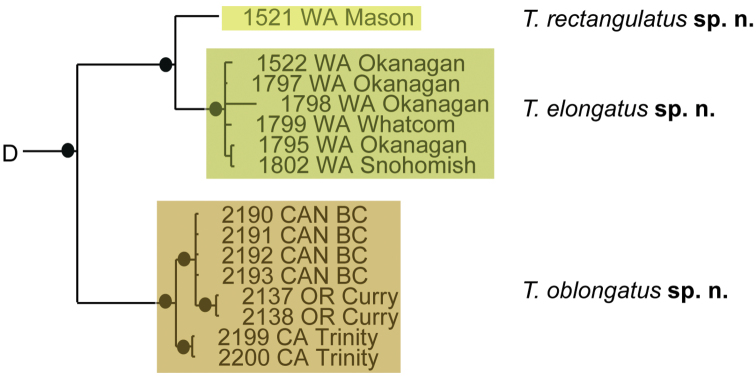
*Testudacarus elongatus* complex molecular phylogeny: 28S and COI Bayesian analysis showing strong support at least three distinct clades (●: >95% posterior probability); colored clades exhibit <2.4% divergence in COI within and >3.3% divergence between; divergence of the two basal clades >9%; continuation of (**D**) lineage from Fig. 43.

Combined molecular, distributional, and morphological data support three distinct clades within the *Testudacarus elongatus* complex (Fig. 43). All three clades exhibit less than 2.4% COI intra-clade divergence and greater than 3.3% divergence between clades. Intra-clade divergence of 2.4%, as seen with *Testudacarus oblongatus*, is not unexpected for a species exhibiting a large geographic range (British Columbia to California); however, a percent difference as high as 3.3% between two close localities (Mason and Snohomish County, between *Testudacarus rectangulatus* and *Testudacarus elongatus*) suggests separate species. Interestingly, COI divergence of more than 9% between the two sister clades (*Testudacarus oblongatus* and *Testudacarus elongatus*/*Testudacarus rectangulatus*) does not seem to produce high amounts of morphological diversity within this complex. Therefore, the morphological varation and geograhic varation found between *Testudacarus elongatus* and *Testudacarus rectangulatus* provide enough evidence for us to hypothesize two species, even if one is from a single specimen. Potentially, there is a coastal species (*Testudacarus oblongatus*), a species within and east of the cascade (*Testudacarus elongatus*), and a species from the Olympic Mountains (*Testudacarus rectangulatus*).

#### Testudacarus elongatus

Taxon classificationAnimaliaTrombidiformesTorrenticolidae

O’Neill & Dowling sp. n.

http://zoobank.org/4A0801F6-C137-48DD-B16C-333E68F81A56

##### Type series.

**Holotype (1♀): Washington, USA**: 1♀ from Okanogan County, Okanogan National Forest, Early Winters Creek, (48°35'55.00"N, 120°35'20.00"W), 29 July 2013, by JC O’Neill and WA Nelson, JNOW13-0729-004 (Specimen 138495 – DNA#1522); **Paratypes (7♀, 4♂): Washington, USA**: (allotype) 1♂ from Okanogan County, Okanogan National Forest, Early Winters Creek, (48°35'55.00"N, 120°35'20.00"W), 29 July 2013, by JC O’Neill and WA Nelson, JNOW13-0729-004 (Specimen 141889 – DNA#1797); 1♂ from Whatcom County, Mount Baker National Forest, Porcupine Creek, (48°31'51.00"N, 120°44'42.00"W), 29 July 2013, by JC O’Neill and WA Nelson, JNOW13-0729-003; 2♀ and 2♂ from Okanogan County, Okanogan National Forest, Early Winters Creek, (48°35'55.00"N, 120°35'20.00"W), 29 July 2013, by JC O’Neill and WA Nelson, JNOW13-0729-004; 1♀ from Snohomish County, Mount Baker National Forest, tributary of South Fork of Sauk River, (48°1'40.00"N, 121°26'24.00"W), 28 July 2013, JC O’Neill and WA Nelson, JNOW13-0728-003; 1♀ from Okanogan County, Okanogan National Forest, North Fork of Twentymile Creek, (48°43'7.00"N, 119°56'14.00"W), 29 July 2013, by JC O’Neill and WA Nelson, JNOW13-0729-007; 3♀ from Okanagan County, North Fork of Salmon Creek, (48°37'48.00"N, 119°48'52.00"W), 29 July 2013, by JC O’Neill and WA Nelson, JNOW13-0729-008.

##### Type deposition.

Holotype (1♀), allotype (1♂), and four paratypes (3♀, 1♂) deposited at CNC; six paratypes (4♀, 2♂) at ACUA.

##### Diagnosis.

Since morphological variation is limited, a combination of morphology and distribution is best used to diagnose members of the complex. These mites occur in Washington within and east of the Cascade Mountains, while *Testudacarus rectangulatus* occur in the Olympic Mountains, and *Testudacarus oblongatus* occur along the western Coast of Washington, Oregon, California, and British Columbia. Additionally, both *Testudacarus rectangulatus* and these mites differ from *Testudacarus oblongatus* in having more robust lateral platelets; most notably, lateral-platelet-4 tends to be larger in these two species than *Testudacarus oblongatus*, and is in direct or near direct contact with lateral-platelet-2. Reversely, *Testudacarus oblongatus* generally have less robust platelets and a smaller lateral-platelet-4 that has a noticeable gap between it and lateral-platelet-2. Limited specimens were found of *Testudacarus elongatus* and *Testudacarus rectangulatus*, but *Testudacarus rectangulatus* appear to have leg and pedipalp measurements roughly 10% larger than *Testudacarus elongatus* even between individuals of similar idiosoma size. More data is needed to better diagnose these species.

##### Description.

**Female (n=8)** with characteristics of genus with following specifications.

Gnathosoma — Subcapitulum [174–175 ventral length; 110–118 dorsal length; 102–122 tall] ovoid with short rostrum. Chelicerae [140–163 long] unmodified with lightly curved fangs [33–37 long]. Pedipalp [221–248 long] unmodified. Trochanter [28–38 long; 32–36 wide]. Femur [60–64 long; 45–50 wide]. Genu [51–59 long; 37–43 wide]. Tibia [61–69 long; 24–28 wide]. Tarsus [20–25 long; 10–12 wide].

**Figure 44.**
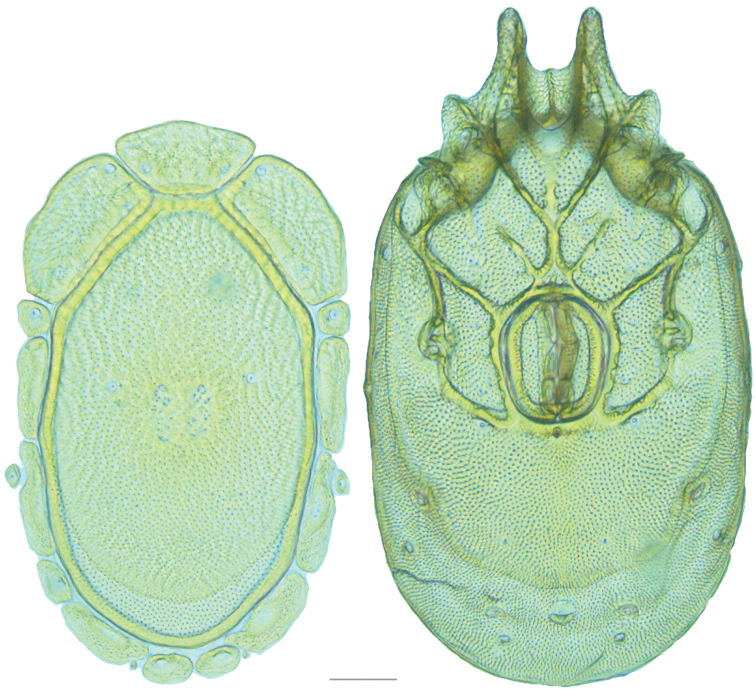
*Testudacarus elongatus* female: (**Left**) dorsum; (**Right**) venter. Scale: 100 µm.

Dorsum (Fig. 44) — [765–861 long; 507–563 wide] oblong and colorless. Dorsal plate [661–723 long; 407–470 wide]. Primary sclerotization [599–650 long]. Dorso-glandularia-4 [163–216 apart] lateral to [23–68] and around the anterior tips of muscle scars. Platelets colorless. Anterio-medial platelet [173–207 long; 101–117 wide] trapeziform to nearly triangular (posterior margin strongly shortened). Anterio-lateral platelets [199–224 long; 105–123 wide] near rectangular. Lateral platelets as follows: lateral-1 [55–66 long; 45–62 wide]; lateral-2 [137–173 long; 35–52 wide]; lateral-3 [23–48 long; 18–24 wide]; lateral-4 [166–188 long; 34–51 wide]; lateral-5 [46–70 long; 26–39 wide]; lateral-6 [118–144 long; 30–45 wide]; lateral-7 [66–78 long; 32–38 wide].

Venter (Fig. 44) — [947–1051; 536–682 wide] oblong. Primary sclerotization [798–880 long]. Gnathosomal bay [86–108 dorsal length; 138–178 ventral length; 63–95 wide]. Coxal field [185–198 long; 366–479 wide]. Coxa-I [260–307 long; 118–133 midlength]. Coxa-II + III [95–134 distance to top of coxa-II; 190–221 distance to top of coxa-III; 383–432 distance to bottom of coxa-III; 273–314 total length]. Coxa-IV [362–394 distance to top; 182–207 total length]. Genital field [383–419 distance to top; 568–614 distance to bottom; 185–198 total length; 140–166 width; 221–262 distance from gnathosomal bay; 102–132 distance from coxa-I; 265–298 distance to excretory pore; 377–443 distance to caudad]. Eggs [270 long; 1–2 eggs]. Distance to excretory pore [846–910].

Legs — colorless. Total leg and podomere lengths as follows: Leg-I [561–614 total; trochanter 63–66; basifemur 101–114; telofemur 79–93; genu 103–116; tibia 110–127; tarsus 96–109]. Leg-II [559–623 total; trochanter 56–65; basifemur 96–120; telofemur 75–88; genu 103–116; tibia 120–128; tarsus 107–120]. Leg-III [630–703 total; trochanter 60–80; basifemur 97–116; telofemur 79–96; genu 116–139; tibia 136–152; tarsus 129–140]. Leg-IV [863–920 total; trochanter 98–109; basifemur 126–140; telofemur 130–140; genu 172–189; tibia 183–194; tarsus 152–167].

**Male (n=4)** similar to female except for sexually dimorphic characters previously discussed and with following specifications.

Gnathosoma — Subcapitulum [148–160 ventral length; 98–108 dorsal length; 95–100 tall]. Chelicerae [135–140 long]. Fangs [30–31 long]. Pedipalp [208–215 long]. Trochanter [30–31 long; 28–33 wide]. Femur [53–60 long; 40–44 wide]. Genu [47–52 long; width 33–35 wide]. Tibia [55–61 long; 23–25 wide]. Tarsus [20–21 long; 10–12 wide].

**Figure 45.**
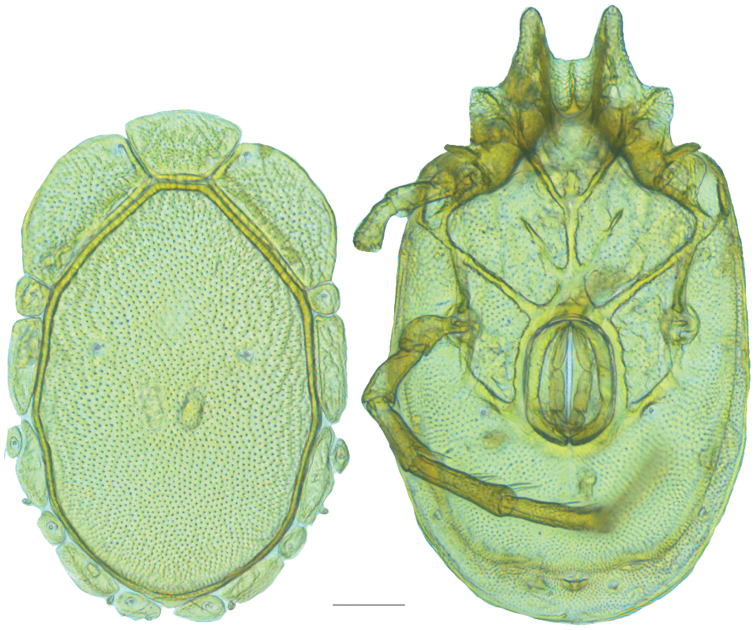
*Testudacarus elongatus* male: (**Left**) dorsum; (**Right**) venter. Scale: 100 µm.

Dorsum (Fig. 45) — [680–759 long; 426–480 wide]. Dorsal plate [564–647 long; 359–404 wide] occasionally with minute area of secondary sclerotization. Dorso-glandularia-4 [180–198 apart] roughly equal distance anterior to [31–60] and lateral to [50–63] muscle scars. Anterio-medial platelet [160–177 long; 98–104 wide]. Anterio-lateral platelets [189–217 long; 100–115 wide]. Lateral platelets as follows: lateral-1 [38–52 long; 38–47 wide]; lateral-2 [147–155 long; 39–46 wide]; lateral-3 [29–52 long; 15–22 wide]; lateral-4 [138–161 long; 35–43 wide]; lateral-5 [41–60 long; 28–32 wide]; lateral-6 [93–107 long; 30–42 wide]; lateral-7 [60–66 long; 26–38 wide].

Venter (Fig. 45) — [830–890 long; 497–578 wide]. Primary sclerotization [764–812 long]. Gnathosomal bay [72–85 dorsal length; 137–157 ventral length; 71–86 wide]. Coxal field [530–564 long; 372–390 wide]. Coxa-I [233–268 long; 96–112 midlength]. Coxa-II + III [95–114 distance to top of coxa-II; 180–201 distance to top of coxa-III; 376–418 distance to bottom of coxa-III; 281–311 total length]. Coxa-IV [333–368 length to top; 187–211 total length]. Genital field [392–436 distance to top; 562–615 distance to bottom; 169–179 total length; 120–128 width; 256–283 distance from gnathosomal bay; 159–173 distance from coxa-I; 170–220 distance to excretory pore; 244–299 distance to caudad]. Genital skeleton [220–238 long; 145 wide]. Distance to excretory pore [764–812].

Legs — total leg and podomere lengths as follows: Leg-I [531–558 total; trochanter 54–65; basifemur 95–99; telofemur 75–80; genu 98–104; tibia 105–111; tarsus 100–105]. Leg-II [549–572 total; trochanter 59–65; basifemur 93–101; telofemur 73–79; genu 98–105; tibia 112–120; tarsus 100–109]. Leg-III [577–610 total; trochanter 64–69; basifemur 93–105; telofemur 75–84; genu 106–119; tibia 122–135; tarsus 117–131]. Leg-IV [765–812 total; trochanter 91–100; basifemur 110–120; telofemur 115–121; genu 153–171; tibia 152–168; tarsus 140–152].

**Etymology.** Specific epithet *elongatus* (*elong*-, L. extend) refers to the elongate idiosoma of adults.

##### Distribution.

Within and east of the Cascade Mountains, Washington.

#### Testudacarus rectangulatus

Taxon classificationAnimaliaTrombidiformesTorrenticolidae

O’Neill & Dowling sp. n.

http://zoobank.org/A7CA6FCE-2D33-482B-AF90-7C93964F5D71

##### Type series.

**Holotype (1♂): Washington, USA**: 1♂ from Mason County, Olympic National Forest, Cabin Creek, by Hamma Hamma River (47°35'44.00"N, 123°7'39.00"W), 22 July 2013, by JC O’Neill and WA Nelson, JNOW13-0722-004 (Specimen 138494 – DNA#1521)

##### Type deposition.

Holotype (1♂) deposited at CNC

##### Diagnosis.

Since morphological variation is limited, a combination of morphology and distribution is best used to diagnosed members of the complex. These mites occur in the Olympic Mountains, while *Testudacarus elongatus* occur in Washington within and east of the Cascade Mountains, and *Testudacarus oblongatus* occur along the western Coast of Washington, Oregon, California, and British Columbia. Additionally, both *Testudacarus elongatus* and these mites differ from *Testudacarus oblongatus* in having more robust lateral platelets; most notably, lateral-platelet-4 tends to be larger in these two species than *Testudacarus oblongatus*, and is in direct or near direct contact with lateral-platelet-2. Reversely, *Testudacarus oblongatus* generally have less robust platelets and a smaller lateral-platelet-4 that has a noticeable gap between it and lateral-platelet-2. Limited specimens were found of *Testudacarus elongatus* and *Testudacarus rectangulatus*, but *Testudacarus rectangulatus* appear to have leg and pedipalp measurements roughly 10% larger than *Testudacarus elongatus* even between individuals of similar idiosoma size. More data is needed to better diagnose these species.

##### Description.

**Female (n=0)** unknown.

**Male (n=1)** with characteristics of genus with following specifications.

Gnathosoma — Subcapitulum [173 ventral length; 108 dorsal length; 105 tall] ovoid with short rostrum. Chelicerae [150 long] unmodified with lightly curved fangs [33-37 long]. Pedipalp [249 long] unmodified. Trochanter [35 long; 34 wide]. Femur [60 long; 48 wide]. Genu [56 long; 40 wide]. Tibia [75 long; 25 wide]. Tarsus [23 long; 12 wide].

**Figure 46.**
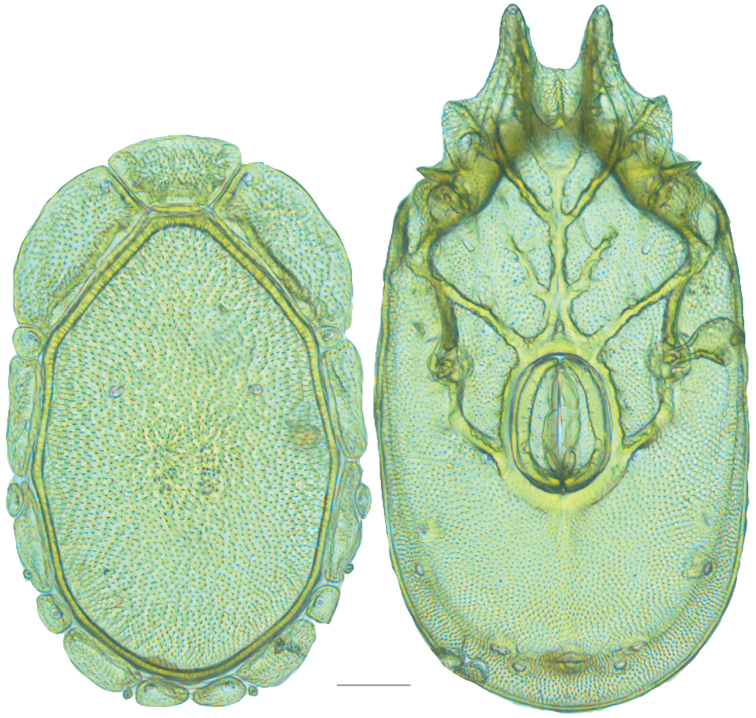
*Testudacarus rectangulatus* male: (**Left**) dorsum; (**Right**) venter. Scale: 100 µm.

Dorsum (Fig. 46) — [773 long; 495 wide] oblong and colorless. Dorsal plate [649 long; 413 wide]. Dorso-glandularia-4 [173 apart] lateral to [41] and anterior to [63] muscle scars. Platelets colorless. Anterio-medial platelet [183 long; 108 wide] trapeziform to nearly triangular (posterior margin strongly shortened). Anterio-lateral platelets [216 long; 114 wide] near rectangular. Lateral platelets as follows: lateral-1 [40 long; 45 wide]; lateral-2 [161 long; 41 wide]; lateral-3 [39 long; 23 wide]; lateral-4 [165 long; 40 wide]; lateral-5 [55 long; 34 wide]; lateral-6 [112 long; 49 wide]; lateral-7 [69 long; 37 wide].

Venter (Fig. 46) — [929 long; 492 wide] oblong. Primary sclerotization [855 long]. Gnathosomal bay [83 dorsal length; 162 ventral length; 89 wide]. Coxal field [577 long; 390 wide]. Coxa-I [278 long; 116 midlength]. Coxa-II + III [122 distance to top of coxa-II; 203 distance to top of coxa-III; 439 distance to bottom of coxa-III; 317 total length]. Coxa-IV [375 distance to top; 201 total length]. Genital field [461 distance to top; 647 distance to bottom; 186 total length; 133 width; 299 distance from gnathosomal bay; 183 distance from coxa-I; 208 distance to excretory pore; 282 distance to caudad]. Genital skeleton [250 long]. Distance to excretory pore [855].

Legs — colorless. Total leg and podomere lengths as follows: Leg-I [603 total; trochanter 61; basifemur 103; telofemur 89; genu 116; tibia 124; tarsus 108]. Leg-II [610 total; trochanter 63; basifemur 101; telofemur 85; genu 115; tibia 126; tarsus 120]. Leg-III [674 total; trochanter 63; basifemur 110; telofemur 86; genu 130; tibia 145; tarsus 137]. Leg-IV [870 total; trochanter 87; basifemur 123; telofemur 130; genu 179; tibia 189; tarsus 160].

##### Etymology.

Specific epithet *rectangulatus* (*rectangulum*, L. straight angle) refers to the boxy, elongate idiosoma of adults.

##### Distribution.

One specimen found in Mason County in the Olympic Mountains, Washington.

#### Testudacarus oblongatus

Taxon classificationAnimaliaTrombidiformesTorrenticolidae

O’Neill & Dowling sp. n.

http://zoobank.org/411D6BD4-3740-4FE0-A7BB-30733BF28851

##### Type series.

**Holotype (1♀): Oregon, USA**: 1♀ from Curry County, Siskiyou National Forest, confluence of tributary and Wheeler Creek, off NF 1205 (42°4'42.00"N, 124°8'53.00"W), by JR Fisher, JRF13-0814-004 (Specimen 146728 – DNA#2138); P**aratypes (11♀, 9♂): British Columbia, Canada**: (allotype) 1♂ from Vancouver Island, beside Harris Creek Mainline 5 km west of Old Hillcrest Gate (26 km west of Mesachie Lake) (48°40'7.00"N, 124°13'20.00"W), 3 July 2010, by IM Smith, IMS100095 (Specimen 146776 – DNA#2192); 2♀ and 1♂ from Vancouver Island, beside Harris Creek Mainline 5 km west of Old Hillcrest Gate (26 km west of Mesachie Lake) (48°40'7.00"N, 124°13'20.00"W), 3 July 2010, by IM Smith, IMS100095; 3♀ and 1♂ from Vancouver Island, beside Highway 4 16.6 km east of road to Ucluelet (Pacific Rim Road) (49°9'N, 125°54'W), 18-19 July 1979, by IM Smith, IMS790047; 1♀ and 3♂ from Bonanza Pass Walker Creek Picnic Area beside Highway 3 between Grand Forks and Castlegar (49°10'N, 118°5'W), 20 July 1988, by IM Smith, IMS880034; 1♂ from Vancouver Island, Honeymoon Bay Wildflower Reserve, (48°49'38.00"N, 124°12'10.00"W), 19 June 1979, by IM Smith, IMS790023A; 1♀ from Vancouver Island, beside Harris Creek Mainline 5 km west of Old Hillcrest Gate (26 km west of Mesachie Lake) (48°40'6.00"N, 124°13'19.00"W), 3 July 2010, by IM Smith, IMS100097; 2♀ from Vancouver Island, beside Harris Creek Mainline 5 km west of Old Hillcrest Gate (26 km west of Mesachie Lake) (48°40'6.00"N, 124°13'16.00"W), 10 July 1988, by IM Smith, IMS880007; **California, USA**: 1♂ from Monterey County, Los Padres National Forest, Lucia, beside Ferguson-Nacimiento Road 5.6 km east of Route 1 (36°0'3.00"N, 121°28'31.00"W), 3 June 2010, by IM Smith, IMS100048; 1♂ from Trinity County, Shasta-Trinity National Forest, beside Route 36 6.2 km west of Forest Glen Station Campground (40°22'57.00"N, 123°23'26.00"W), 11 June 2010, by IM Smith, IMS100061; 1♀ from Trinity County, Shasta-Trinity National Forest, beside Route 36 7 km west of Forest Glen Station Campground (40°23'5.00"N, 123°23'57.00"W), 11 June 2010, by IM Smith, IMS100062; **Oregon, USA**: 1♀ from Curry County, Siskiyou National Forest, confluence of tributary and Wheeler Creek, off NF 1205 (42°4'42.00"N, 124°8'53.00"W), by JR Fisher, JRF13-0814-004.

##### Type deposition.

Holotype (1♀), allotype (1♂), and ten paratypes (6♀, 4♂) deposited at CNC; nine paratypes (5♀, 4♂) at ACUA.

##### Diagnosis.

Since morphological variation is limited, a combination of morphology and distribution is best used to diagnosed members of the complex. These mites occur along the western coast of Washington, Oregon, California, and British Columbia, while *Testudacarus elongatus* occur in Washington within and east of the Cascade Mountains, and *Testudacarus rectangulatus* occur in the Olympic Mountains. These mites differ from others in the complex in having less robust platelets and a smaller lateral-platelet-4 that has a noticeable gap between it and lateral-platelet-2. Reversely lateral-platelet-4 tends to be larger in the other two species of the complex than *Testudacarus oblongatus*, and is in direct or near direct contact with lateral-platelet-2.

##### Description.

**Female (n=11)** with characteristics of genus with following specifications.

Gnathosoma — Subcapitulum [192–208 ventral length; 116–132 dorsal length; 120–134 tall] ovoid with short rostrum. Chelicerae [149–166 long] unmodified with lightly curved fangs [33–36 long]. Pedipalp [231–242 long] unmodified. Trochanter [31–36 long; 30–33 wide]. Femur [58–63 long; 45–49 wide]. Genu [53–59 long; 36–38 wide]. Tibia [64–69 long; 22–25 wide]. Tarsus [20–25 long; 11–12 wide].

**Figure 47.**
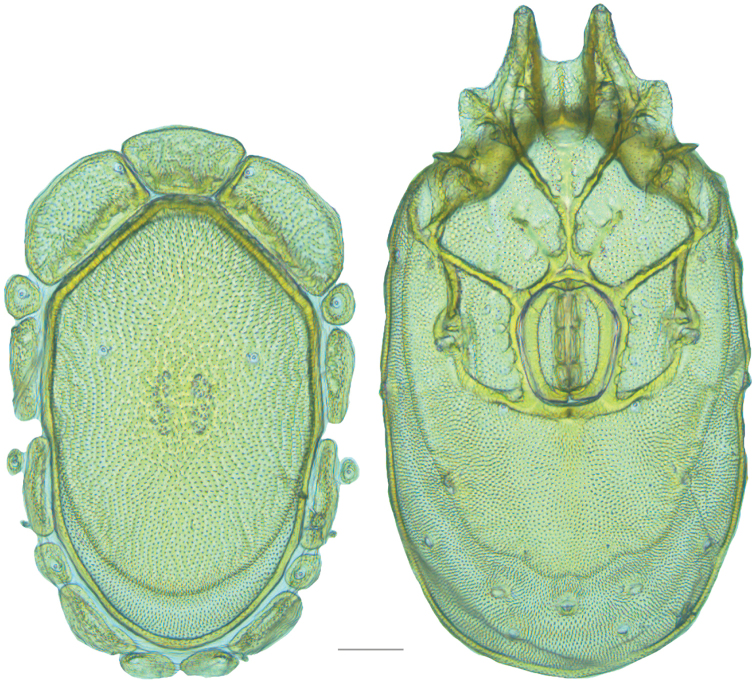
*Testudacarus oblongatus* female: (**Left**) dorsum; (**Right**) venter. Scale: 100 µm.

Dorsum (Fig. 47) — [826–915 long; 539–623 wide] oblong and colorless. Dorsal plate [695–779 long; 446–449 wide]. Primary sclerotization [617–701 long]. Dorso-glandularia-4 [188–280 apart] slightly anterior to [0–26] and well lateral to [42–82] muscle scars. Platelets colorless. Anterio-medial platelet [182–211 long; 101–120 wide] trapeziform to nearly triangular (posterior margin strongly shortened). Anterio-lateral platelets [213–244 long; 111–135 wide] near rectangular and without noticeable bump. Lateral platelets as follows: lateral-1 [60–80 long; 46–54 wide]; lateral-2 [149–180 long; 39–50 wide]; lateral-3 [30–50 long; 18–28 wide]; lateral-4 [158–193 long; 33–46 wide]; lateral-5 [44–72 long; 25–48 wide]; lateral-6 [128–142 long; 31–53 wide]; lateral-7 [52–89 long; 25–40 wide].

Venter (Fig. 47) — [1022–1095 long; 586–664 wide] oblong. Primary sclerotization [860–947 long] extensive. Gnathosomal bay [74–109 dorsal length; 176–190 ventral length; 78–116 wide]. Coxal field [603–632 long; 424–507 wide]. Coxa-I [288–319 long; 112–137 midlength]. Coxa-II + III [123–136 distance to top of coxa-II; 222–238 distance to top of coxa-III; 413–456 distance to bottom of coxa-III; 298–330 total length] extensive. Coxa-IV [385–425 distance to top; 196–218 total length]. Genital field [415–446 distance to top; 618–656 distance to bottom; 196–210 total length; 155–178 width; 239–267 distance from gnathosomal bay; 117–137 distance from coxa-I; 278–335 distance to excretory pore; 402–452 distance to caudad]. Eggs [173–175 long; 1–2 eggs]. Distance to excretory pore [902–983].

Legs — colorless. Total leg and podomere lengths as follows: Leg-I [623–676 total; trochanter 72–85; basifemur 106–115; telofemur 85–94; genu 114–129; tibia 123–137; tarsus 109–120]. Leg-II [642–689 total; trochanter 75–80; basifemur 108–117; telofemur 88–94; genu 115–135; tibia 131–152; tarsus 119–133]. Leg-III [710–777 total; trochanter 70–80; basifemur 106–126; telofemur 92–100; genu 129–151; tibia 151–172; tarsus 146–161]. Leg-IV [941–1001 total; trochanter 106–125; basifemur 135–150; telofemur 139–146; genu 188–199; tibia 197–215; tarsus 160–178].

**Male (n=9)** similar to female except for sexually dimorphic characters previously discussed and with following specifications.

Gnathosoma — Subcapitulum [153–177 ventral length; 99–118 dorsal length; 98–110 tall]. Chelicerae [123–148 long]. Fangs [28–32 long]. Pedipalp [201–231 long]. Trochanter [29–33 long; 27–30 wide]. Femur [51–55 long; 37–46 wide]. Genu [44–55 long; width 32–37 wide]. Tibia [54–66 long; 20–23 wide]. Tarsus [19–22 long; 10–12 wide].

**Figure 48.**
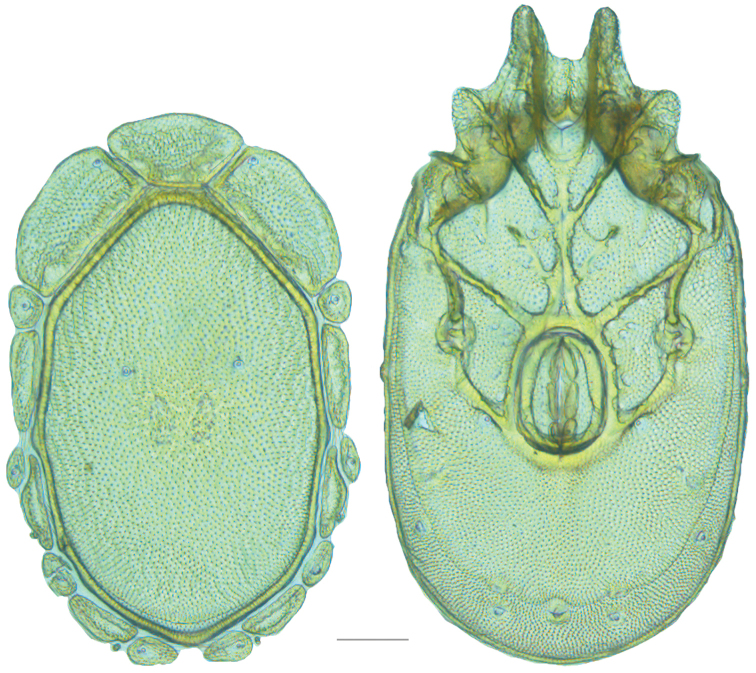
*Testudacarus oblongatus* male: (**Left**) dorsum; (**Right**) venter. Scale: 100 µm.

Dorsum (Fig. 48) — [683–775 long; 405–496 wide]. Dorsal plate [566–648 long; 356–437 wide] occasionally with minute area of secondary sclerotization. Dorso-glandularia-4 [139–231 apart] roughly equal distance anterior to [22–85] and lateral to [25–60] muscle scars. Anterio-medial platelet [156–186 long; 90–119 wide]. Anterio-lateral platelets [180–213 long; 87–110 wide]. Lateral platelets as follows: lateral-1 [44–60 long; 32–50 wide]; lateral-2 [105–161 long; 25–42 wide]; lateral-3 [33–70 long; 18–27 wide]; lateral-4 [105–150 long; 25–41 wide]; lateral-5 [44–65 long; 28–41 wide]; lateral-6 [85–105 long; 29–40 wide]; lateral-7 [54–76 long; 25–39 wide].

Venter (Fig. 48) — [809–936 long; 432–551 wide]. Primary sclerotization [724–863 long]. Gnathosomal bay [69–88 dorsal length; 133–168 ventral length; 59–99 wide]. Coxal field [491–577 long; 331–424 wide]. Coxa-I [235–279 long; 102–117 midlength]. Coxa-II + III [100–120 distance to top of coxa-II; 178–210 distance to top of coxa-III; 365–432 distance to bottom of coxa-III; 265–315 total length]. Coxa-IV [323–381 length to top; 168–202 total length]. Genital field [381–458 distance to top; 550–636 distance to bottom; 161–185 total length; 119–130 width; 248–298 distance from gnathosomal bay; 146–187 distance from coxa-I; 174–241 distance to excretory pore; 250–314 distance to caudad]. Genital skeleton [225–255 long; 123–152 wide]. Distance to excretory pore [724–863].

Legs — total leg and podomere lengths as follows: Leg-I [526–617 total; trochanter 57–69; basifemur 90–103; telofemur 75–90; genu 100–116; tibia 105–124; tarsus 97–116]. Leg-II [536–629 total; trochanter 59–70; basifemur 87–106; telofemur 73–86; genu 100–117; tibia 106–134; tarsus 104–123]. Leg-III [589–691 total; trochanter 60–73; basifemur 89–111; telofemur 71–90; genu 115–130; tibia 123–151; tarsus 128–147]. Leg-IV [810–878 total; trochanter 92–101; basifemur 112–125; telofemur 114–127; genu 144–177; tibia 158–188; tarsus 143–168]

##### Etymology.

Specific epithet *oblongatus* (*oblong*-, L. rather long) referring to the oblong idiosoma.

##### Distribution.

West coast of British Columbia, Washington, Oregon, and California.

### Asian species

#### Testudacarus tripeltatus

Taxon classificationAnimaliaTrombidiformesTorrenticolidae

Walter, 1928

http://zoobank.org/3871B946-C254-4A5E-81A2-2B4E45E5A47F

Testudacarus tripeltatus : Walter 1928: 62, 64, 75–77; Walter 1929: 217, 220; Marshall 1943: 318, 320, 322; Radford 1950: 120; Baker and Wharton 1952: 295; Mitchell 1954: 40; Imamura 1955: 182, 188; Viets 1956: 256; Cook 1967: 5; Lundblad 1967: 418; Cook 1974: 146; Prasad 1974: 50–52, 186, 235; Imamura 1976: 283–284; Viets 1987: 724; Cramer 1992: 14; Wiles 1997a: 199, 201, 209; Wiles 1997b: 1245; Pešić and Smit 2007: 49–50; Pešić et al. 2010: 15.

##### Type series.

**Holotype (1♀): Himachal Pradesh, India**: (1♀) from Kangra Valley, Upper Dharamsala, Bhagsunath, June 4^th^ 1926, by Dr Hora.

##### Type deposition.

Holotype (1♀) at Naturhistorisches Museum Basel, Switzerland.

##### Diagnosis.

*Testudacarus tripeltatus* can be differentiated from all other Asian species by distribution (India, Java, and Bhutan) and large size (dorsal length >700 µm). More research and updated descriptions are needed for a better diagnosis.

##### Distribution.

India (Walter 1928), Java (Walter 1929), and Bhutan (Pešić and Smit 2007).

#### Testudacarus japonicus

Taxon classificationAnimaliaTrombidiformesTorrenticolidae

Imamura, 1955

http://zoobank.org/F3B6396B-0BEB-475E-B83D-83B6AF9C2D71

Testudacarus japonicus : Imamura 1955: 182, 186–187; Imamura 1965: 238; Lundblad 1967: 418; Imamura 1976: 283–284; Imamura 1980: 343; Imamura 1986: 381; Viets 1987: 724; Wiles 1997a: 201, 209; Abé 2005: 120; Abé 2006: 6; Abé et al. 2006: 14.

##### Type series.

**Holotype (1♂): Shizuoka, Japan**: brook connected with a stream in Takékura, Mishima, Shizuoka, Japan, 15 May 1953, by T. Imamura.

The types were not examined for this publication.

##### Type deposition.

Holotype (1♂) at Taiji Imamura Collection at Ibaraki Nature Museum, Japan.

Holotype loans are not available from Ibaraki Nature Museum. The museum provided a low-magnification photograph through e-mail, though permission to print the photograph was not obtained.

##### Diagnosis.

These mites differ from all other Testudacarinae by distribution (Japan), and from *Testudacarus tripeltatus* by small size (dorsal length <700 µm). *Testudacarus japonicus* may be conspecific with *Testudacarus okadai*. More research and updated descriptions are needed for a better diagnosis.

##### Distribution.

Takékura, Japan (Imamura 1955).

##### Remarks.

It is reasonable to assume Imamura (1955) had no knowledge of Habeeb (1954) because he never mentions *Testudacarus vulgaris* and there are inaccuracies in his description that could have been prevented if he had. Firstly, his “female” type specimen is almost certainly a male as “the genital area [is] relatively more to the posterior than in [females] and the two [dorsal muscle scars]… are located posterior to the [glandularia]” (Habeeb 1954). Furthermore, in his remarks he states the “Japanese specimen resembles most the Indian species,” which with more current information is unlikely. At the time, *Testudacarus japanicus* would have been most similar in size, color, and shape to either *Testudacarus vulgaris* or *Testudacarus minimus*, not *Testudacarus tripeltatus*. Most importantly, the *Testudacarus japonicus* type is almost certainly male and therefore shares little morphology with the female *Testudacarus tripeltatus*. Therefore, the distinctions Imamura (1955) offers that *Testudacarus japonicus* are “different from [*Testudacarus tripeltatus*] in the anterior tips of the first [coxae], [pedi]palps, situations of [coxae] and genital organ” are unhelpful (Imamura 1955). He is referring to sexual dimorphism and comparing only the two most disparate species available to him.

#### Testudacarus okadai

Taxon classificationAnimaliaTrombidiformesTorrenticolidae

Imamura, 1976

http://zoobank.org/B66EFC5E-2C6A-448A-91E8-C5B1D7922654

Testudacarus okadai : Imamura 1976: 279, 281–284; Imamura 1980: 342–343; Viets 1987: 724; Wiles 1997a: 201, 209; Abé 2005: 120; Abé 2006: 6; Abé et al. 2006: 14; Pešić and Smit 2007: 50.

##### Type series.

**Holotype (1♀): Tichigi, Japan**: Onisawa, Shôbuga-Hama, Nikkô National Park, 13 May 1974, by Y. Okada.; **Allotype (1♂?): Tichigi, Japan**: Onisawa, Shôbuga-Hama, Nikkô National Park, 13 May 1974, by Y. Okada.

The types were not examined for this publication.

##### Type deposition.

Holotype (1♀) and allotype (1♂?) at Taiji Imamura Collection at Ibaraki Nature Museum, Japan.

Holotype loans are not available from Ibaraki Nature Museum. The museum provided a low-magnification photograph through e-mail, though permission to print the photograph was not obtained.

##### Diagnosis.

These mites differ from all other Asian Testudacarinae by distribution (Japan), and from *Testudacarus tripeltatus* by small size (dorsal length <700 µm). *Testudacarus okadai* may be conspecific with *Testudacarus japonicus*. More research and updated descriptions are needed for a better diagnosis.

##### Distribution.

Throughout Honshu, Japan (Imamura 1980).

##### Discussion.

A drawing of the “male” dorsum is left out of the *Testudacarus okadai* description. This is of the utmost importance because the sex of the “male” specimen is in question. The positioning of the genital field in relation to coxae-IV and the short coxae-II+III midline is typical of female testudacarines, but the coxal field size in relation to the venter is typical of males (Fig. 7). Furthermore, Imamura states the “feature and shape of dorsal shields are all similar to those of the female” (Imamura 1976). Again, testudacarine male and female dorso-glandularia-4 are positioned differently with respect to the muscle scars. While his word choice of “similar” suggests this difference could exist, without a more elaborate description or a drawing it is impossible to tell (Imamura 1976, 1980). In short, it is possible that this is an atypically small female, or a teneral female that has not undergone secondary growth and sclerotization. Imamura (1976) continues to confuse sexual dimorphism when he states: “the female of *okadai* n. sp. is also clearly distinguished from… *japonicus*… by the feature of the venter.” Although this is true, it is because one is female and the other male. This casts suspicion on *Testudacarus okadai*. Imamura (1976) seems to be suggesting they are separate species based on his confusions about sexual differences. *Testudacarus okadai* could be synonymous with *Testudacarus japonicus* and this issue should be further explored. Wiles (1997a) offers a key to Asian species, but the characters he used to differentiate species are also differences between sexes and therefore are not useful.

#### Testudacarus binodipalpis

Taxon classificationAnimaliaTrombidiformesTorrenticolidae

Guo & Jin, 2005

http://zoobank.org/0AF046B5-9FA7-4DA2-9C08-7051076DA7AC

Testudacarus binodipalpis : Guo and Jin 2005: 70; Jin et al. 2010: 111.

##### Type series.

**Holotype (1♀): Guizhou, China**: Mt. Fanjing (27°49'–28.01'N, 108°46'–108°49'E), 29 July 2001, by Guo Jian-Jun, 2001-VII-291; **Paratype (1♀): Guizhou, China**: Mt. Fanjing (27°49'–28.01'N, 108°46'–108°49'E), 4 Aug 2001, by Guo Jian-Jun, 2001-VII-292.

The types were not examined for this publication; contact with the authors was attempted but unsuccessful and the the types were not examined.

##### Type deposition.

Institute of Entomology , Guizhou University.

##### Diagnosis.

These mites can differ from all other Testudacarinae by distribution (China) and from *Testudacarus tripeltatus* by their small size (dorsal length <700 µm). More research and updated descriptions are needed for a better diagnosis.

##### Distribution.

Mt. Fanjing (Guo and Jin 2005) and Fujian, China (Jin et al. 2010).

##### Remarks.

*Testudacarus binodipalpis* was described from one female and one “male.” The described “male” is almost certainly a female as it exhibits all female sexual characters and no ejaculatory complex is noted in the description. However, these two females differ in some noteworthy respects. From illustrations it appears that the smaller female seems to have undergone tertiary sclerotization, while the larger female seems to have only undergone primary and secondary sclerotization. The size and positioning of lateral platelets are also quite different in each specimen. For these reasons the specimens should be re-examined as they might represent two species diagnosable by size. Guo and Jin (2005) state that *Testudacarus binodipalpis* can be separated from other *Testudacarus* by “the possession of 2 tubercles on the ventral surface of the” pedipalp tibia and the genu and femur “both with a feathered seta on the ventral surface.” These pedipalp characters do not work as they are plesiomorphic for all *Testudacarus* (Fig. 5). Guo and Jin (2005) also state that the “dorsal and ventral apodeme both [have] a round terminal tip; [coxae-IV] with a triangular base.” These additional characters are unhelpful in separating any testudacarines.

### Key to world species of Testudacarinae

**Table:** 

1	Pedipalp four-segmented, anterior tip of coxa-I with projection; California	***Debsacarus oribatoides***
–	Pedipalp five-segmented, anterior tip of coxa-I without projection	**2**
2	Body elongate to rectangular	(*Testudacarus elongatus* complex)...**3**
–	Body oval	**5**
3	Lateral platelets robust; lateral-platelet-4 large and in direct or near direct contact with lateral-platelet-2	**4**
–	Lateral-platelet-4 small, gap present between it and lateral-platlet-2; widespread along west coast of N. America	***Testudacarus oblongatus***
4	Distribution restricted to the Olympic Mountains	***Testudacarus rectangulatus***
–	Distribution within and east of the Cascade Mountains	***Testudacarus elongatus***
5	Body large (>700 µm female and >650 male dorsal length; if smaller, found in Asia), dull coloration common; within and west of the Rocky Mountains or Asia	(Asian species and *Testudacarus americanus* complex except *Testudacarus rollerae*)...**6**
–	Body small (<700 µm female and <650 male dorsal length), bright coloration (orange, red, violet, blue) common; present throughout North America	(*Testudacarus minimus* complex, *Testudacarus hitchensi* complex, *Testudacarus rollerae*)...**12**
6	Gnathosomal bay “covered” (short dorsal gnathosomal bay length), gnathosoma elongate with long rostrum that extends below ventral surface of gnathosoma	***Testudacarus hyporhynchus***
–	Without these characters	**7**
7	Body very large (>900 µm dorsal length, female) with small, square (unusual for complex) anterio-medial platelet (male unknown)	***Testudacarus kirkwoodae***
–	Body not this large (<900 µm dorsal length, female and male) often with wide, rectangular or trapezoidal anterio-medial platelet	**8**
8	Anterior-medial platelet compact and pentagonal, suture lines between second and third coxae absent; India, Java, Bhutan	***Testudacarus tripeltatus***
–	Anterior-medial platelet wider, more trapezoidal in shape, suture lines between second and third coxae present, but incomplete	**9**
9	Body small (<650 µm dorsal length female and <450 µm dorsal length male); Japan	***Testudacarus japonicas* or *Testudacarus okadai***
–	Body larger (>700 µm dorsal length female and >500 µm dorsal length male)	**10**
10	Body <770 µm dorsal length female and <660 µm dorsal length male, dg-4 in line with middle of dorsal muscle scars; China	***Testudacarus binodipalpis***
–	Body >780 µm dorsal length female and >670 µm dorsal length male, dg-4 anterior to dorsal muscle scars	**11**
11	Body elliptical, colorless to peach, small cheliceral fang (<33 µm)	***Testudacarus americanus***
–	Body rounded, grey to colorless, large cheliceral fang (>40 µm)	***Testudacarus smithi***
12	Anterio-medial platelet wide (>140 µm) and more than or nearly twice as wide as long	***Testudacarus rollerae***
–	Anterio-medial platelet unmodified (<140 µm) and less than twice as wide as long	(*Testudacarus minimus* and *Testudacarus hithensi* complex)...**13**
13	Anterio-medial and anterio-lateral platelets with consistent coloration (either colorless or colorled across)	(*Testudacarus minimus* complex)...**14**
–	Anterio-lateral platelets with coloration and anterio-medial platelet colorless	**18**
14	Body in entirety conspicuously violet; currently known only from Arkansas	***Testudacarus radwellae***
–	Body orange, red, pink, blue, or violet but not covering the majority of body	**15**
15	Distribution east of the Great Plains, color violet to blue	***Testudacarus vulgaris***
–	Distribution within and west of the Great Plains	**16**
16	Distribution in Washington or northern Oregon, orange to red	***Testudacarus minimus***
–	Distribution in and west of Great Plains excluding Washington and northern Oregon	**17**
17	Violet to blue, females and males smaller than 600 and 500 µm, respectively	***Testudacarus vulgaris*** (most likely)
–	Collected outside of California, orange to red to clear, females and males larger than 600 and 500 µm, respectively	***Testudacarus minimus*** (most likely)
–	Collected in California and orange to clear	***Testudacarus minimus*, *vulgaris*, or *deceptivus*** (no morphological characters for reliable identification available within California)
18	Dorsal plate with large medial pores surrounded by distal ring of smaller pores (all pores uniform in other species), area posterior to coxal plate “bleached” in males	***Testudacarus hitchensi***
–	Dorsal plate with uniform pores, males without “bleached” area	**19**
19	Anterio-lateral platelets with violet to blue coloration covering most of platelet	***Testudacarus harrisi***
–	Anterio-lateral platelets with coloration restricted to the posterior half of platelet	**20**
20	Females and males with dorsal lengths less than 575 and 450 µm, respectively	***Testudacarus dennetti***
–	Females and males with dorsal lengths greater than 600 and 475 µm, respectively	***Testudacarus dawkinsi***

## Supplementary Material

XML Treatment for Torrenticolidae

XML Treatment for Testudacarinae

XML Treatment for Debsacarus

XML Treatment for Debsacarus oribatoides

XML Treatment for Testudacarus

XML Treatment for Testudacarus minimus

XML Treatment for Testudacarus vulgaris

XML Treatment for Testudacarus deceptivus

XML Treatment for Testudacarus radwellae

XML Treatment for Testudacarus hitchensi

XML Treatment for Testudacarus harrisi

XML Treatment for Testudacarus dennetti

XML Treatment for Testudacarus dawkinsi

XML Treatment for Testudacarus americanus

XML Treatment for Testudacarus kirkwoodae

XML Treatment for Testudacarus hyporhynchus

XML Treatment for Testudacarus smithi

XML Treatment for Testudacarus rollerae

XML Treatment for Testudacarus elongatus

XML Treatment for Testudacarus rectangulatus

XML Treatment for Testudacarus oblongatus

XML Treatment for Testudacarus tripeltatus

XML Treatment for Testudacarus japonicus

XML Treatment for Testudacarus okadai

XML Treatment for Testudacarus binodipalpis

## References

[B1] Abé H (2005) Annotated checklist of Japanese water mites (Acari: Prostigmata: Hydracarina). Acta Arachnologica 54: 111–145. doi: 10.2476/asjaa.54.111

[B2] Abé H (2006) A catalogue of Japanese water mites (Acari: Prostigmata: Hydracarina). The Acarological Society of Japan 15: 1–16. doi: 10.2300/acari.15.1 [In Japanese]

[B3] Abé H Imamura T Kikuchi Y (2006) Catalogue of type specimens of aquatic mites (Acari, Hydrachnellae and Halacaridae) in the Taiji Imamura collection of Ibaraki Nature Museum, Ibaraki, Japan. Bulletin of Ibaraki Nature Museum 9: 1–18.

[B4] Bader C (1988) Die Torrenticolidae (Acari, Hydrachnaellae). Eine abklärende studie über eine schwierige Wassermilben-Familie. Revue Suisse de Zoologie 95: 87–98. doi: 10.5962/bhl.part.79640

[B5] Baker EW Wharton GW (1952) An Introduction to Acarology. The Macmillan Company, New York, 465 pp.

[B6] Barr DW (1972) The ejaculatory complex in water mites (Acari: Parasitengona): morphology and potential value for systematics. Life Sciences Contributions of the Royal Ontario Museum 81: 1–87.

[B7] Barr DW (1977) A new water mite genus from western Canada (Acari: Parasitengona: Anisitsiellidae). Canadian Journal of Zoology 55: 877–881. doi: 10.1139/z77-114

[B8] Barr DW (1982) Comparative morphology of the genital acetabula of aquatic mites (Acari, Prostigmata): Hydrachnoidea, Eylaoidea, Hydryphantoidea and Lebertioidea. Journal of Natural History 16: 147–160. doi: 10.1080/00222938200770111

[B9] Bergstrom D (1953) Hydracarina from the Rocky Mountain Region. Transactions of the American Microscoicpal Society 72: 157–162. doi: 10.2307/3223514

[B10] Boyaci YÖ Özkan M (2008) The species of the genus *Monatractides* Viets, 1926 (Acari, Hydrachnidia, Torrenticolidae) in Turkey. Turkish Journal of Zoology 32: 363–366.

[B11] Chakrabarty P Warren M Page LM Baldwin CC (2013) GenSeq: An updated nomenclature and ranking for genetic sequences from type and non-type sources. ZooKeys 346: 29–41. doi: 10.3897/zookeys.346.5753 24223486 10.3897/zookeys.346.5753 PMC3821064

[B12] Conroy JC (1968) The water-mites of western Canada. Bulletin of the National Museum of Canada 223: 23–42.

[B13] Conroy JC Scudder GGE (1975) An annotated checklist of the water mites (Acari) of British Columbia. Syesis 8: 305–310.

[B14] Cook DR (1967) Water mites from India. Memoirs of the American Entomological Institute 9: 1–411.

[B15] Cook DR (1974) Water mite genera and subgenera. Memoirs of the American Entomological Institute 21: 1–841.

[B16] Cramer C (1992) Estudios sobre hidracáridos Mexicanos, familia Torrenticolidae. I. Cinco especies nuevas de *Neoatractides* y *Torrenticola* y primer registro de *Testudacarus* para México. Anales Instituto de Biologia, Universidad Nacionale Autónoma de México, Seria Zoologia 63: 13–27. [In Spanish]

[B17] Cramer C Cook DR (2000) Water mites of the genera *Neoatractides* Lundblad and *Pseudotorrenticola* Walter (Acari: Hydrachnida: Torrenticolidae) from Mexico. International Journal of Acarology 26: 51–61. doi: 10.1080/01647950008683635

[B18] Crowell RM (1961) Catalogue of the distribution and ecological relationship of North American Hydracarina. Canadian Entomologist 93: 321–359. doi: 10.4039/Ent93321-5

[B19] Cuellar D Underwood DLA (2012) Final report wadeable streams bioassessment region 8 sites samples: May-July 2009. Prepared by California State University Long Beach Stream Ecology and Assessment Laboratory for Santa Ana Regional Water Quality Control Board, 73 pp.

[B20] Davids C di Sabatino A Gerecke R Gledhill T Van der Hammen H Smit H (2007) Acari: Hydrachnidia. In: Gerecke R (Ed.) Chelicerata: Araraneae, Acari I. Süßwasserfauna von Mitteleuropa, vol. 7/2–1 Elsevier Spektrum Akademischer Verlag, München, Germany, 241–376.

[B21] Esen Y Erman O (2014) Kahramanmaraş İli *Monatractides* K. Viets, 1926 ve *Torrenticola* Piersig, 1896 (Acari: Hydrachnidia: Torrenticolidae) Türleri ve Türkiye faunası için İki yeni kayıt. Firat University Journal of Science 26: 49–44. [In Turkish]

[B22] Fernández HR Reid B (2012) Invertebrate distribution on a macroalgae/macrophyte mixed mat in flowing water. Fundamental and Applied Limnology 181: 289–299. doi: 10.1127/1863-9135/2012/0373

[B23] Fisher JR Fisher DM Nelson WA O’Neill JC Skvarla MJ Radwell AJ Ochoa R Bauchan G Dowling APG (2015) An integrative description of *Torrenticola trimaculata* sp. nov. (Parasitengona: Torrenticolidae), a three-spotted, riffle- dwelling mite from eastern North America: morphology, phylogenetics, and taxonomic history of the genus. Acarologia 55: 71–116. doi: 10.1051/acarologia/20152155

[B24] Folmer O Black M Hoeh W Lutz R Vrijenhoek R (1994) DNA primers for amplification of mitochondrial cytochrome c oxidase subunit I from diverse metazoan invertebrates. Molecular Marine Biology and Biotechnology 3: 294–299. 7881515

[B25] Fuste LA (1980) Effects of the Mount St. Helens eruption on the benthic fauna of the Toutle River, Muddy River, and Pine Creek Drainage Basins, Washington. Geological Survey Circular 850-H, 19 pp.

[B26] GEI (2008) Evaluation of potential site-specific zinc, cadmium, and copper standards for the Eagle River, segment 5 and Cross Creek, segment 7b: technical memorandum. Prepared by GEI Consultants, Inc. Ecological Devision, Littleton, Colorado for CBS Operations, 101 pp.

[B27] Goldschmidt T (2007) Studies on Latin American water mites of the genus *Torrenticola* Piersig, 1896 (Torrenticolidae, Hydrachnidia, Acari). Zoological Journal of the Linnean Society 150: 443–678. doi: 10.1111/j.1096-3642.2007.00305.x

[B28] Guo J-J Jin D-C (2005) Description of a new species *Testudacarus* in the subfamily Testudacarinae newly recorded from China (Acari, Lebertioidea, Torrenticolidae). Acta Zootaxonomica Sinica 30: 70–72.

[B29] Guo J-J Zhang P (2011) Cladistics and phylogeny of genus (subgenus) relationships within *Torrenticolidae*. Journal of Southwest University (Natural Science Edition) 33: 46–49. [In Chinese]

[B30] Habeeb H (1954) North American Hydrachnellae, Acari – IX-XVI. Leaflets of Acadian Biology 2: 1–14.

[B31] Habeeb H (1956) Notes on water-mites – I. Leaflets of Acadian Biology 11: 1–2.

[B32] Habeeb H (1959a) List of North American water-mites. Le Naturaliste Canadien 86: 19–25.

[B33] Habeeb H (1959b) New Hydrachnellae chiefly from California. Leaflets of Acadian Biology 19: 1–6.

[B34] Habeeb H (1961) Walter Vincent Powers, noble fellow, 1895-1954. Leaflets of Acadian Biology 22: 1–6.

[B35] Habeeb H (1967) A check list of North American water-mites. Leaflets of Acadian Biology 43: 1–8.

[B36] Habeeb H (1969) Notes on water-mites – II. (erratum: III). Leaflets of Acadian Biology 51: 1–2.

[B37] Habeeb H (1974a) New genera of water-mites. Leaflets of Acadian Biology 61: 1–2.

[B38] Habeeb H (1974b) Notes on water-mites. VIII. Leaflets of Acadian Biology 59: 1–4.

[B39] Hall TA (1999) BioEdit: a user-friendly biological sequence alignment editor and analysis program for Windows 95/98/NT. Nucleic Acids Symposium 41: 95–98.

[B40] Harvey MS (1998) The Australian Water Mites: A Guide to Families and Genera. CSIRO Publishing, Collingwood, 150 pp.

[B41] Hawkins CP (2009) Revised invertebrate RIVPACS model and O/E index for assessing the biological condition of Colorado streams. Prepared by Western Center for Monitoring and Assesment of Freshwater Ecosystems, Department of Watershed Sciences, Utah State University for Colorado Department of Public Health and Environment, Water Quality Control Division–Monitoring Unit, 42 pp.

[B42] Herbst DB Medhurst RB Bell ID (2013) Evaluating recovery of stream invertebrate communities following removal of introduced trout in Kings Canyon National Park: baseline biological stream surveys and contrasts after fish removal. Sierra Nevada Aquatic Research Laboratory, University of California, Mammoth Lakes, California, 31 pp.

[B43] Herbst DB Medhurst RB Roberts SW (2011) Development of biological criteria for sediment TMDLs: the relation of sediment deposition to benthic invertebrate communities of streams exposed to varied land use disturbances in the Sierra Nevada and Coast Range mountains of California. Prepared by Sierra Nevada Aquatic Research Laboratory for California State Water Resources Control Board, 31 pp.

[B44] Herbst DB Roberts SW Medhurst RB Hayden NG (2011) Sediment TMDL guidance for Central Coast Region of California and the San Lorenzo River: physical habitat and biological criteria for deposited sediments in streams, 24 pp.

[B45] Herbst DB Silldorff EL (2009) Development of a benthic macroinvertebrate index of biological integrity (IBI) for stream assessments in the Eastern Sierra Nevada of California. Prepared by Sierra Nevada Aquatic Research Laboratory and Delaware River Basin Commission, 89 pp.

[B46] Herbst D Roberts S Medhurst B Bogan M (2010) A Sentinel monitoring network for detecting the hydrologic effects of climate change on Sierra Nevada headwaters stream ecosystems and biological indicators. Sierra Nevada Aquatic Research Labratory, University of California, 26 pp.

[B47] Imamura T (1955) Crenophilous and rheophilous water-mites from Mishima and its vicinity. Bulletin of the Biogeographical Society of Japan 16-19: 181–192.

[B48] Imamura T (1965) Hydrachnellae. In: Sasa M (Ed.) Mites, an introduction to classification, bionomics and control of Acarina. University of Tokyo Press, Tokyo, Japan, 216–251. [In Japanese]

[B49] Imamura T (1976) Two new species of water-mites from Nikko National Park, Japan. Annotationes Zoologicae Japonenses 49: 279–284.

[B50] Imamura T (1980) Hydrachnellae. In: Ehara S (Ed.) Illustrations of the mites and ticks of Japan. Zenokoku Noson Kyoiku Kyokai, Tokyo, 330–379. [In Japanese]

[B51] Imamura T (1986) Acarina: Hydrachnellae. In: Ueno M (Ed.) Freshwater Biology of Japan. Hokuryukan, Tokyo, 368–395.

[B52] Jin D-C Yi T-C Guo J-J (2010) A review of progress in taxonomy of water mites from China (Acari: Hydrachnidia). In: Zhang Z-Q Hong X-Y Fan Q-H (Eds) Xin J-L, Centenary: Progress in Chinese Acarology. Zoosymposia 4: 106–119.

[B53] Laubitz DR Sutherland I Sharma N Antoine W (1983) 1 Bibliographia invertebratorum aquaticorum canadensium, vol. 1. Invertebrate Zoology Division National Museum of Natural Sciences, Ottawa, 56 pp.

[B54] Lewis WM McCutchan JH (2005) Environmental thresholds for nutrients in streams and rivers of the Colorado mountains and foothills. USEPA Project number: X7-97805801, 87 pp.

[B55] Lundblad CO (1967) Wassermilben von Hinterindien. Arkiv för Zoologi 19: 391–419. [In German]

[B56] Lundblad O (1941) Eine übersicht des Hydrachnellensystems und der bis jetzt bekannten Verbreitung der Gattungen dieser Gruppe. Zoologiska Bidrag från Uppsala 20: 359–379. [In German]

[B57] Marshall R (1943) Hydracarina from California. Part I. Transactions of the American Microscopical Society 62: 306–324. doi: 10.2307/3223036

[B58] ME Inc. (2011) Evaluation of study design alternatives for benthic invertebrate community assessment at United Keno Hill Mines (DRAFT). Prepared by Minnow Environmental Inc., Georgetown, Ontario, Canada for Elsa Recplamation and Development Company Ltd., Whitehorse, 25 pp.

[B59] Miller MA Pfeiffer W Schwartz T (2010) Creating the CIPRES Science Gateway for inference of large phylo- genetic trees. Proceedings of the Gateway Computing Environments Workshop. New Orleans, Louisiana, 1–8.

[B60] Mitchell RD (1954) Check list of North American water-mites. Fieldiana: Zoology 35: 27–70.

[B61] Mitchell RD (1962) The structure and evolution of water-mite mouthparts. Journal of Morphology 110: 41–59.

[B62] MMWD (2008) Final report: consolidated concept proposal for nonpoint source projects, Greater San Pablo Bay Area grant agreement no. 04-155-552-2. Prepared by Marin Municipal Water District, Corte Madera, 71 pp.

[B63] Morimoto S (2012) Jewelry in the water. Symbiosis 7: 85–87. [In Japanese]

[B64] Newell IM (1959) Acari. In: Edmonson WT (Ed.) Fresh-Water Biology (2nd Ed). John Wiley & Sons Inc, New York, 1080–1116.

[B65] O’Neill JC (2015) Systematics of testudacarine torrent mites (Parasitengona, Hydrachnidiae, Torrenticolidae). Master’s Thesis, University of Arkansas. 10.3897/zookeys.582.7684 PMC4857046 27199586

[B66] Park J O’Foighill D (2000) Sphaeriid and corbiculid clams represent separate heterodont bivalve radiations into freshwater environments. Molecular Phylogenetics and Evolution 14: 75–88. doi: 10.1006/mpev.1999.0691 10631043 10.1006/mpev.1999.0691

[B67] Peckarsky BI Fraissinet PR Penton MA Conklin DJ (1990) Freshwater macroinvertebrates of Northeastern North America. Cornell University Press, Ithica, 456 pp.

[B68] Pennak RW (1953) Fresh-water invertebrates of the United States. The Ronald Press Company, New York, 769 pp.

[B69] Pennak RW (1978) Fresh-water invertebrates of the United States. John Wiley and Sons Inc, New York, 803 pp.

[B70] Pennak RW (1989) Fresh-water invertebrates of the United States: Protozoa to Mollusca. John Wiley and Sons Inc, New York, 628 pp.

[B71] Pernot CP Underwood DLA (2010) Final report: wadeable streams bioassessment region 8 sites sampled: May – June 2008. Prepared by California State University Long Beach Stream Ecology and Assessment Laboratory, 73 pp.

[B72] Perrin CJ (2001) Trophic Structure and function in the Cheakamus River for water use planning. Prepared by Limnotek Research and Development Inc., Vancouver, Canada, for BC Hydro and The Resort Municipality of Whistler, 67 pp.

[B73] Perrin CJ (2006) Perophyton and benthic incertebrate monitoring for water use planning in the Coquitlam River, 2006. Prepared by Limnotek Research and Development Inc., Vancouver, 51 pp.

[B74] Perrin CJ Bennett SA (2011) Coquitlam River periphyton and benthic invertebrate monitoring: Coquitlam River Monitoring Program #5. Prepared by Limnotek Research and Development Inc. for BC Hydro, 73 pp.

[B75] Pešić V Chatterjee T Bordoloi S (2010) A checklist of the water mites (Acari: Hydrachnidia) of India, with new records and description of one new species. Zootaxa 2617: 1–54.

[B76] Pešić V Smit H (2007) First records of water mites (Acari: Hydrachnidia) from Bhutan, with descriptions of two new species. Zootaxa 1613: 45–56. 10.11646/zootaxa.4559.3.5 30791005

[B77] Prasad V (1974) A Catalogue of Mites of India. Indira Acarology Publishing House, Ludhiana, 320 pp.

[B78] Proctor HC (1992) The Evolution of Sperm Transfer Behaviour in Water Mites (Acari: Parasitengona). PhD Dissertation, University of Toronto, Toronto, 279 pp.

[B79] Proctor HC (2006) Key to Aquatic Mites Known from Alberta, 13 pp.

[B80] Proctor HC Smith IM Cook DR Smith BP (2015) Subphylum Chelicerata, Class Arachnida. In: Thorp JH Rogers DC (Eds) Thorp and Covich’s Freshwater Invertebrates: Ecology and General Biology. Academic Press, London, 599–660. doi: 10.1016/b978-0-12-385026-3.00025-5

[B81] De Queiroz K (1998) The general lineage concept of species, species criteria, and the process of speciation: A conceptual unification and terminological recommendations. In: Howard DJ Berlocher SH (Eds) Endless forms: species and speciation. Oxford University Press, Oxford, 57–75.

[B82] De Queiroz K (1999) The general lineage concept of species and the defining properties of the species category. In: Wilson RA (Ed.) Species: New Interdisciplinary Essays. MIT Press, Cambridge, Massachusetts, 49–89.

[B83] De Queiroz K (2005) A unified species concept and its consequences for the future of taxonomy. Proceedings of the California Academy of Sciences 56: 196–215.

[B84] De Queiroz K (2007) Species Concepts and Species Delimitation. Systematic Biology 56: 879–886. doi: 10.1080/10635150701701083 18027281 10.1080/10635150701701083

[B85] Radford CD (1950) Systematic check list of mite genera and type species. Union Internationale des Sciences Biologiques Série C, Section Entomologique 1: 1–232.

[B86] Richards AB Rogers DC (2006) List of Freshwater Macroinvertebrate Taxa from California and Adjacent States Including Standard Taxonomic Effort Levels. Southwest Association of Freshwater Invertebrate Taxonomists (SAFIT), 215 pp.

[B87] Richards AB Rogers DC (2011) List of Freshwater Macroinvertebrate Taxa from California and Adjacent States Including Standard Taxonomic Effort Levels. Southwest Association of Freshwater Invertebrate Taxonomists (SAFIT), 266 pp.

[B88] Smith IM (1982) Larvae of water mites of the genera of the superfamily Lebertioidea (Prostigmata: Parasitengona) in North America with comments on phylogeny and higher classification of the superfamily. Canadian Entomologist 114: 901–990. doi: 10.4039/Ent114901-10

[B89] Smith IM (1987) Order Acariformes, suborder Actinedida (or Prostigmata). In: Lafontaine JD Allyson S Behan-Pelletier VM Borkent A Campbell JM Hamilton KGA Martin JEH Masner L (Eds) The Insects, Spiders and Mites of Cape Breton Highlands National Park. Biosystematics Research Centre Report 1, 41–46.

[B90] Smith IM (1991a) Descriptions of new species representing new or unreported genera of Lebertioidea (Acari: Hydrachnida) from North America. Canadian Entomologist 123: 811–825. doi: 10.4039/Ent123811-4

[B91] Smith IM (1991b) Water mites (Acari: Parasitengona: Hydrachnida) of spring habitats in Canada. Memoirs of the Entomological Society of Canada 155: 141–167. doi: 10.4039/entm123155141-1

[B92] Smith IM (2010) Water mites (Acarina: Hydrachnidiae) of the Atlantic Maritime Ecozone. In: McAlpine DF Smith IM (Eds) Assessment of Species Diversity in the Atlantic Maritime Ecozone. Canadian Science Publishing (NRC Research Press), Ottawa, 283–311.

[B93] Smith IM Cook DR (1991) Water mites (Hydrachnidiae) and other arachnids. In: Thorp JH Covich AP (Eds) Ecology and Classification of North American Freshwater Invertebrates. Academic Press, San Diego, 523–592.

[B94] Smith IM Cook DR (1999) An assessment of global distribution patterns in water mites (Acari: Hydrachnida). In: Needham GR Mitchell RD Horn DJ Welbourn WC (Eds) Acarology IX (Vol. 2 Symposia). Ohio Biological Survey, Columbus, 109–124.

[B95] Smith IM Cook DR Smith BP (2010) Water mites (Hydrachnidiae) and other arachnids. In: Thorp JH Covich AP (Eds) Ecology and Classification of North American Freshwater Invertebrates. Academic Press, San Diego, 485–586. doi: 10.1016/B978-0-12-374855-3.00015-7

[B96] Smith IM Lindquist EE Behan-Pelletier V (2011) Mites (Acari). In: Scudder GGE Smith IM (Eds) Assessment of Species Diversity in the Montane Cordillera Ecozone, 193–268. Available at http://www.royalbcmuseum.bc.ca/assets/Montane-Cordillera-Ecozone.pdf

[B97] Smith IM Smith BP Cook DR (2001) Water mites (Hydrachnida) and other arachnids. In: Covich JH Thorp AP (Eds) Ecology and Classification of North American Freshwater Invertebrates. Academic Press, San Diego, 551–659. http://www.sciencedirect.com/science/article/pii/B978012690647950017X [April 10, 2015]

[B98] Stalingo DM (2009) Assessment of aquatic macroinvertebrates on USFS/BLM lands of the Crooked and Sage Creek watersheds. Prepared by the Montana Natural Heritage Program for Darin Watschke, USFS Custer National Forest, Billings, 26 pp.

[B99] Thompson JD Gibson TJ Pewniak F Jeanmougin F Higgins DG (1997) The Clustal X windows interface: flexible strategies for multiple sequence alignments aided by quality analysis tools. Nucleic Acids Research 24: 4876–4882. doi: 10.1093/nar/25.24.4876 9396791 10.1093/nar/25.24.4876 PMC147148

[B100] Viets K (1936) Wassermilben oder Hydracarina (Hydrachnellae und Halacaridae). In: Dahl F (Ed.) Die Tierwelt Deutschlands und der umgrenzenden Meeresteile (31/32). G. Fischer Verlag, Stuttgart, 1–574.

[B101] Viets K (1956) Die milben des süßwassers und des meeres: Hydrachnellae und Halacaridae (Acari). Zweiter un dritter teil. Katalog und nomenklator. Gustav Fischer, Jena, 870 pp.

[B102] Viets K (1935) Die wassermilben von Sumatra, Java und Bali nach den Ergebnissen der Deutschen Limnologischen Sunda-Expedition. II. Teil. Archiv für Hydrobiologie, Supplement 13: 595–738.

[B103] Viets KO (1987) Die milben des Süßwassers (Hydrachnellae und Halacaridae [part.], Acari). 2: Katalog. Sonderbände des Naturwissenschaftlichen Vereins in Hamburg 8: 1–1012. [In German]

[B104] Vitzthum H (1942) Acarina. In: Bronn HG (Ed.) Dr H.G. Bronn’s klassen und ordnungen des thierreichs, wissenschaftlich dargestellt in wort und bild. Bd. 5, Abt. 4, Buch 5. C.F. Winter (Akademie Verlag), Leipzig und Heidelberg [In German]

[B105] Walter C (1928) Zur kenntnis der mikrofauna von Britisch Indien. II. Hydracarina. Records of the Indian Museum 30: 57–108.

[B106] Walter C (1929) Hydracarinen aus Java. Treubia 11: 211–273.

[B107] Walter DE Lindquist EE Smith IM Cook DR Krantz GW (2009) Order Trombidiformes. In: Krantz GW Walter DE (Eds) A Manual of Acarology. Texas Tech University Press, Lubbock, 233–420.

[B108] Wiles PR (1997a) Asian and Oriental Torrenticolidae Piersig, 1902 (Acari: Hydrachnidia: Lebertioidea): a revision of the family and descriptions of ne species of *Torrenticola* Piersig and *Pseudotorrenticola* Walter, from Southeast Asia. Journal of Natural History 31: 191–236. doi: 10.1080/00222939700770121

[B109] Wiles PR (1997b) The homology of glands and glandularia in the water mites (Acari: Hydrachnidia). Journal of Natural History 31: 1237–1251. doi: 10.1080/00222939700770671

[B110] Williams DD Mundie JH Mounce DE (1977) Some aspects of benthic production in a salmonid rearing channel. Journal of the Fisheries Research Board of Canada 34: 2133–2141. doi: 10.1139/f77-280

[B111] Williams D Hogg ID (1988) Ecology and production of invertebrates in a Canadian coldwater spring-springbrook system. Holarctic Ecology 11: 41–54. doi: 10.1111/j.1600-0587.1988.tb00779.x

[B112] Winger PV Peters EJ Donahoo MJ Barnes JR White DA (1972) A checklist of the macroinvertebrates of the Provo River, Utah. Great Basin Naturalist 32: 211–219.

[B113] Young WC (1969) Ecological distribution of Hydracarina in north central Colorado. American Midland Naturalist 82: 367–401. doi: 10.2307/2423785

[B114] Zhang P Guo J-J (2010) Research progress on phylogeny of Torrenticolidae (Acari, Lebertioidea). Guizhou Agricultural Sciences 38: 116–118, 122.

